# Supplementary figures and images for: Effects of germline and somatic events in candidate BRCA-like genes on breast-tumor signatures
Source: PLoS One. 2020 Sep 30;15(9):e0239197. doi: 10.1371/journal.pone.0239197 (PMC7526916; doi:10.1371/journal.pone.0239197)

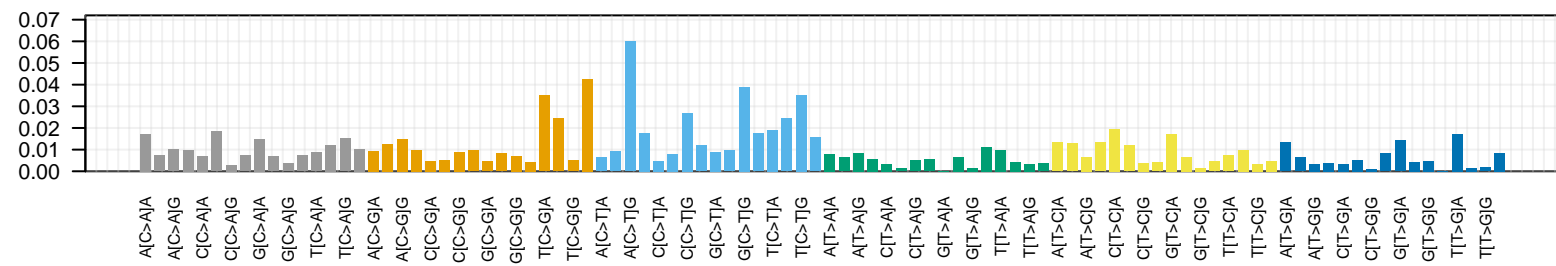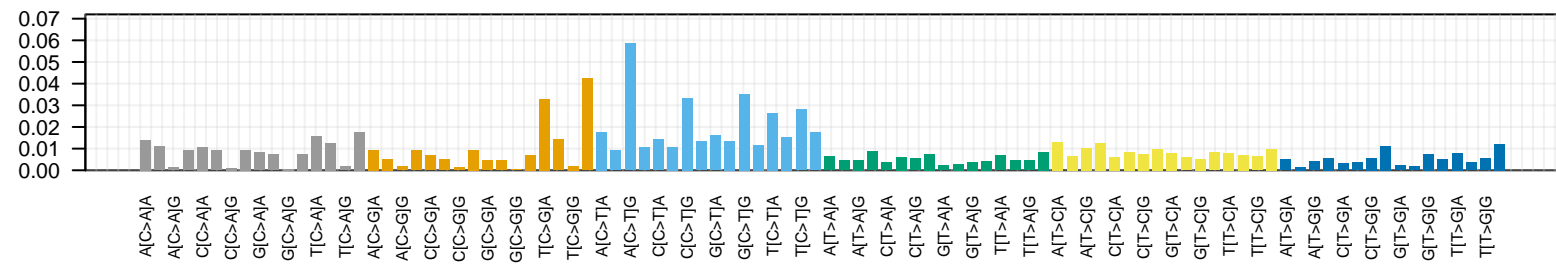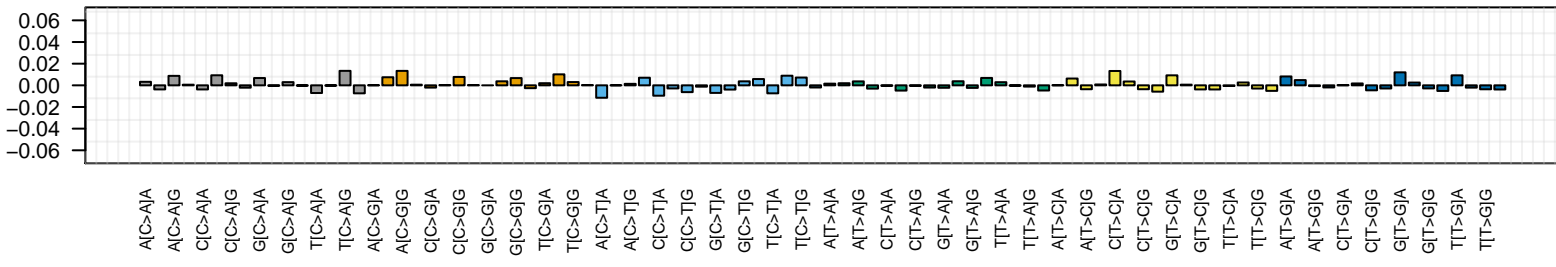

Supplement: S1 Fig — This tumor had a large proportion of C>T mutations, which are representative of Signature 3. (PDF) [file pone.0239197.s001.pdf]

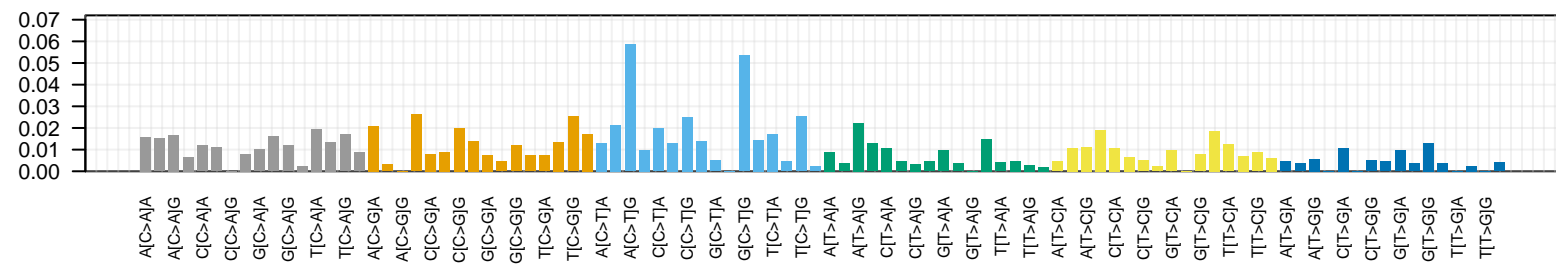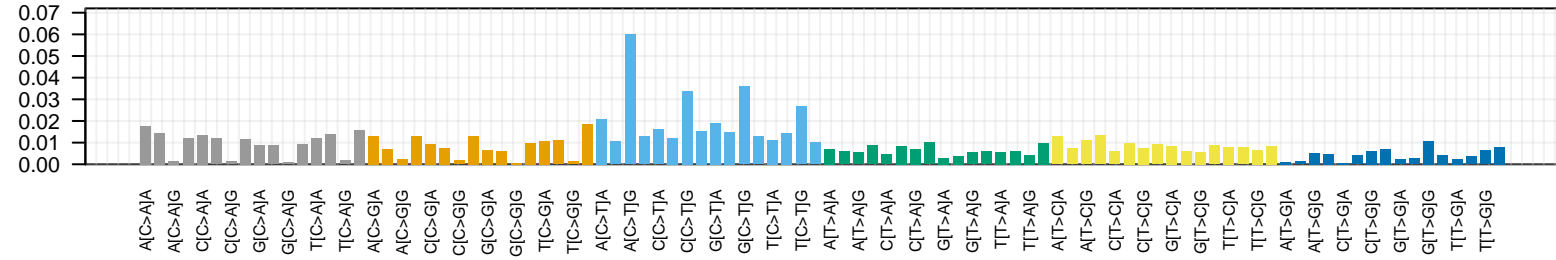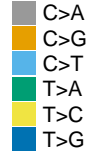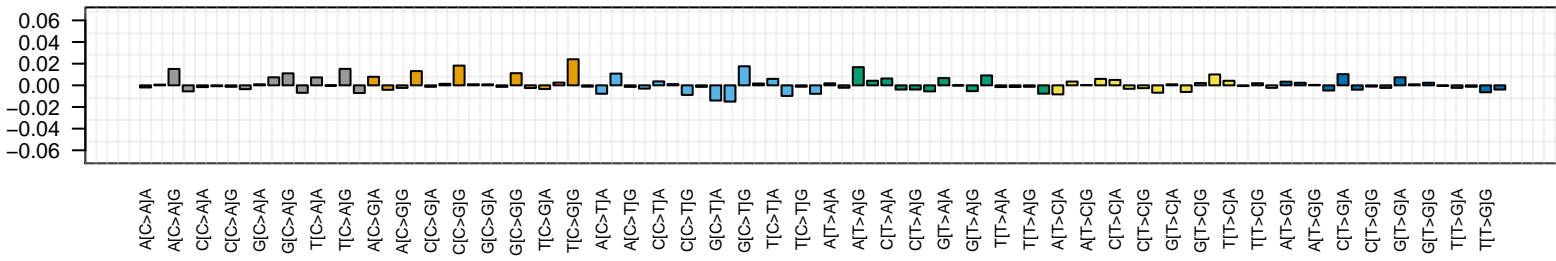

Supplement: S2 Fig — This tumor had a large proportion of C>T mutations, which are representative of Signature 3. (PDF) [file pone.0239197.s002.pdf]

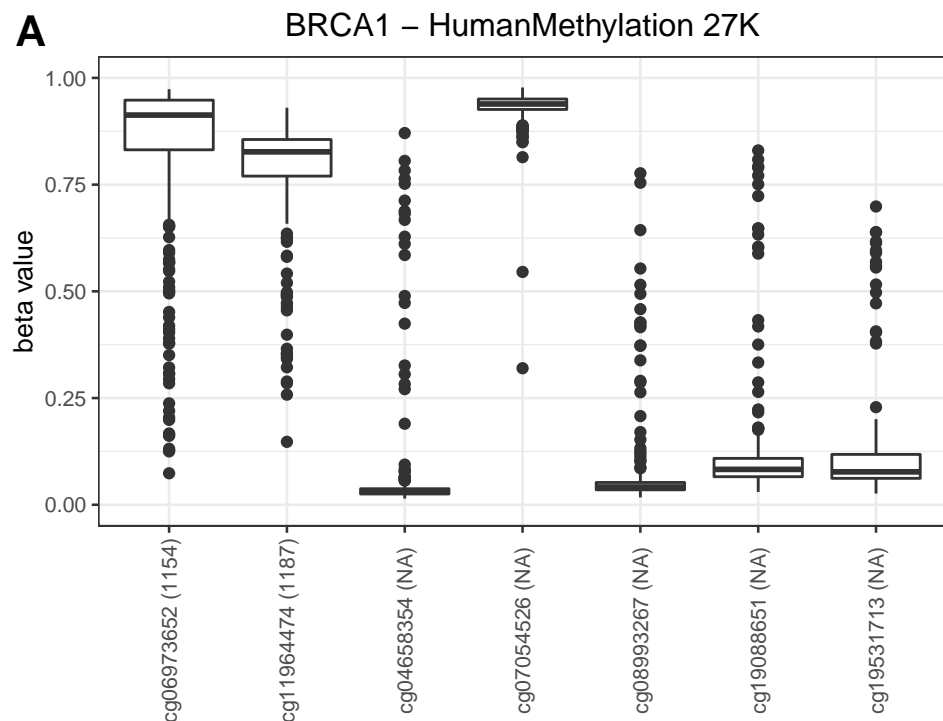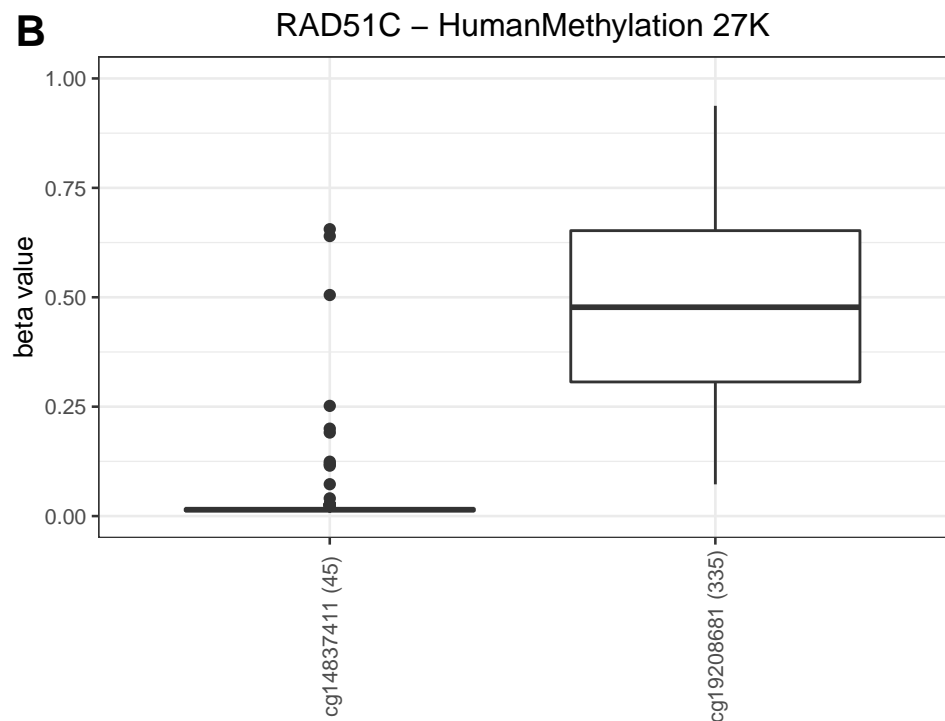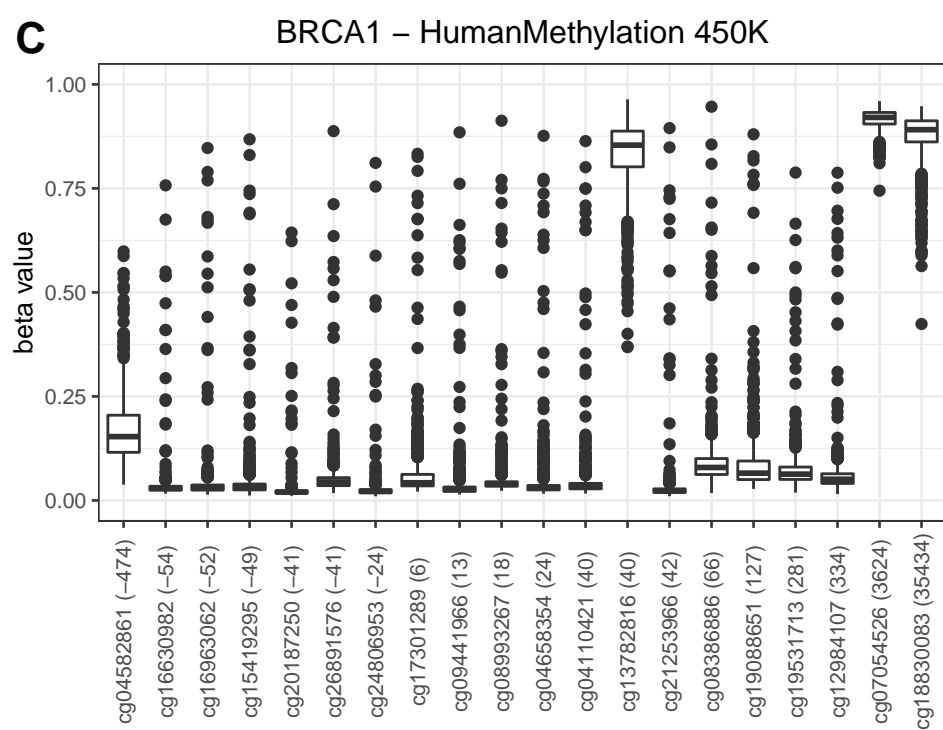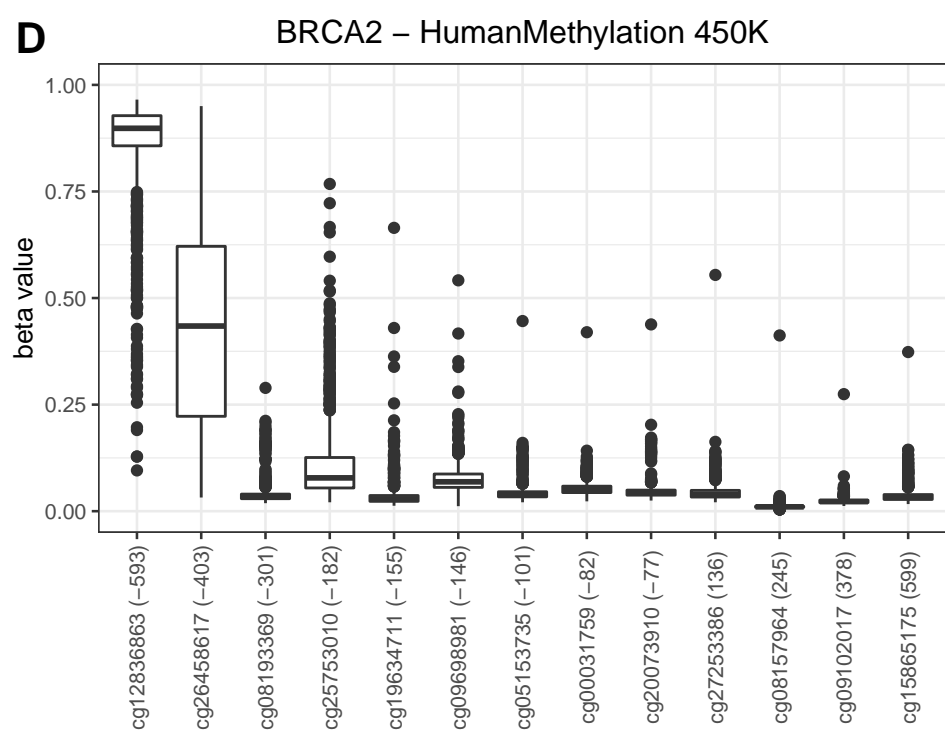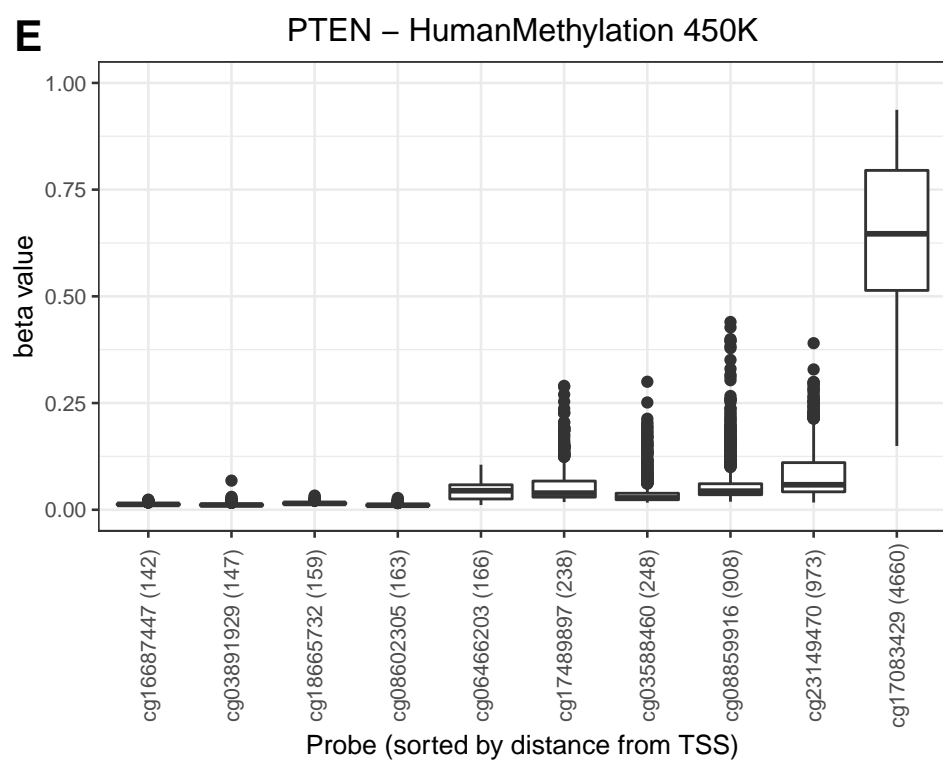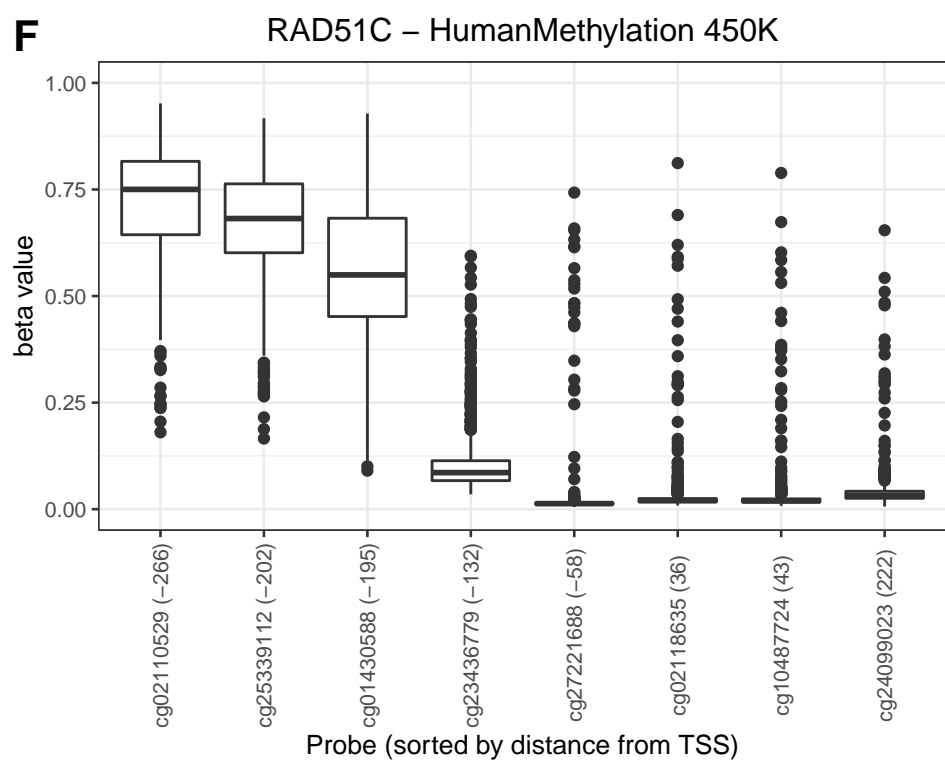

Supplement: S3 Fig — We extracted probe-level methylation (beta) values for all available breast-cancer samples in TCGA and plotted them relative to the transcription start site of each gene. These graphs illustrate beta values for four genes (BRCA1, BRCA2, PTEN, and RAD51C) and two microarray platforms (Illumina HumanMethylation 27K and 450K). Values in parenthesis indicate distance from the transcription start site (TSS). TSS distances marked as “NA” were unavailable. The 27K arrays have fewer probes per gene. In general, probes near the TSS exhibited relatively low methylation levels for these genes, whereas probes further from the TSS were more highly methylated. These observations are consistent with the assumption that most genes would be “on” by default. Some exceptions to this pattern are apparent (for example, cg13782816 on panel C); these exceptions may be caused by mismapped probes, cross hybridization, or misannotations. We calculated gene-level values as the median across all probes that were within 300 nucleotides of the TSS. (PDF) [file pone.0239197.s003.pdf]

## BRCA1 (n=36)

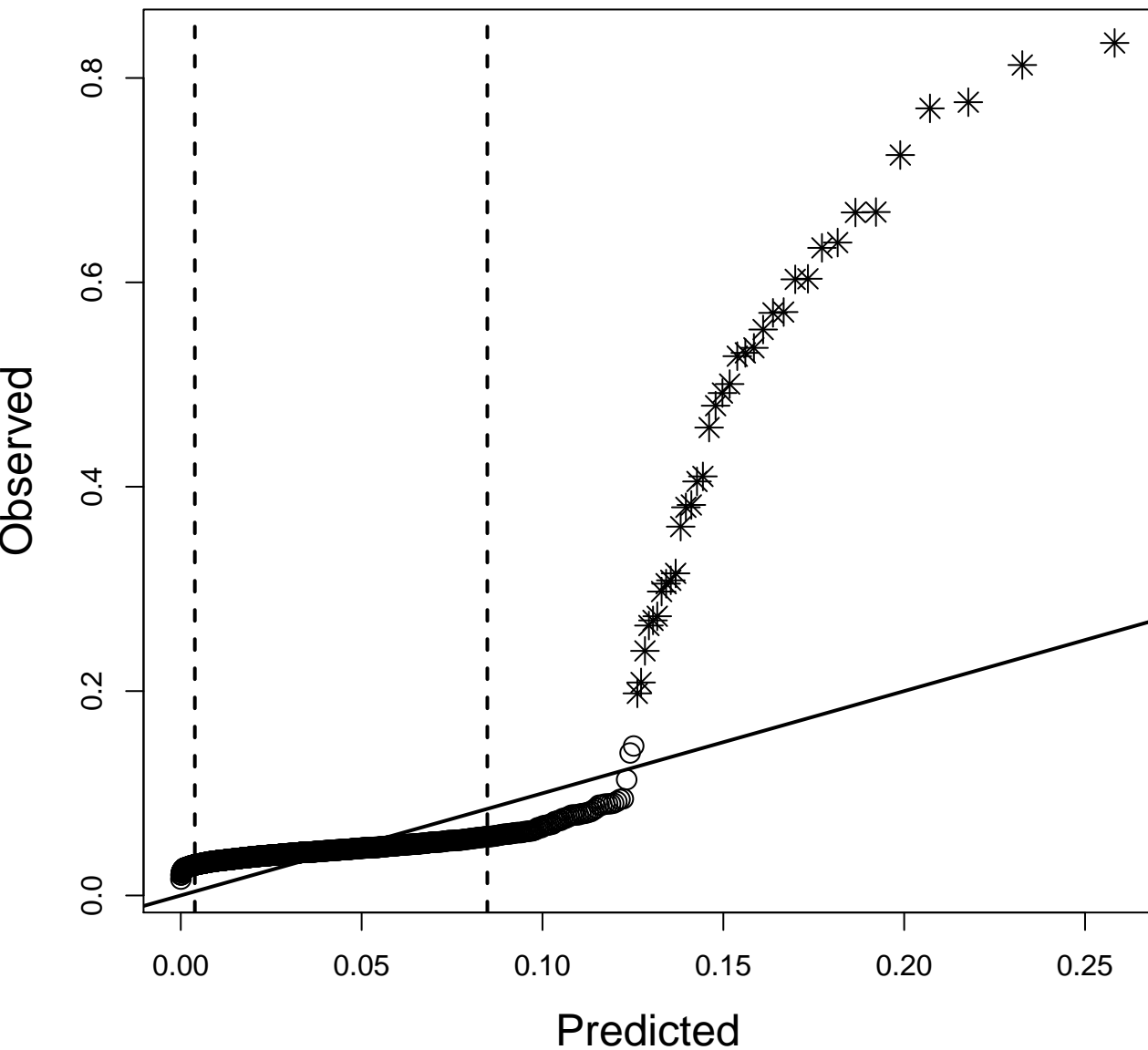

Supplement: S4 Fig — We used an outlier-detection methodology to estimate which tumors were hypermethylated for a given gene. This scatter plot illustrates the model fit for BRCA1. Asterisks represent tumors considered to be outliers. (PDF) [file pone.0239197.s004.pdf]

# BRCA2 (n=2)

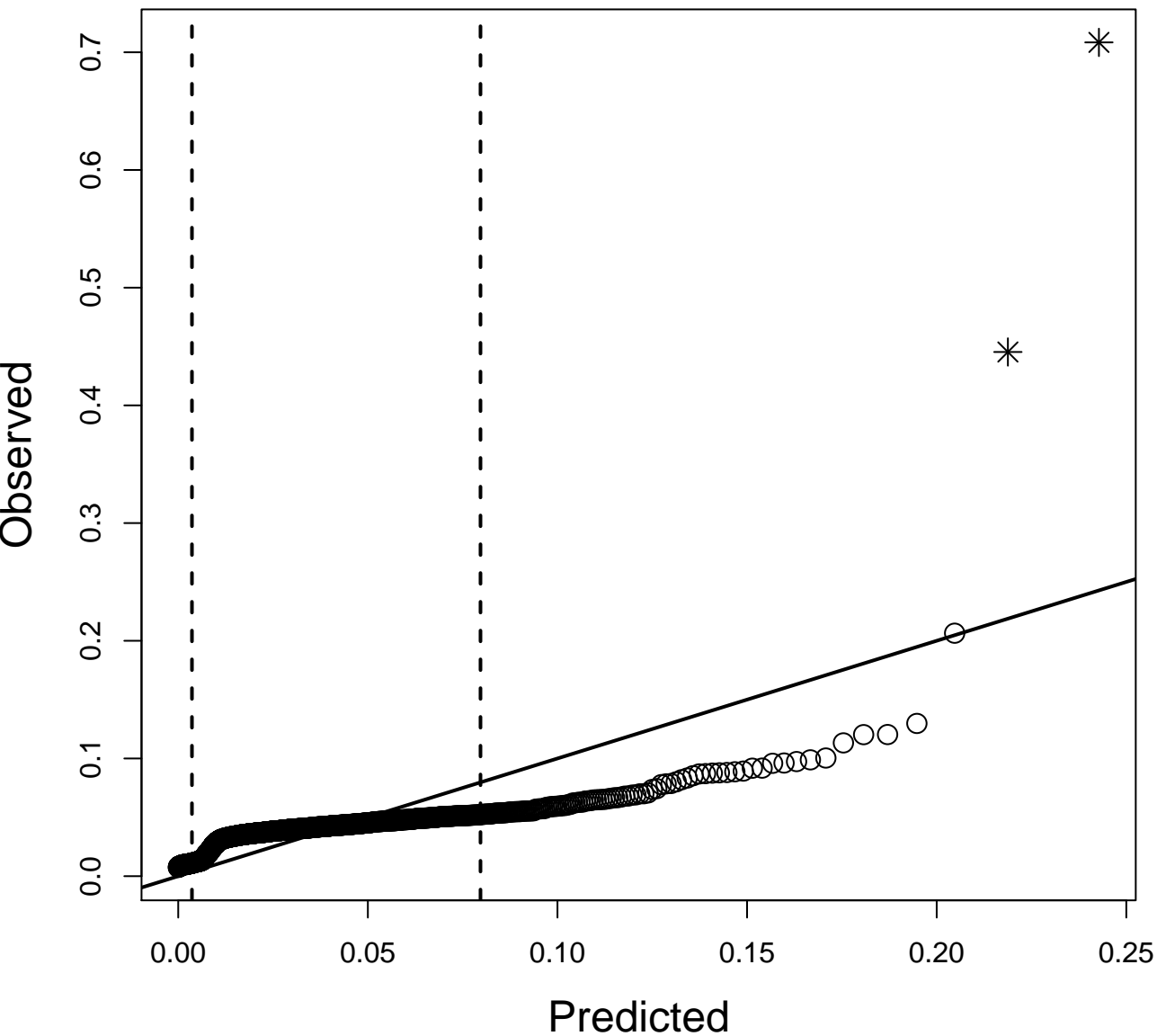

Supplement: S5 Fig — We used an outlier-detection methodology to estimate which tumors were hypermethylated for a given gene. This scatter plot illustrates the model fit for BRCA2. Asterisks represent tumors considered to be outliers. (PDF) [file pone.0239197.s005.pdf]

# PTEN (n=2)

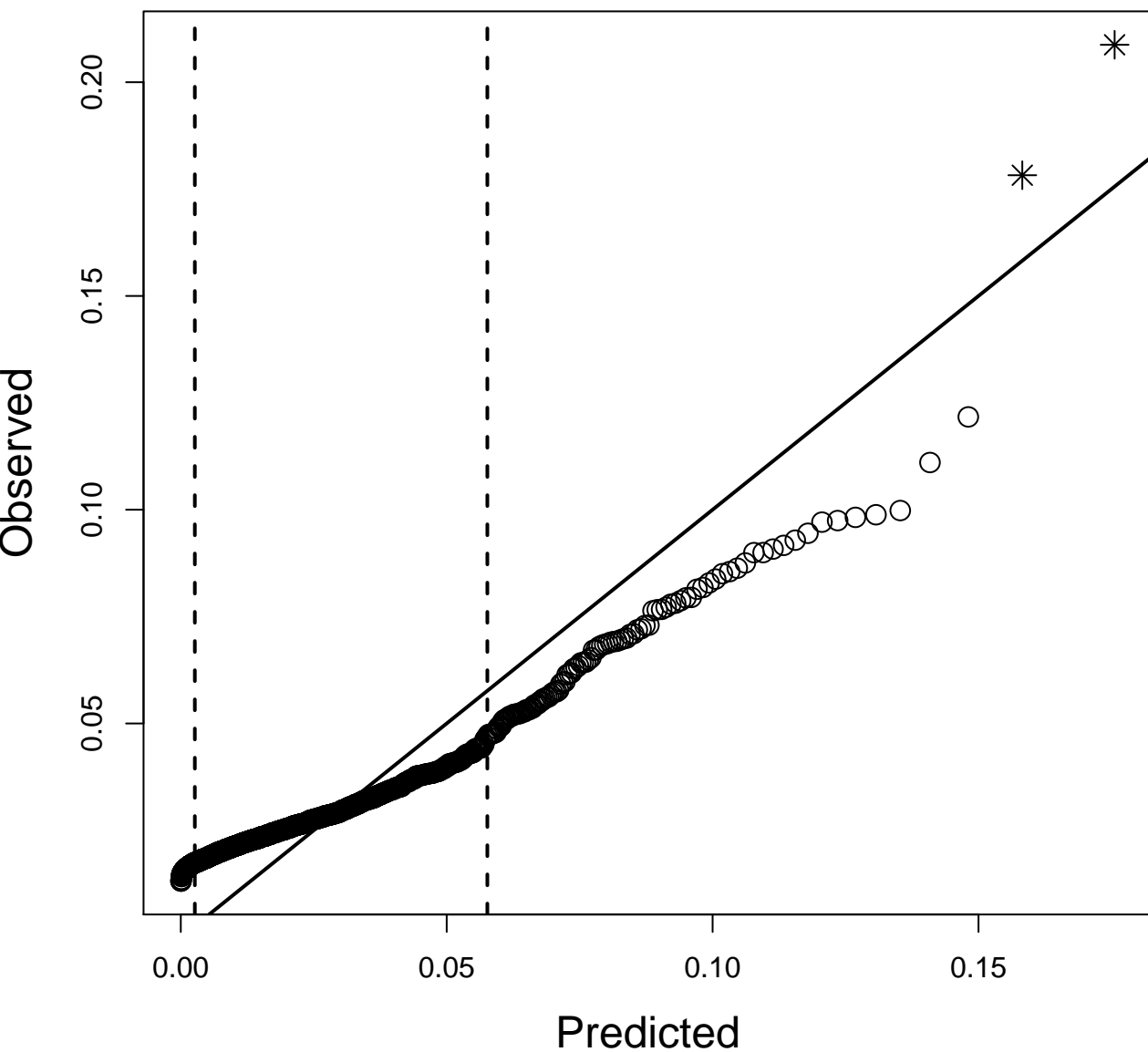

Supplement: S6 Fig — We used an outlier-detection methodology to estimate which tumors were hypermethylated for a given gene. This scatter plot illustrates the model fit for PTEN. Asterisks represent tumors considered to be outliers. (PDF) [file pone.0239197.s006.pdf]

# RAD51C (n=35)

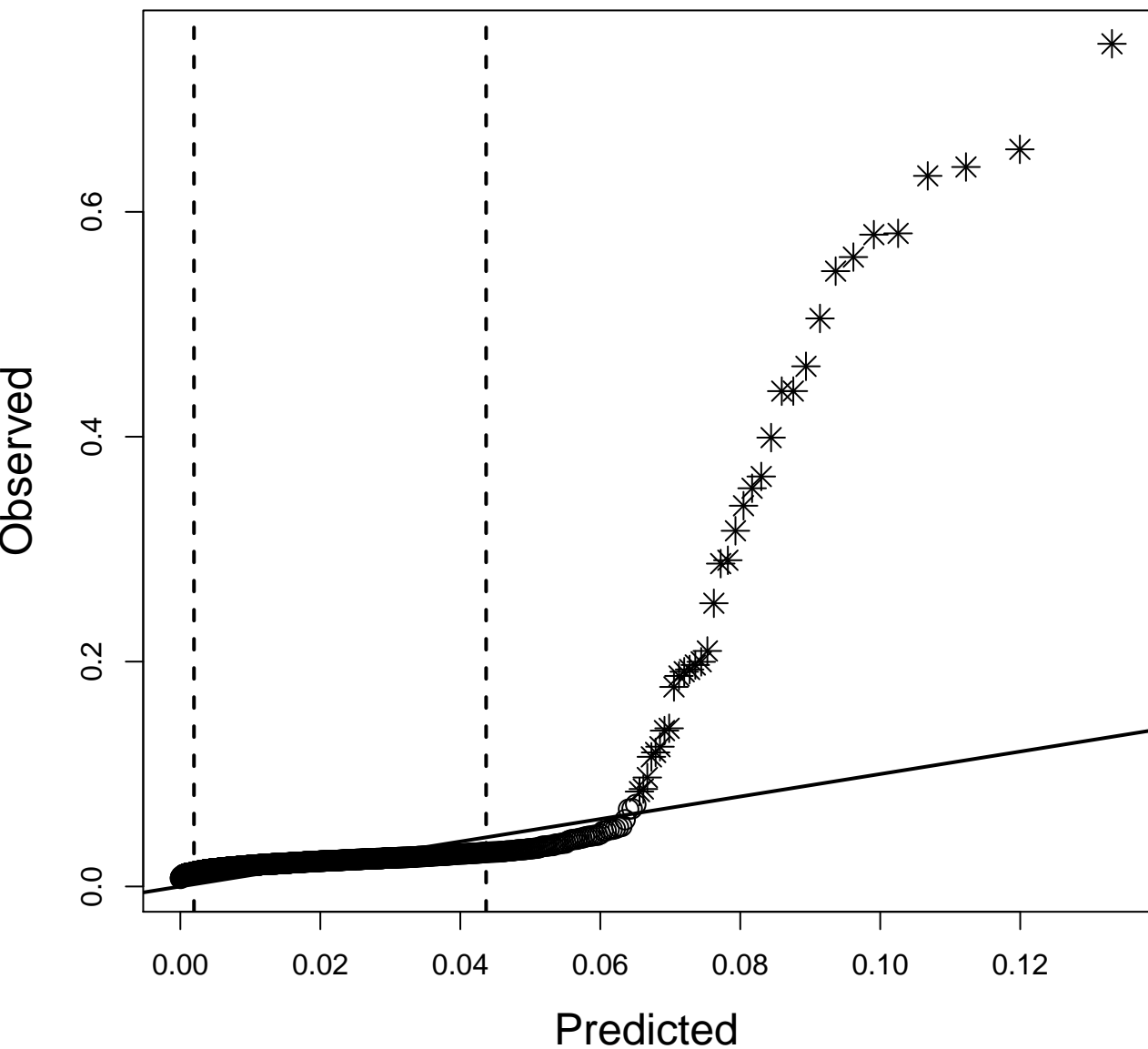

Supplement: S7 Fig — We used an outlier-detection methodology to estimate which tumors were hypermethylated for a given gene. This scatter plot illustrates the model fit for RAD51C. Asterisks represent tumors considered to be outliers. (PDF) [file pone.0239197.s007.pdf]

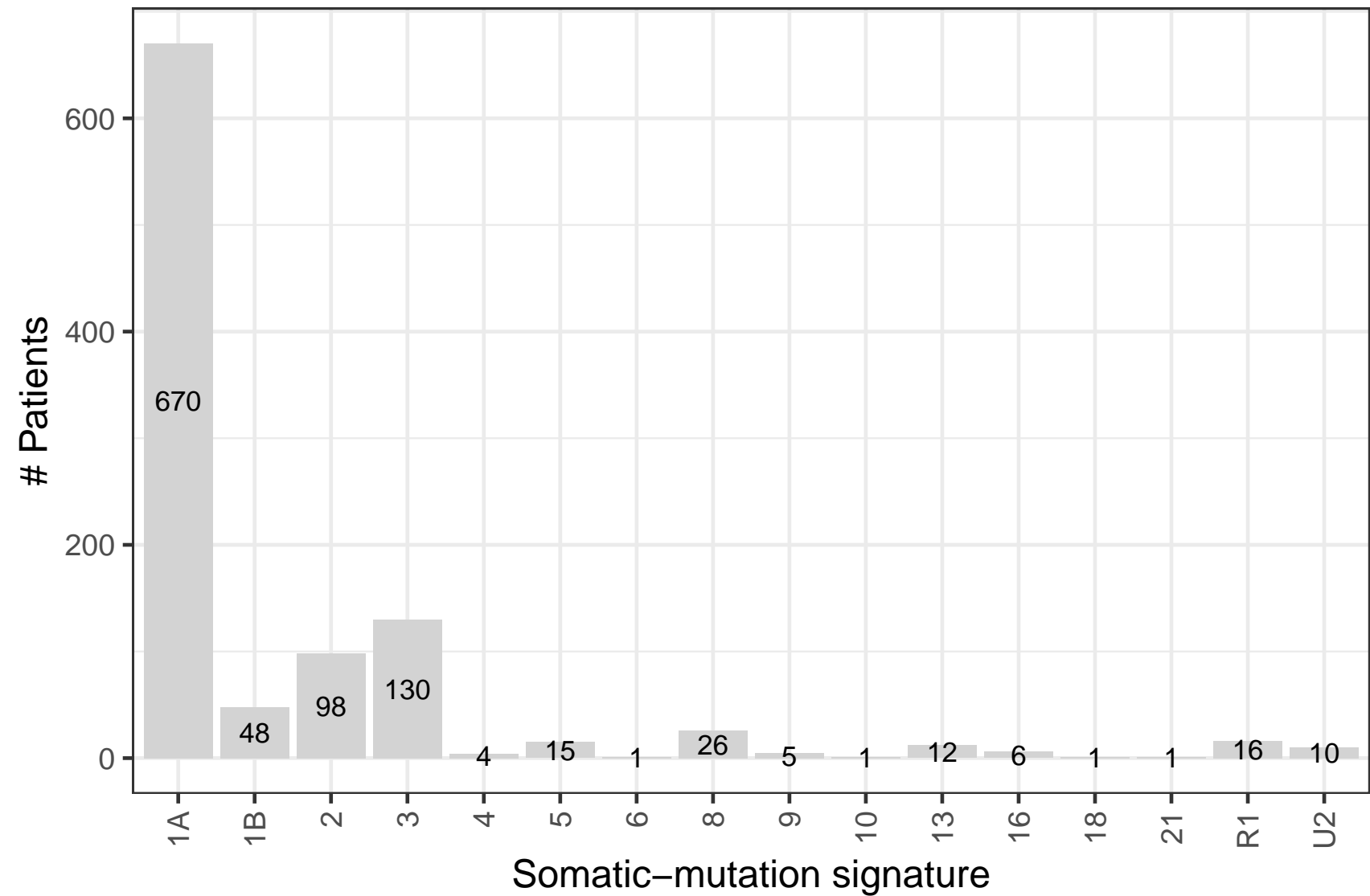

Supplement: S8 Fig — Each TCGA breast-cancer patient was assigned to a primary somatic-mutation signature based on exome-wide mutational patterns. This plot illustrates the frequency of each somatic-mutation signature across all the patients. (PDF) [file pone.0239197.s008.pdf]

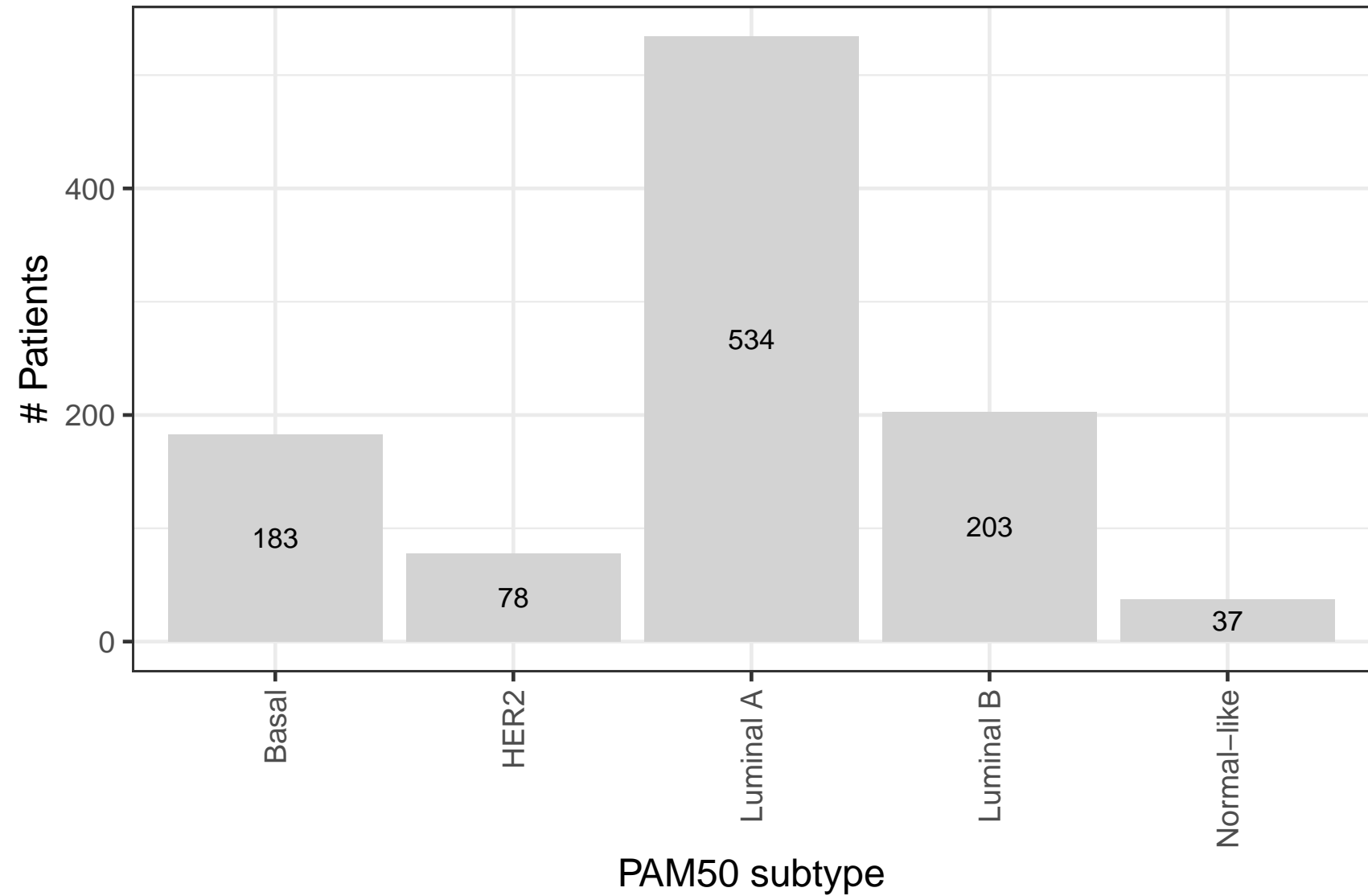

Supplement: S9 Fig — Each TCGA breast-cancer patient was assigned to a primary PAM50 subtype based on tumor gene-expression levels. This plot illustrates the frequency of each PAM50 subtype across all the patients. (PDF) [file pone.0239197.s009.pdf]

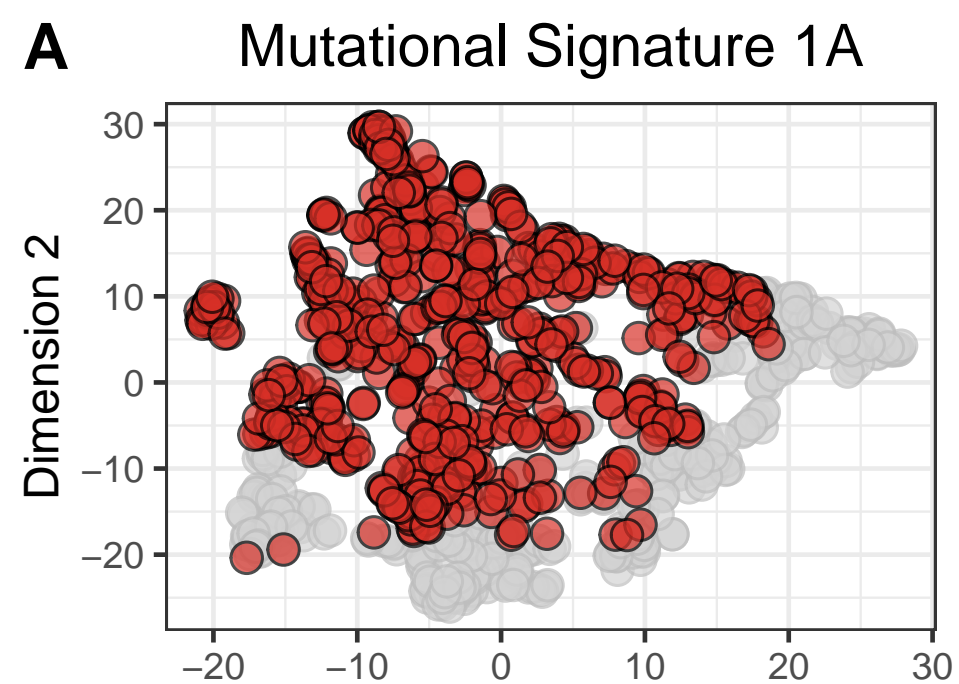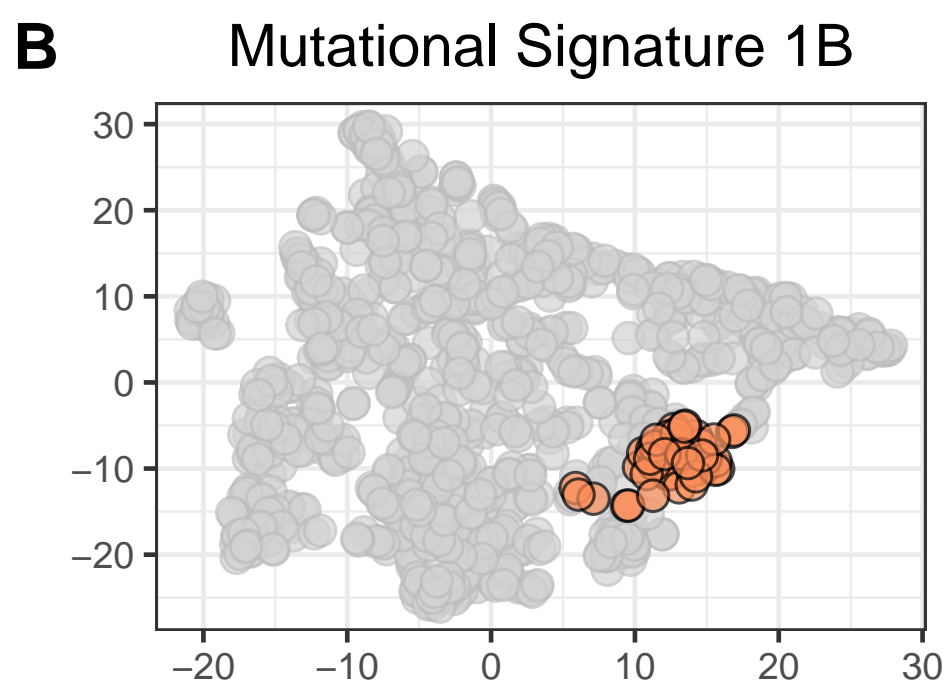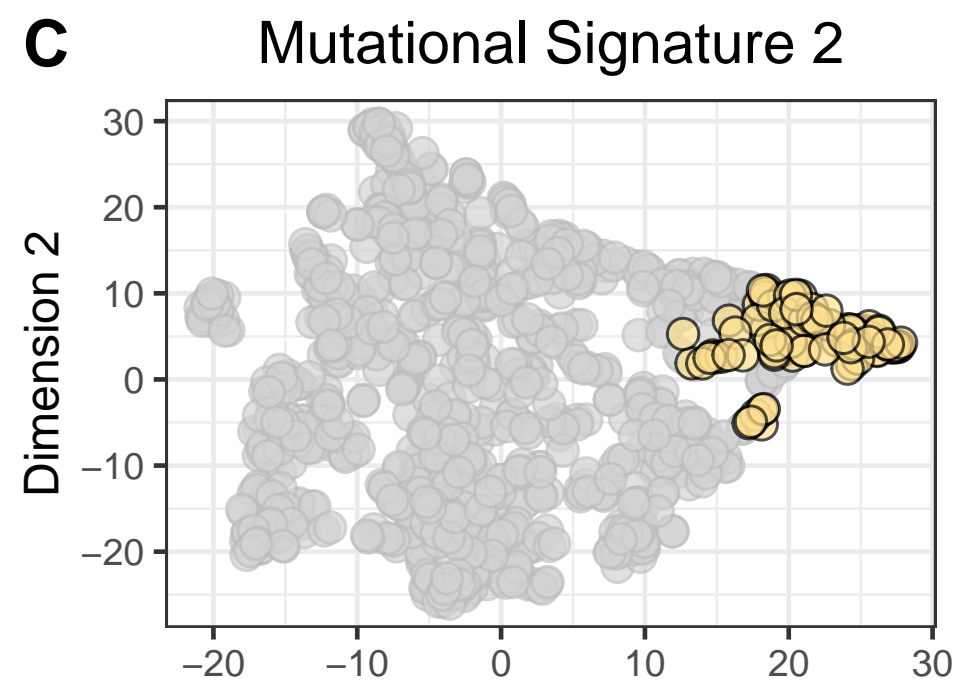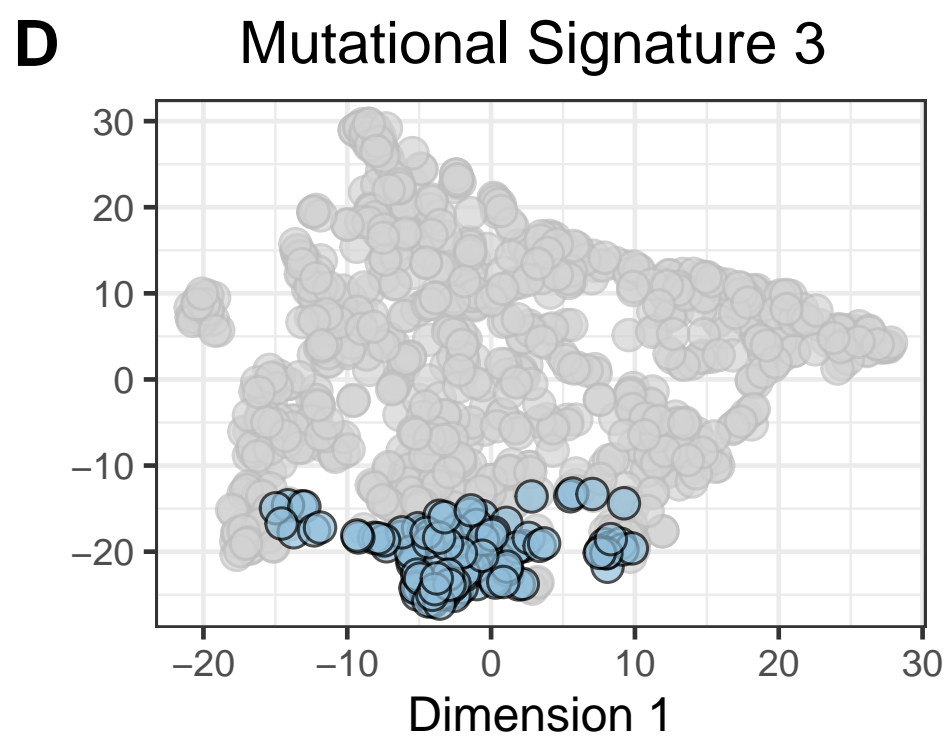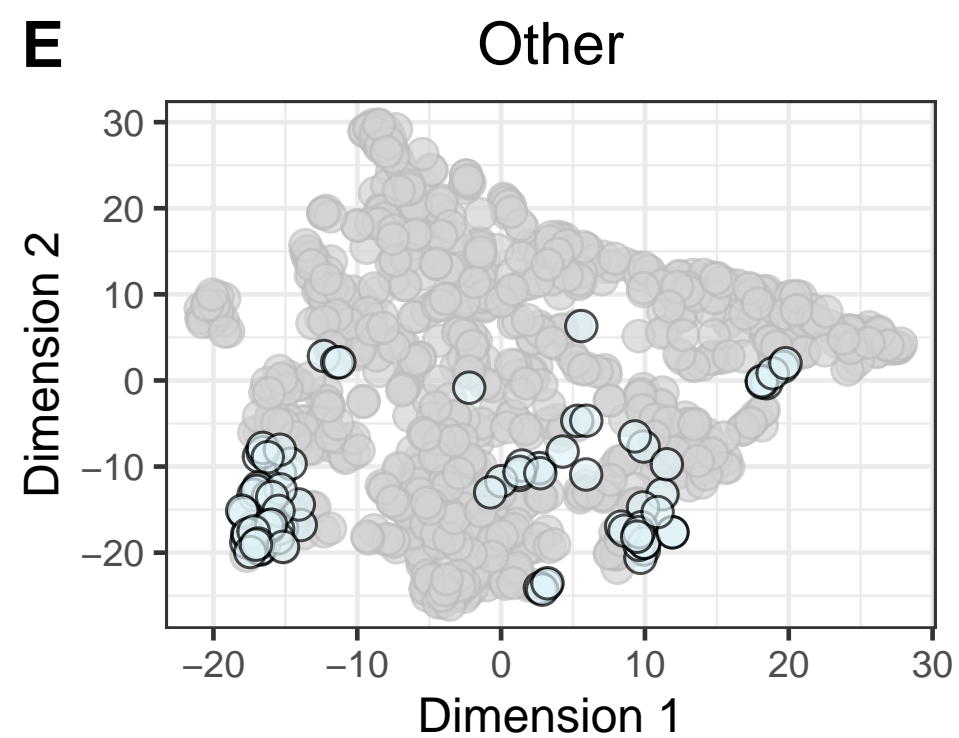

Supplement: S10 Fig — We summarized each tumor based on their somatic-mutation signatures, which represent overall mutational patterns in a trinucleotide context. We used the t-distributed Stochastic Neighbor Embedding (t-SNE) method to reduce the data to two dimensions. Each point represents a single tumor, overlaid with colors that represent the tumor’s primary somatic-mutation signature. Mutational Signature 1A (A) was the most prevalent; these tumors were widely dispersed across the signature landscape. Signatures 1B (B), 2 (C), and 3 (D) were relatively small and formed cohesive clusters. The remaining 23 clusters were rare individually and were dispersed broadly. (PDF) [file pone.0239197.s010.pdf]

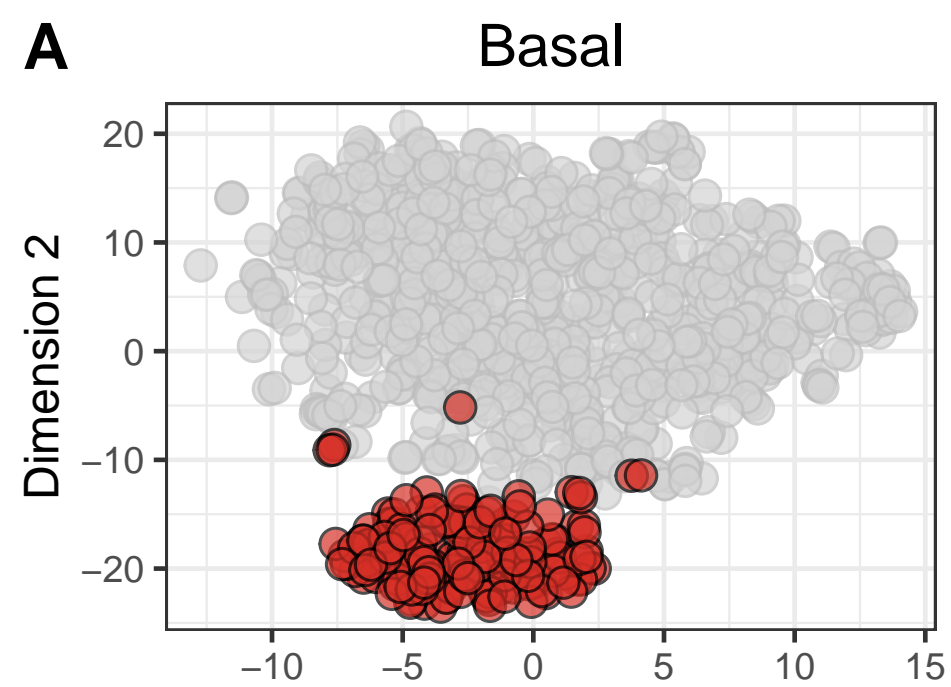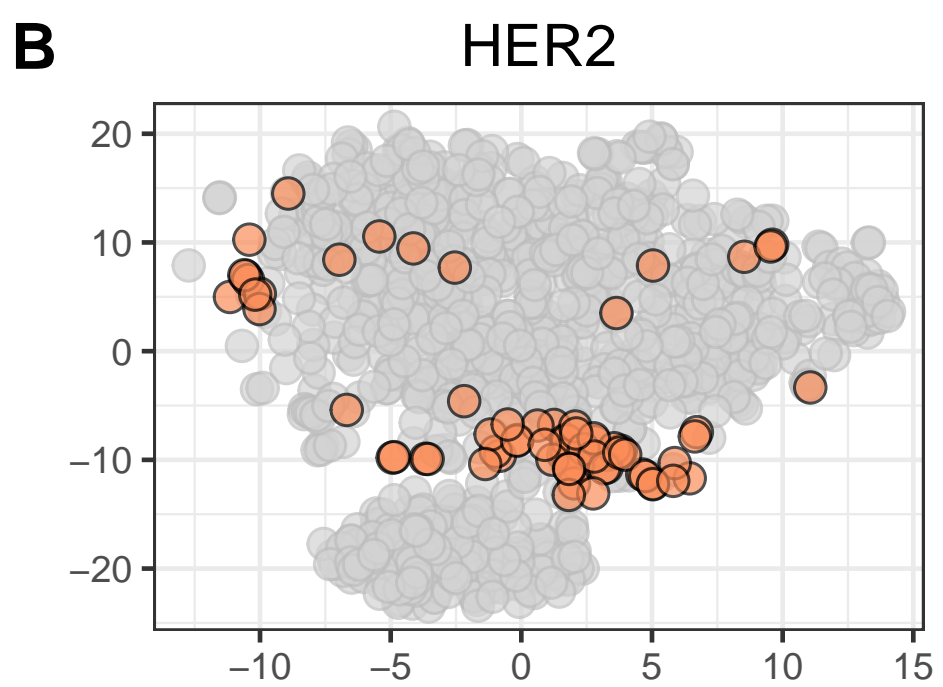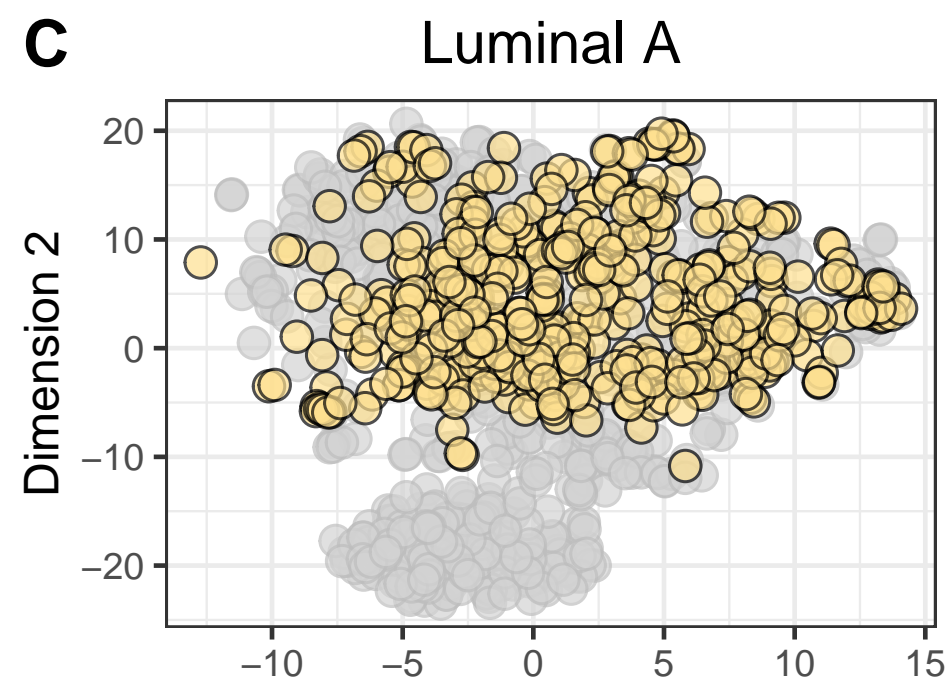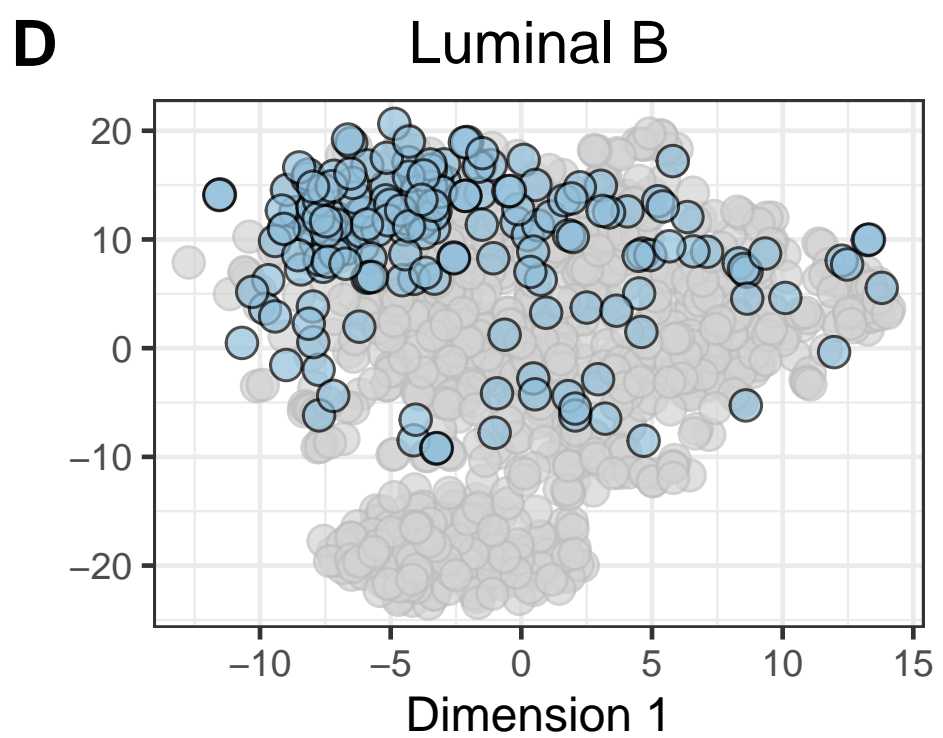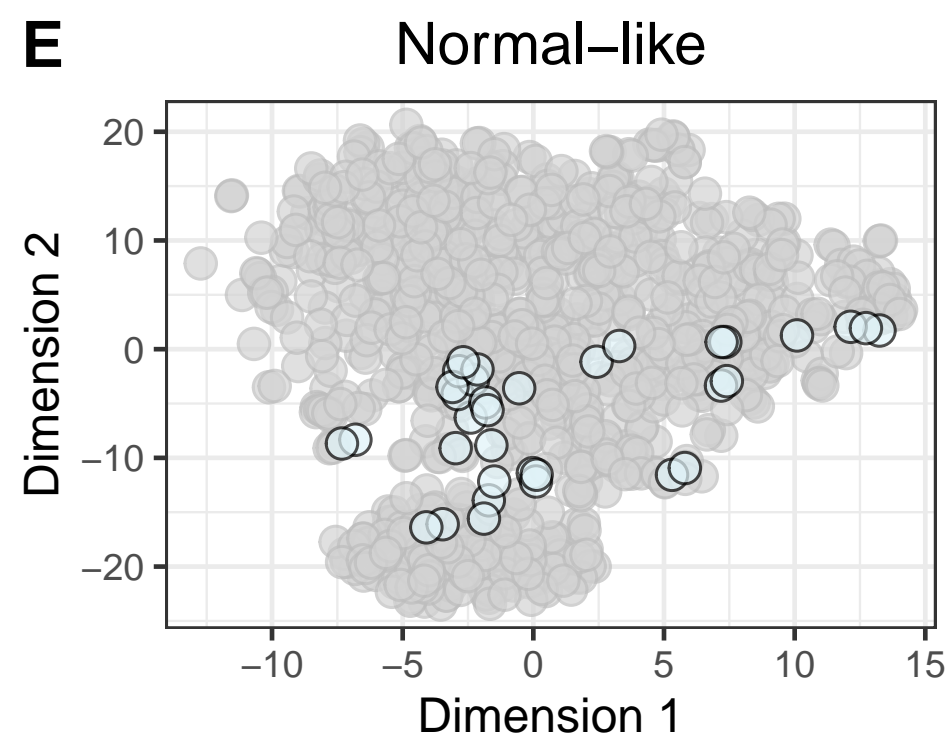

Supplement: S11 Fig — We used the t-distributed Stochastic Neighbor Embedding (t-SNE) method to reduce the gene-expression profiles to two dimensions. Each point represents a single tumor, overlaid with colors that represent the tumor’s primary PAM50 subtype. Generally, the PAM50 subtypes clustered cohesively, but there were exceptions. For example, some Basal-like tumors (A) exhbited expression patterns that differed considerably from the remaining Basal-like tumors. The normal-like tumors (E) showed the most variability in expression. This graph represents patients for whom we could identify a PAM50 subtype. (PDF) [file pone.0239197.s011.pdf]

Intersection Size

20  
15  
10  
5  
0

3

19

BRCA1 germline mutation

BRCA1 loss of heterozygosity

20 15 10 5 0

Set Size

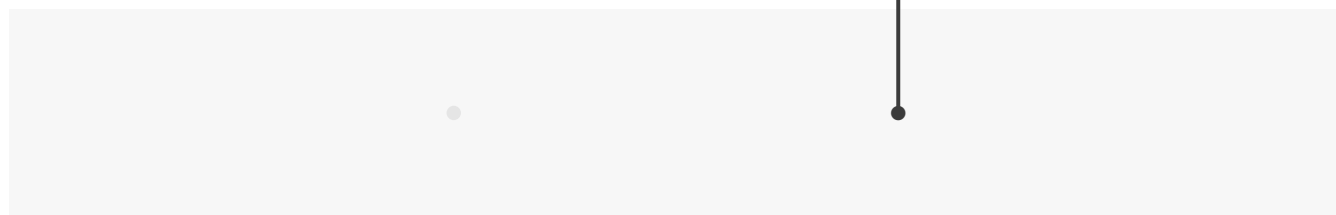

Supplement: S12 Fig — A total of 22 patients carried a germline mutation in BRCA1. We detected loss-of-heterozygosity events in tumors for all but 3 of these patients. Data are only shown for patients for whom we had both types of data. (PDF) [file pone.0239197.s012.pdf]

Intersection Size

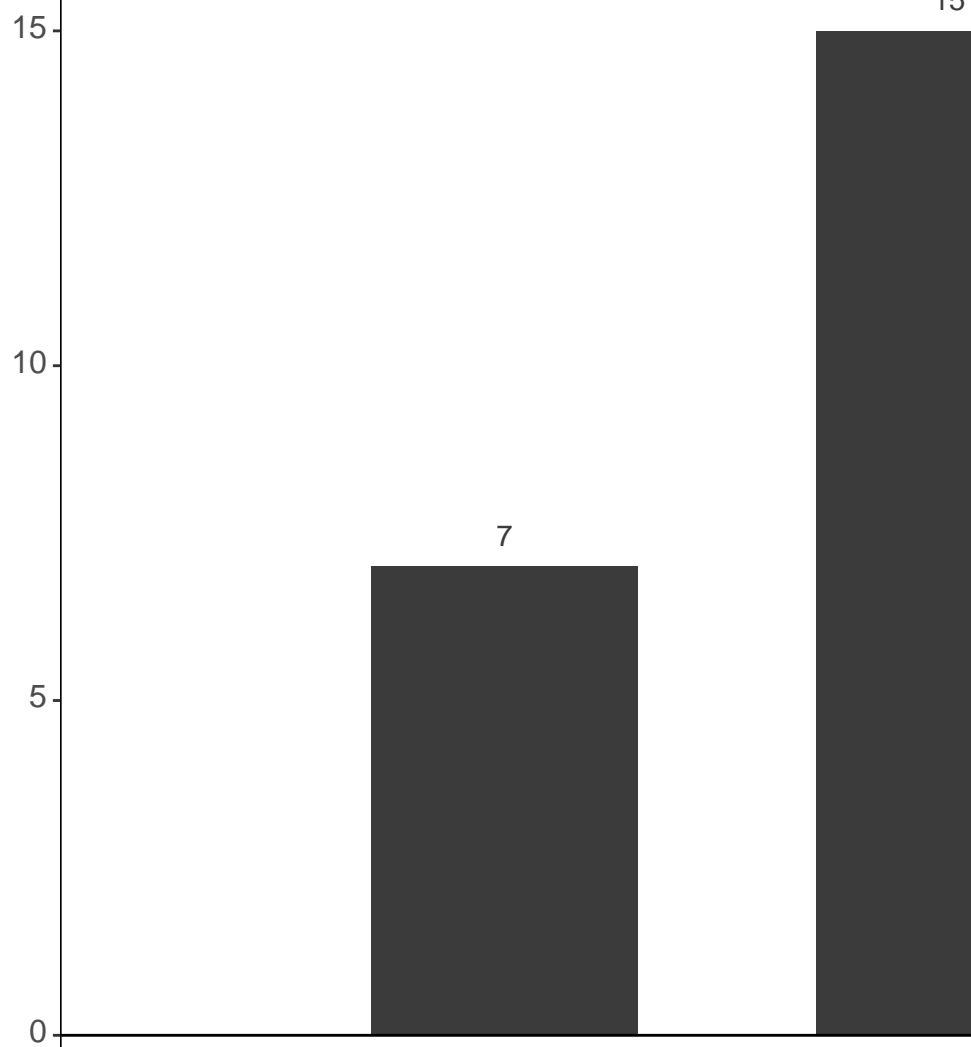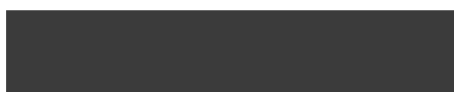

BRCA2 germline mutation

BRCA2 loss of heterozygosity

20 15 10 5 0

Set Size

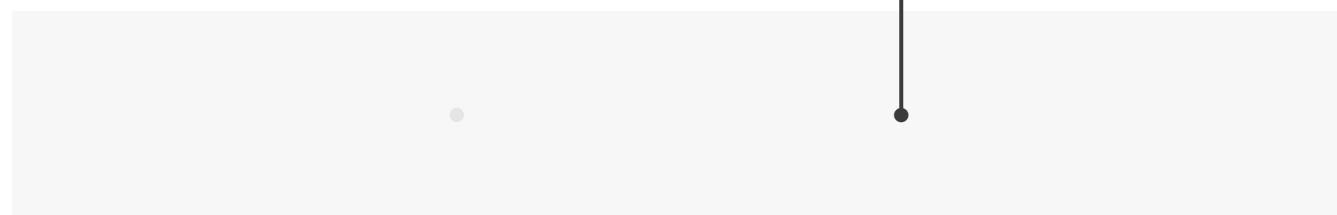

Supplement: S13 Fig — A total of 22 patients carried a germline mutation in BRCA2. We detected loss-of-heterozygosity events in tumors from all but 7 of these patients. Data are only shown for patients for whom we had both types of data. (PDF) [file pone.0239197.s013.pdf]

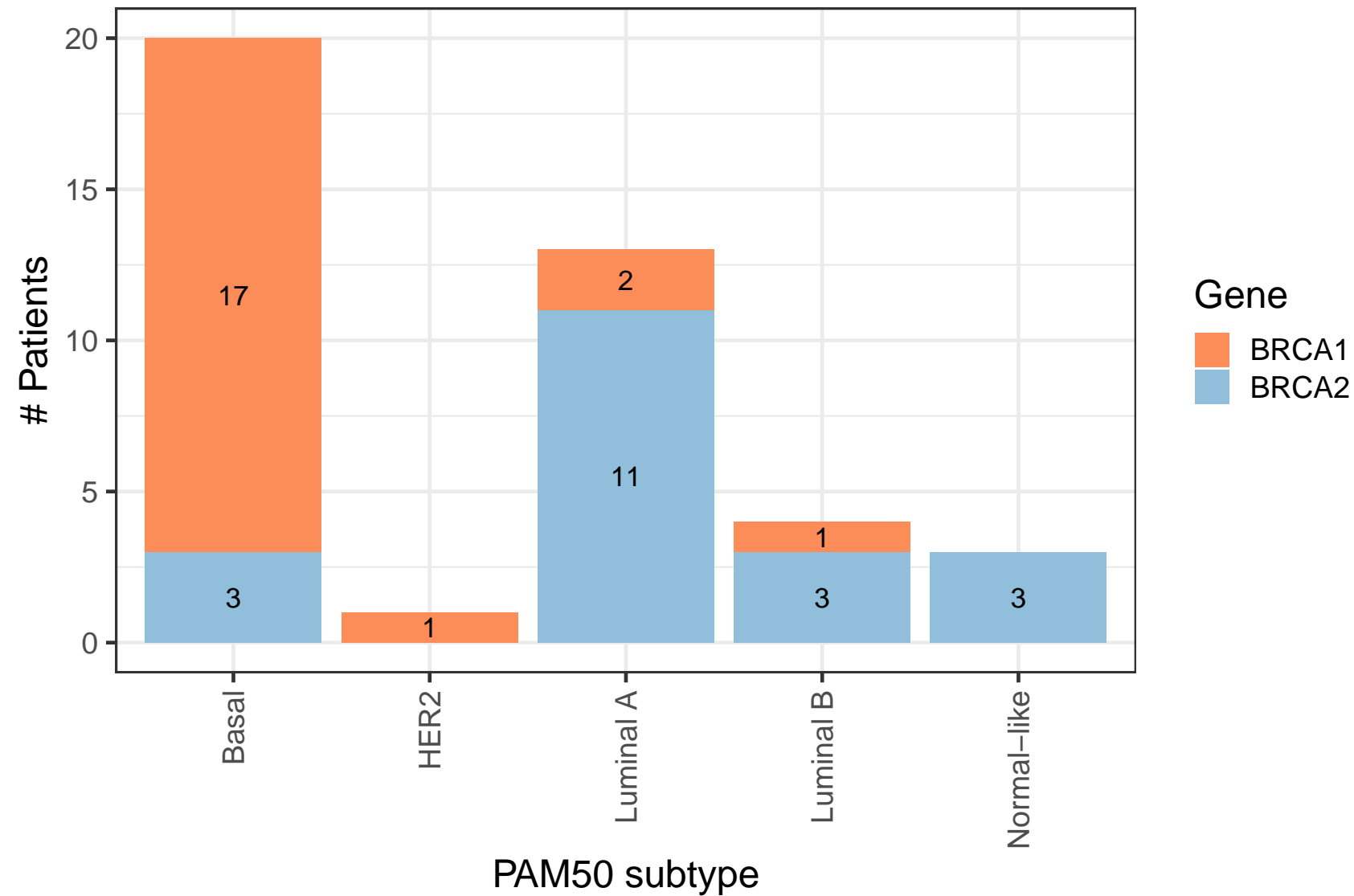

Supplement: S15 Fig — Gene-expression subtypes were unavailable for some patients; One BRCA1 carrier is not represented in this figure; we could not assign a gene-expression subtype to this individual due to missing data. (PDF) [file pone.0239197.s015.pdf]

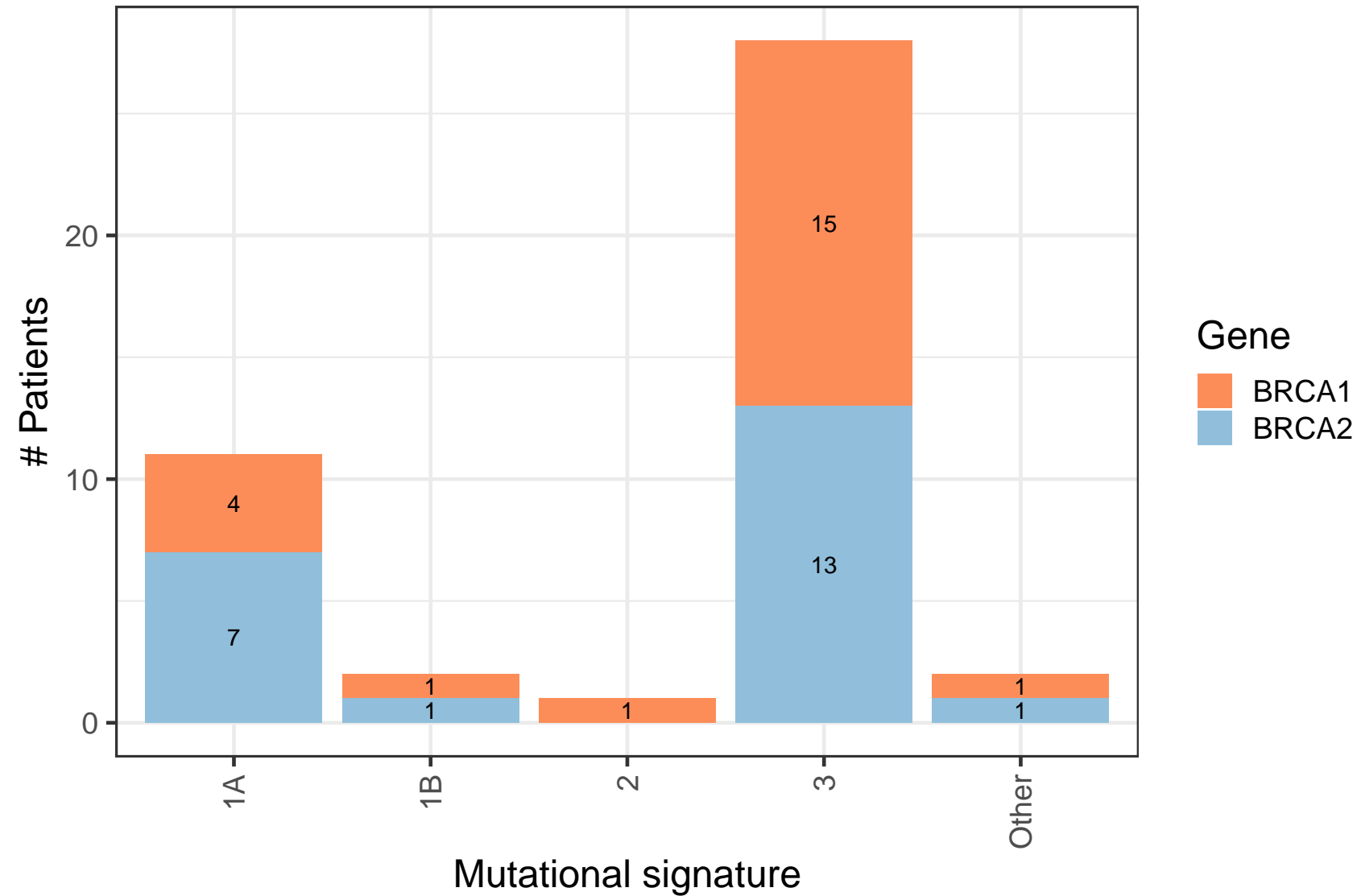

Supplement: S16 Fig — This graph represents patients for whom we had both germline- and somatic-mutation data. (PDF) [file pone.0239197.s016.pdf]

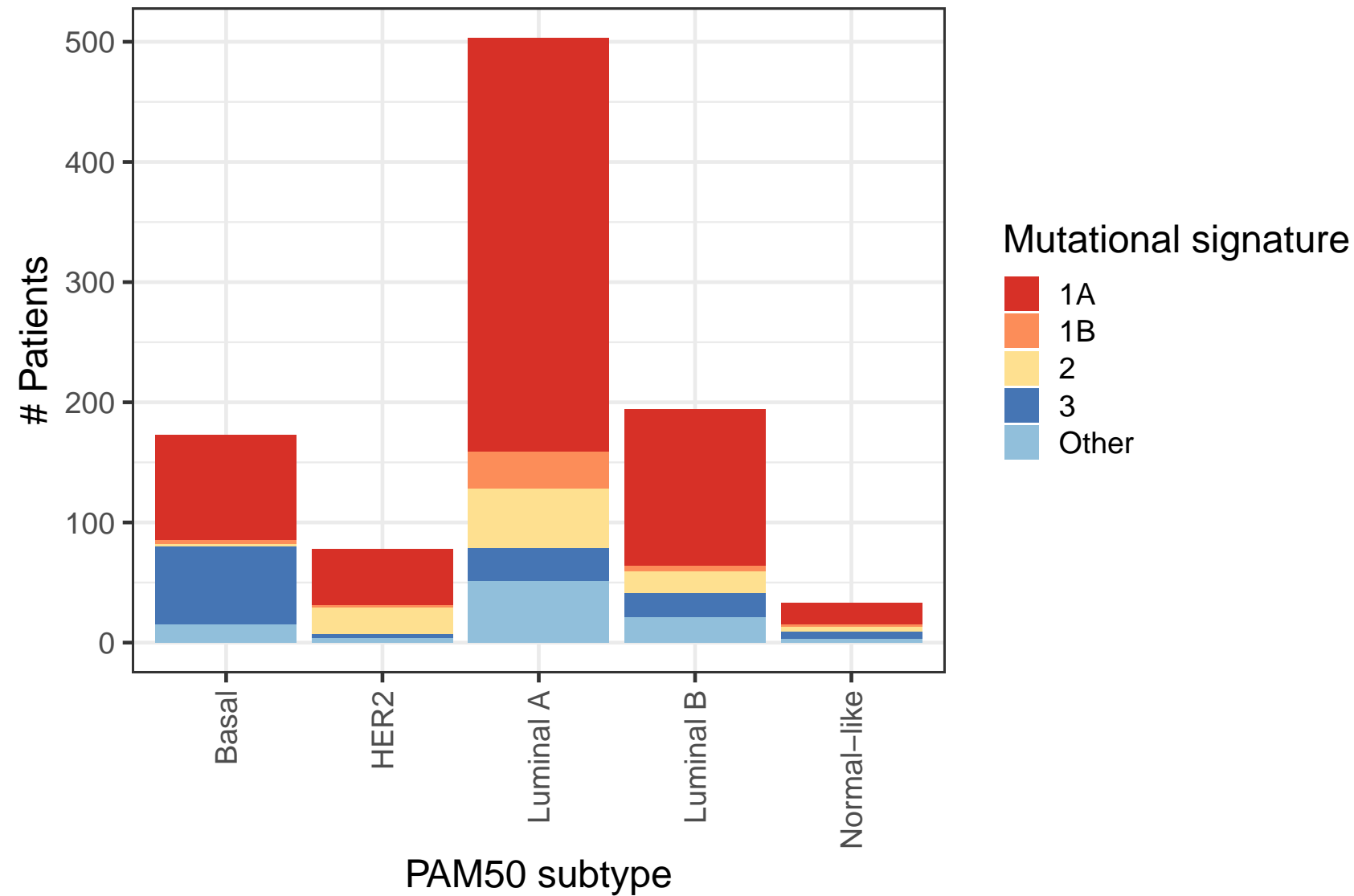

Supplement: S17 Fig — This graph represents patients for whom we could evaluate the status of both PAM50 subtype and somatic-mutation signatures. (PDF) [file pone.0239197.s017.pdf]

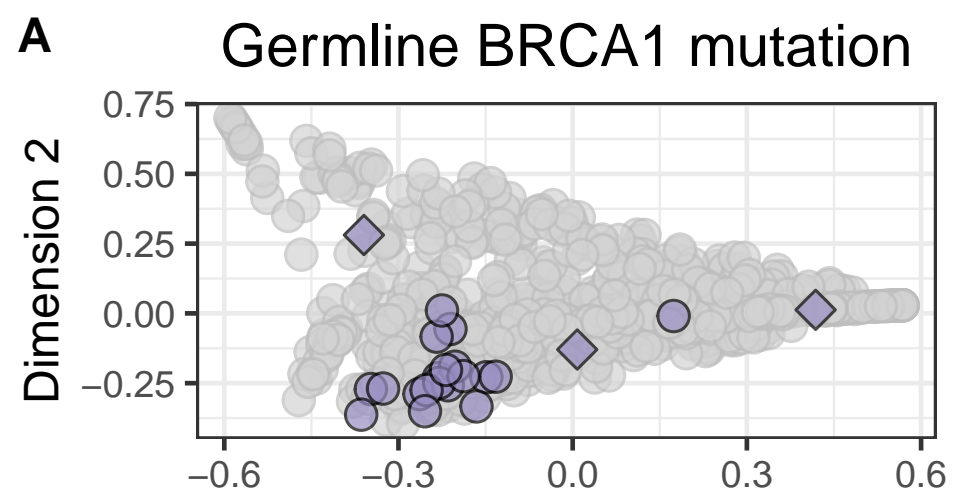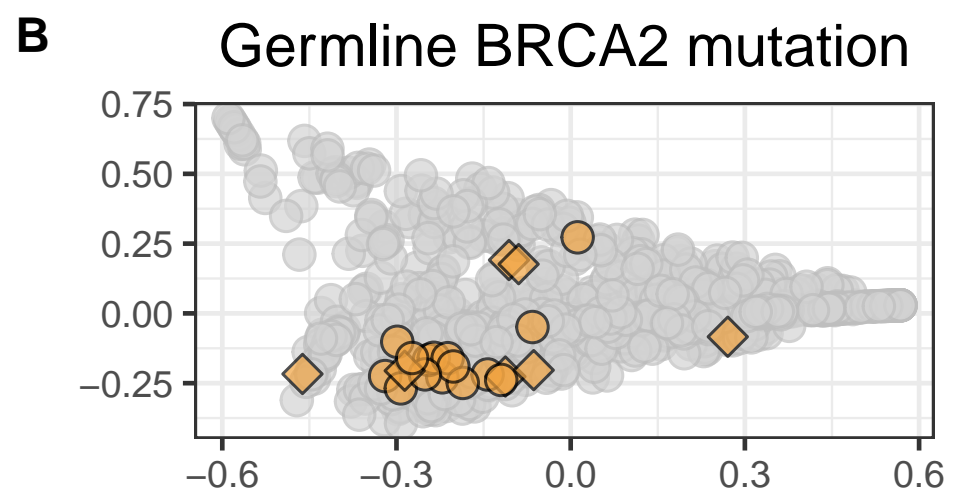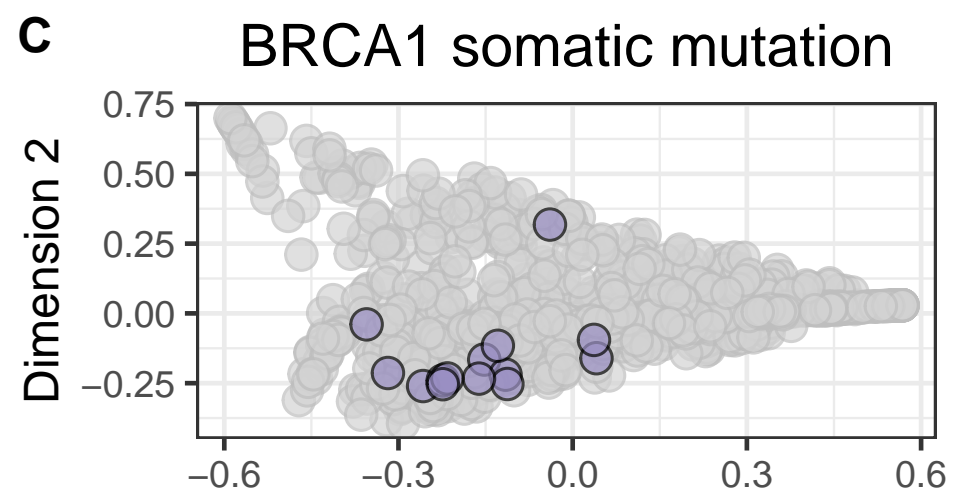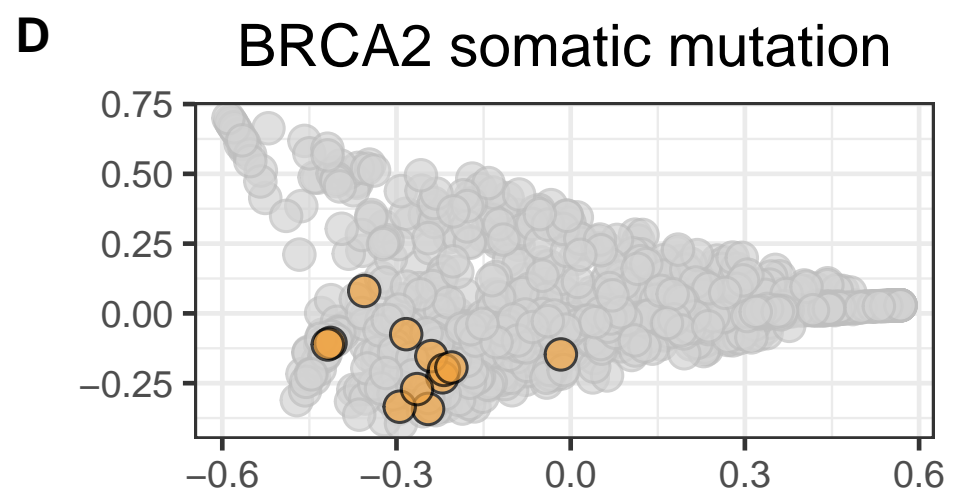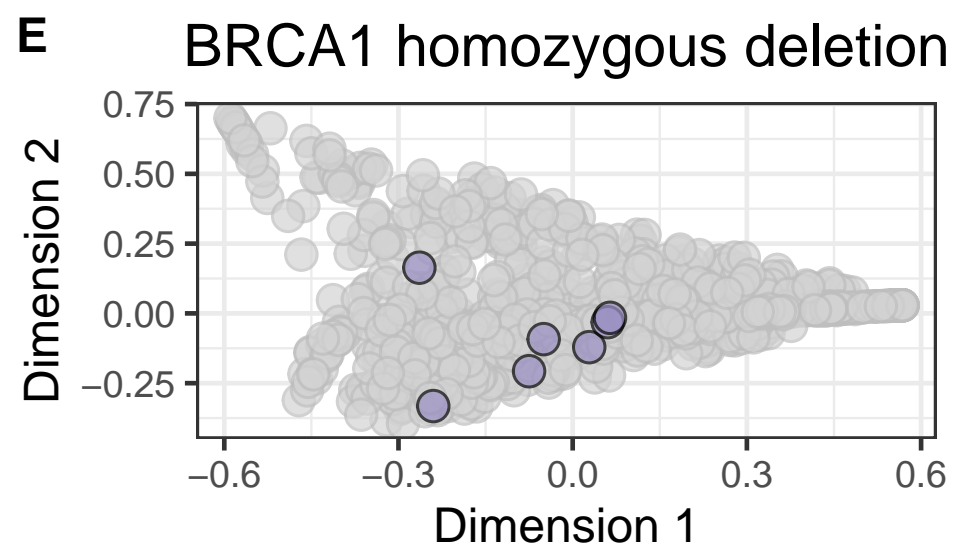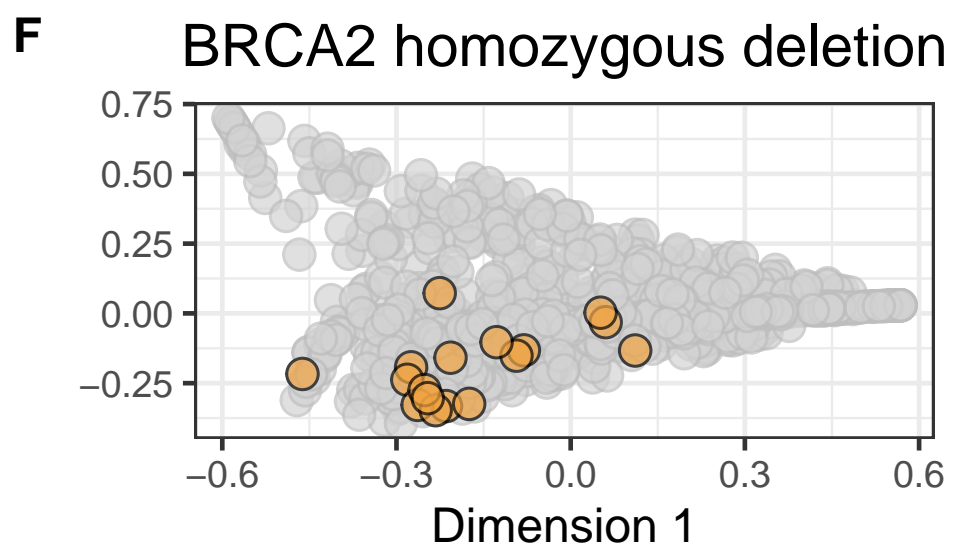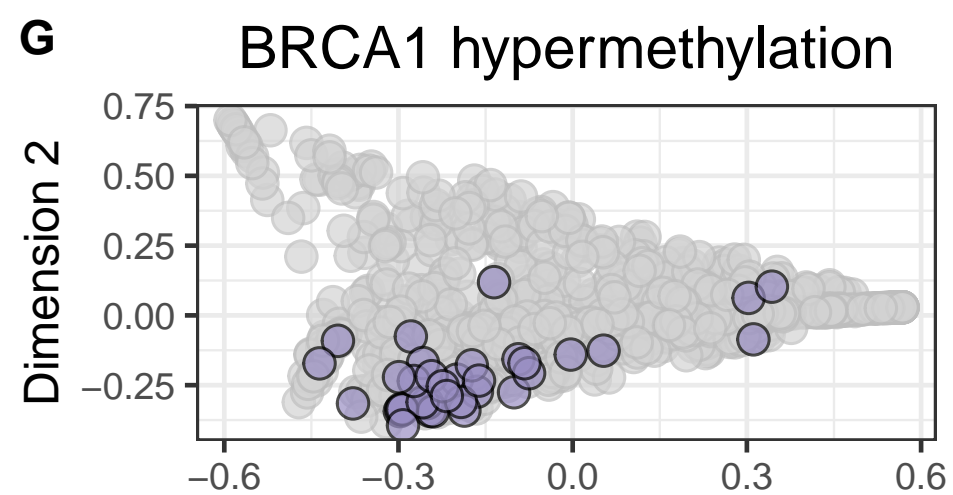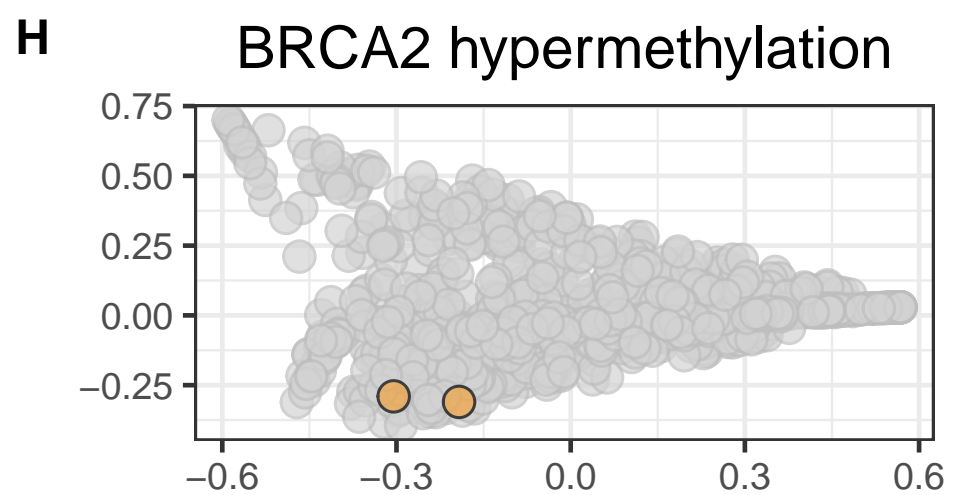

Supplement: S18 Fig — Using the same two-dimensional representation of mutational signatures shown in Fig 1, this plot indicates which patients had germline mutations (A, B), somatic mutations (C, D), homozygous deletions (E, F), or hypermethylation events (G, H) in BRCA1 and BRCA2, respectively. Largely, these tumors had similar somatic-mutation signatures. Diamond shapes indicate patients for whom no loss-of-heterozygosity was observed. Data are shown for all patients, even those for whom we did not have all types of data. (PDF) [file pone.0239197.s018.pdf]

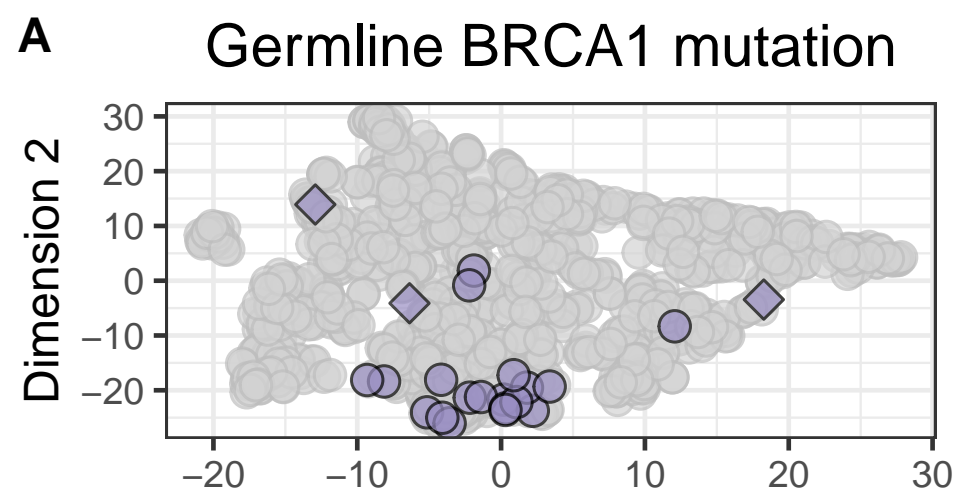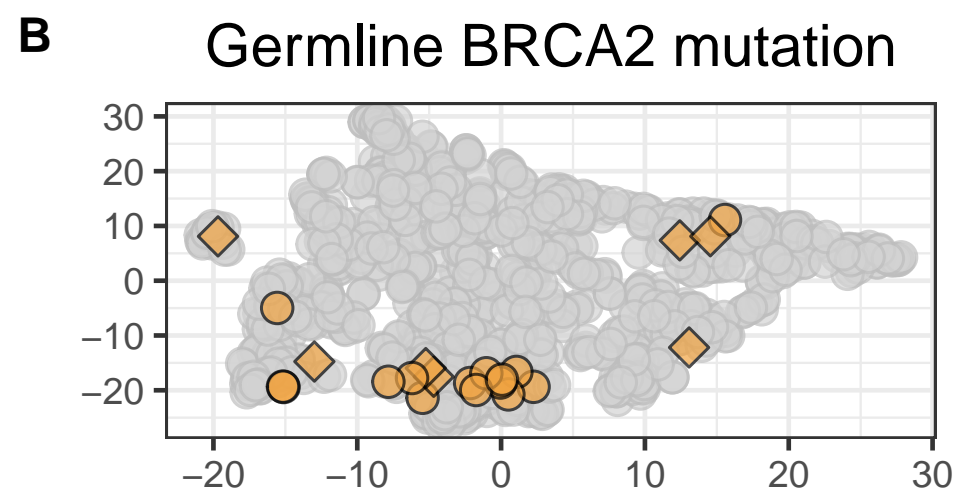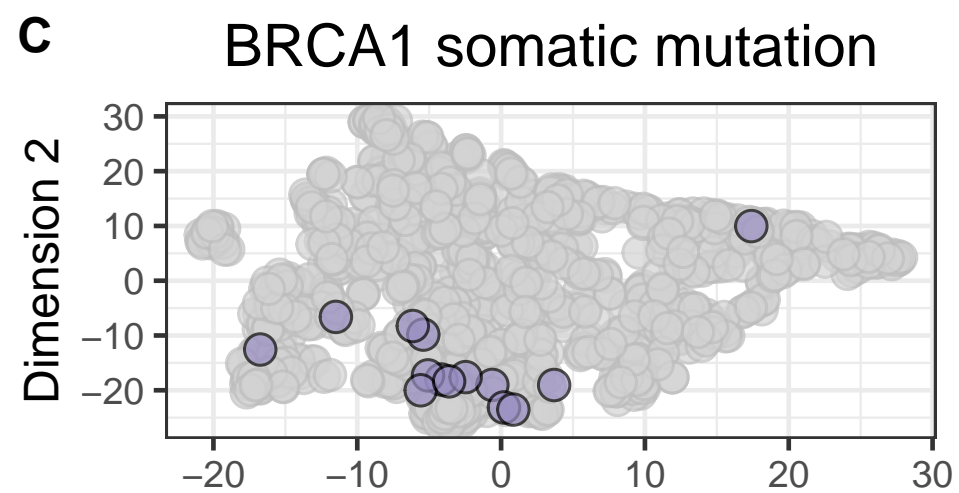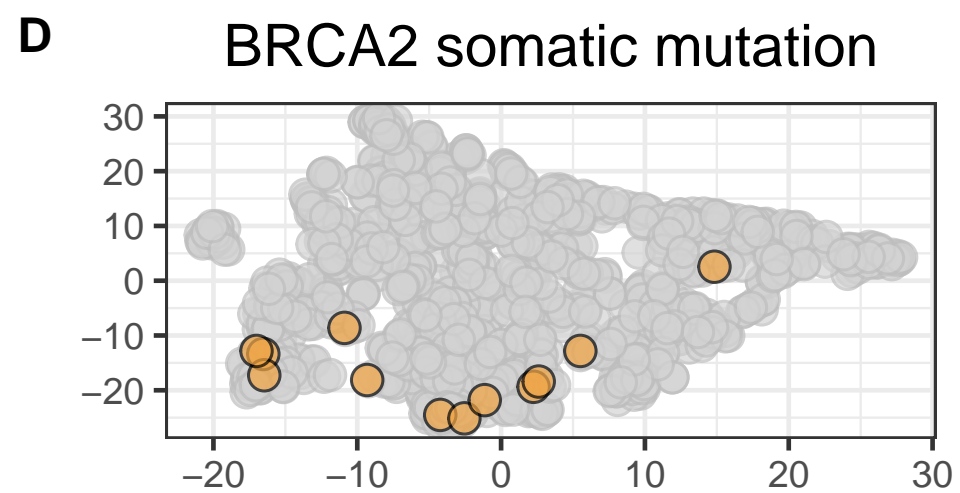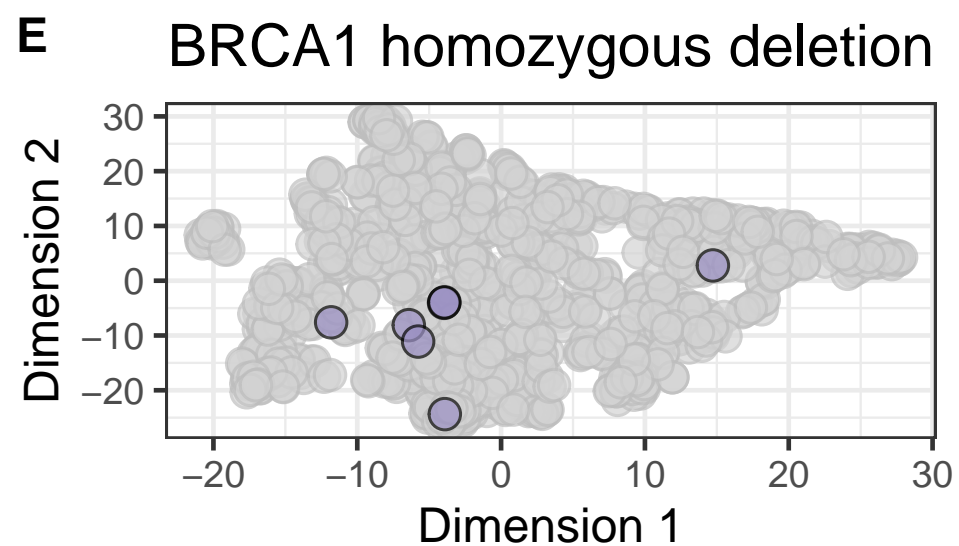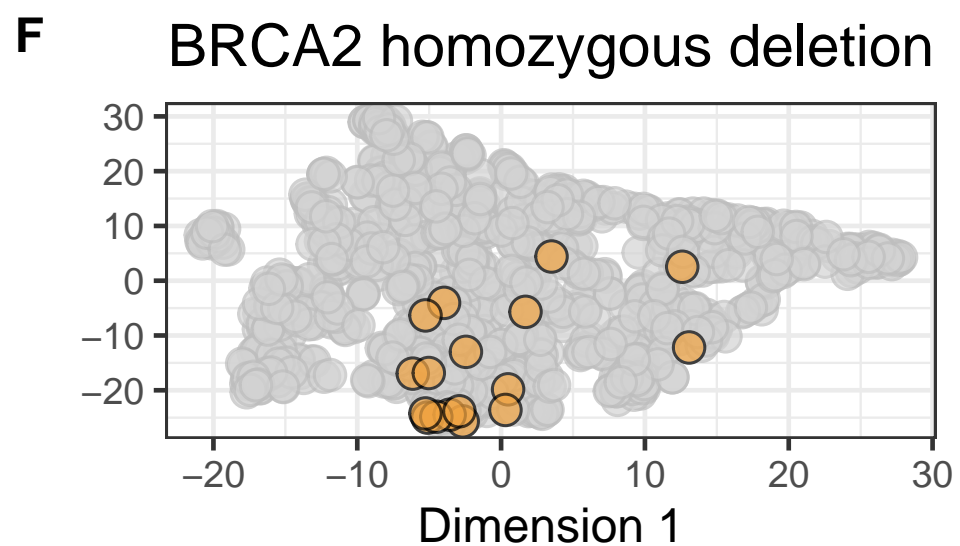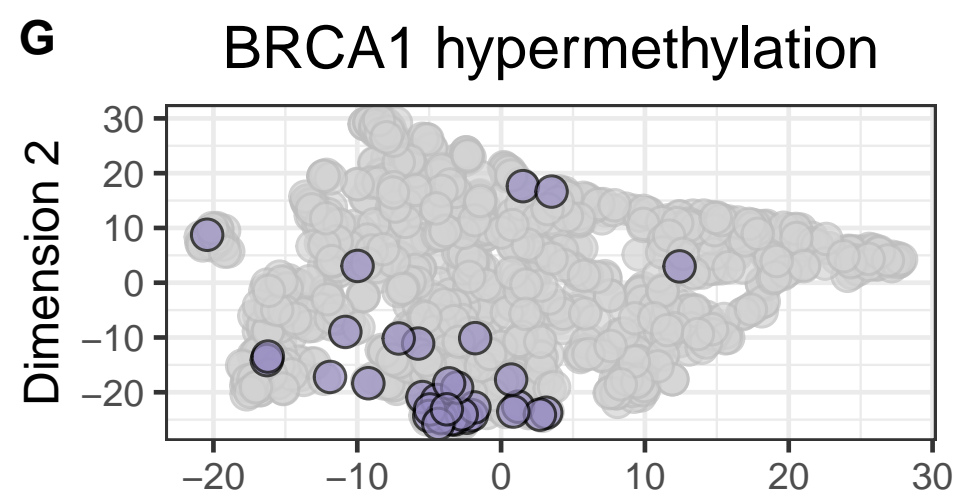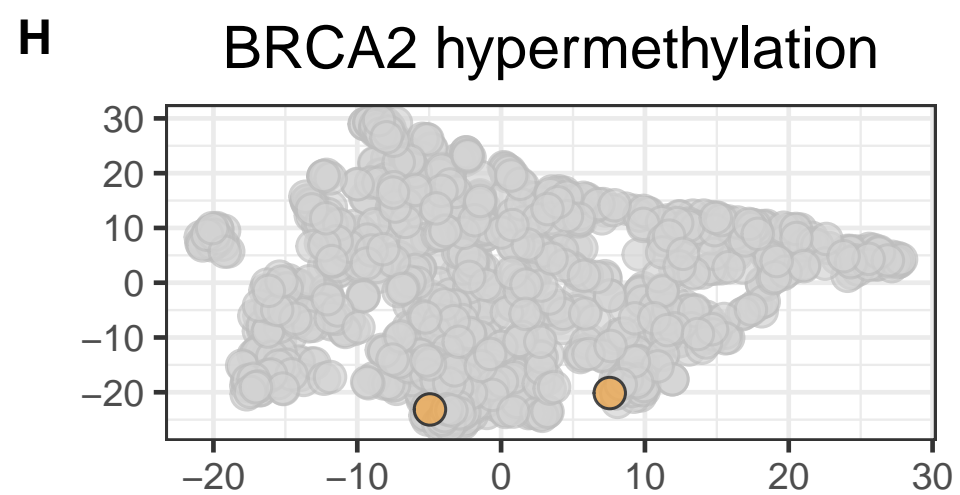

Supplement: S19 Fig — Using the same two-dimensional representation of mutational signatures shown in S10 Fig, this plot indicates which patients had germline mutations (A, B), somatic mutations (C, D), homozygous deletions (E, F), or hypermethylation events (G, H) in BRCA1 and BRCA2, respectively. Largely, these tumors had similar somatic-mutation signatures. Diamond shapes indicate patients for whom no* loss-of-heterozygosity was observed. Data are shown for all patients, even those for whom we did not have all types of data. (PDF) [file pone.0239197.s019.pdf]

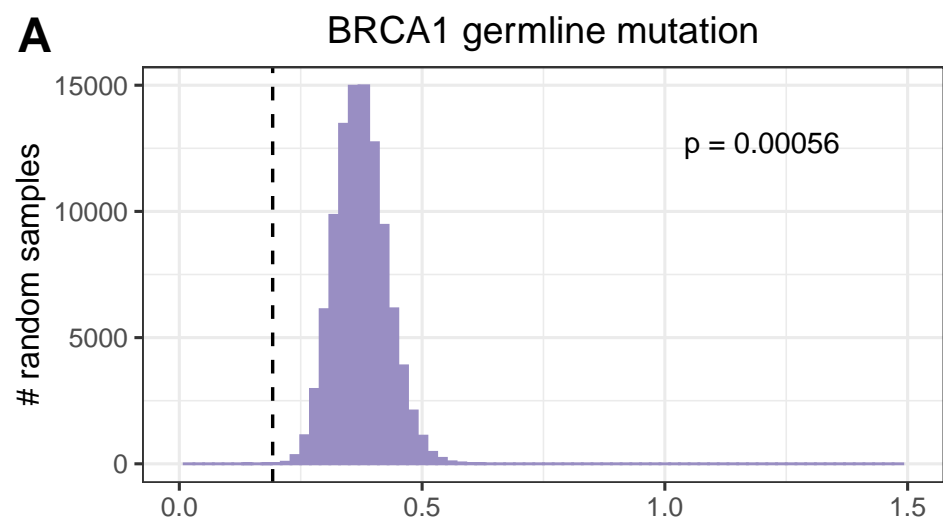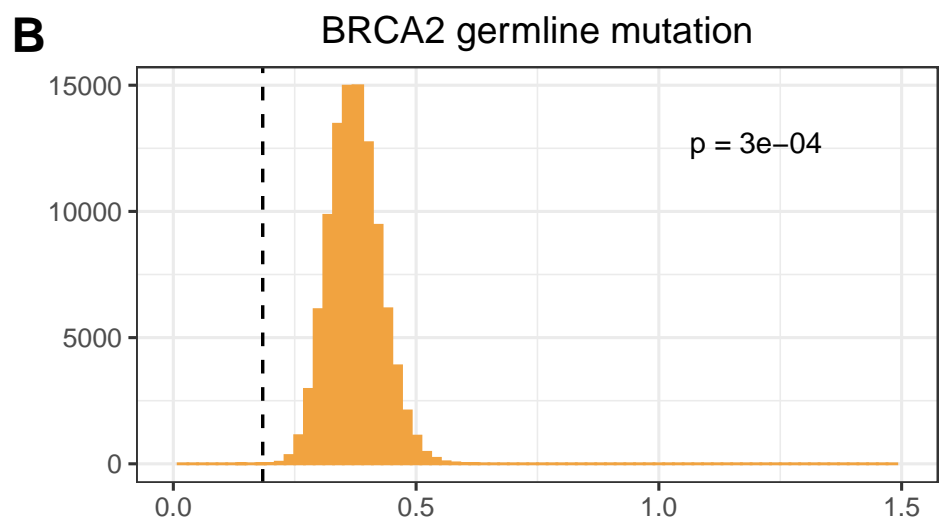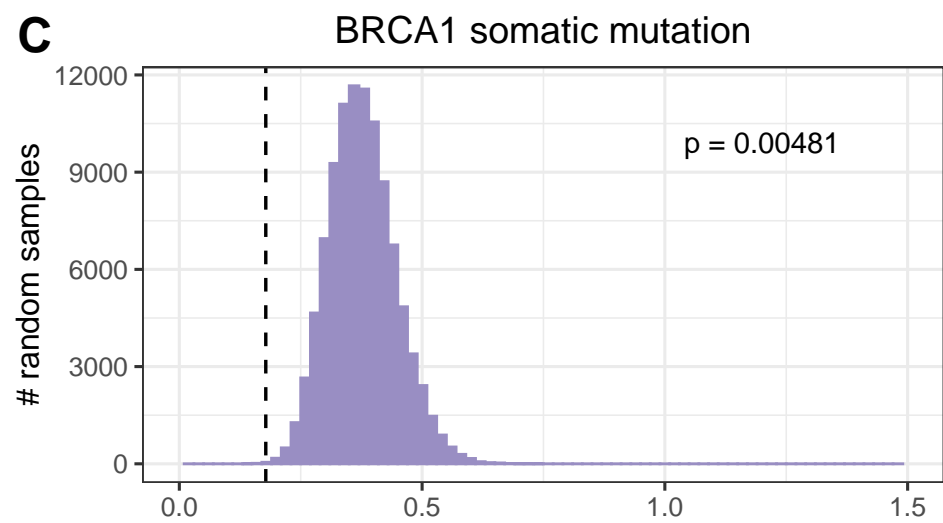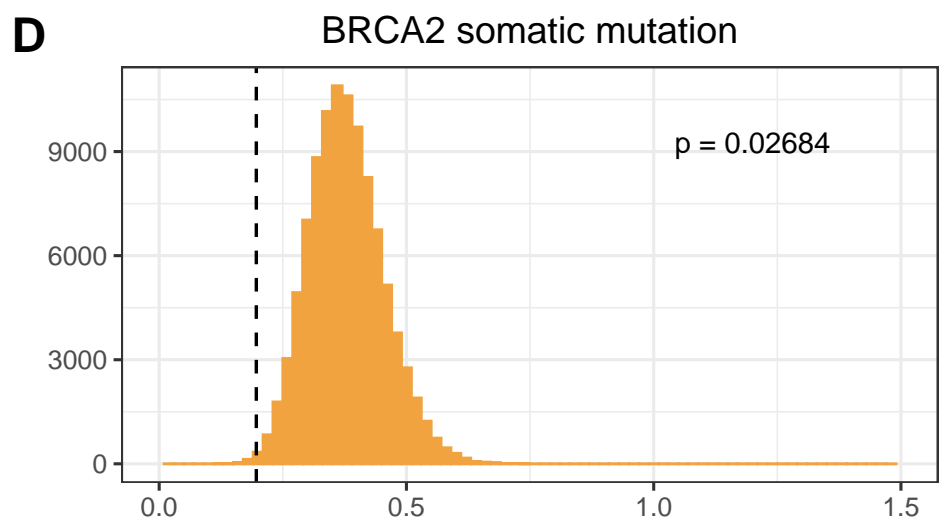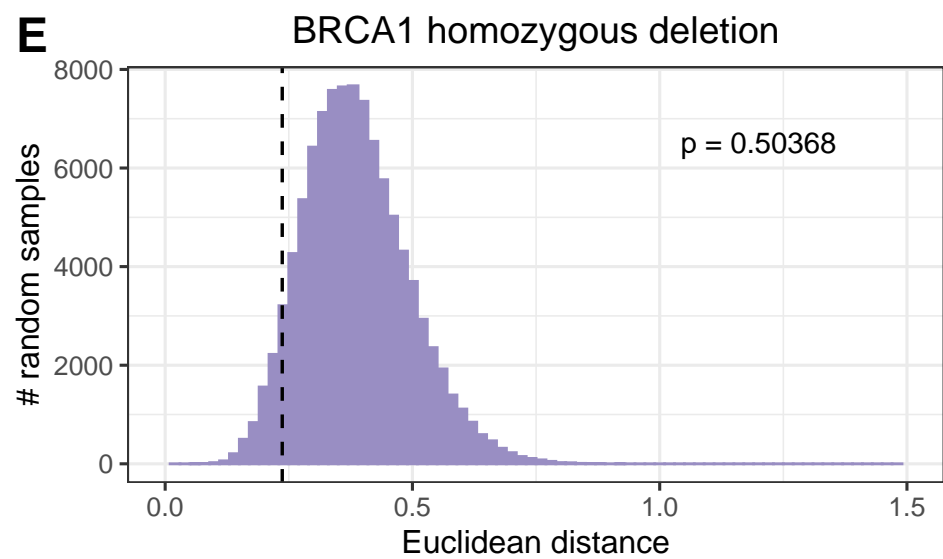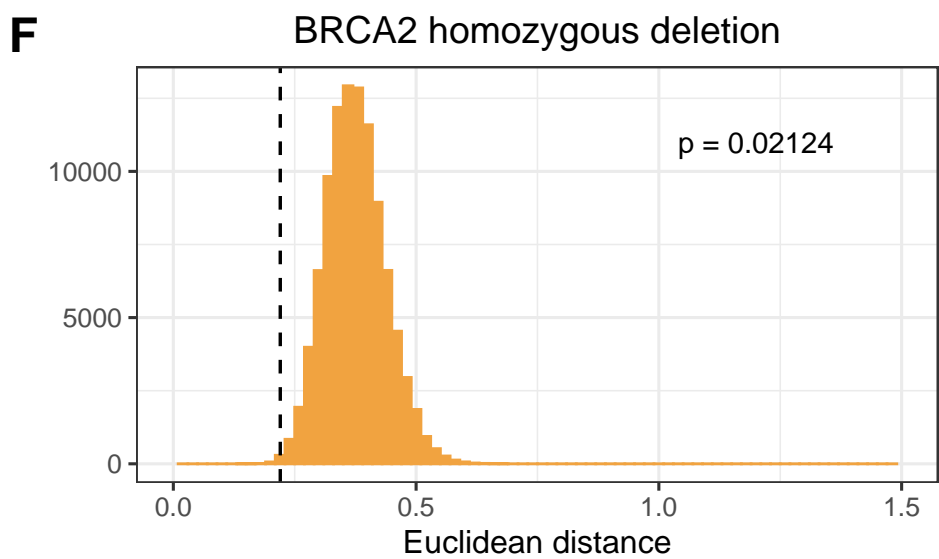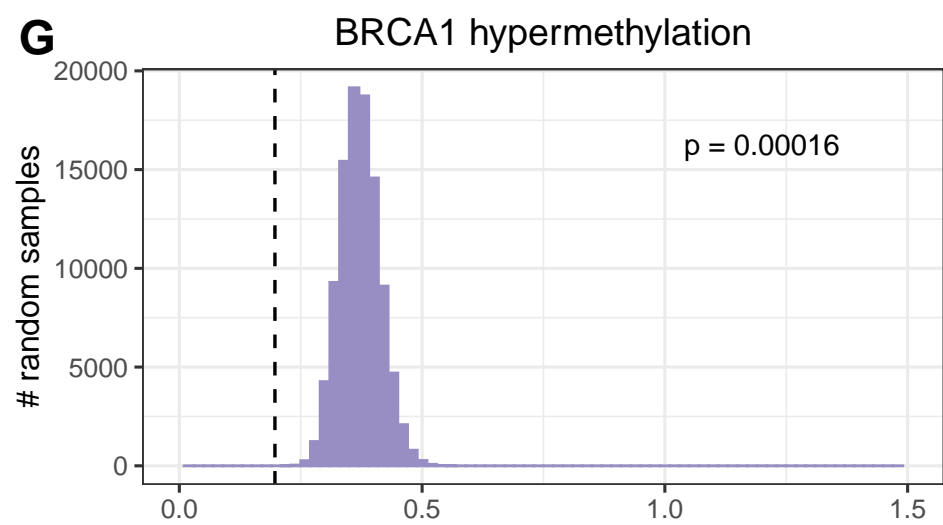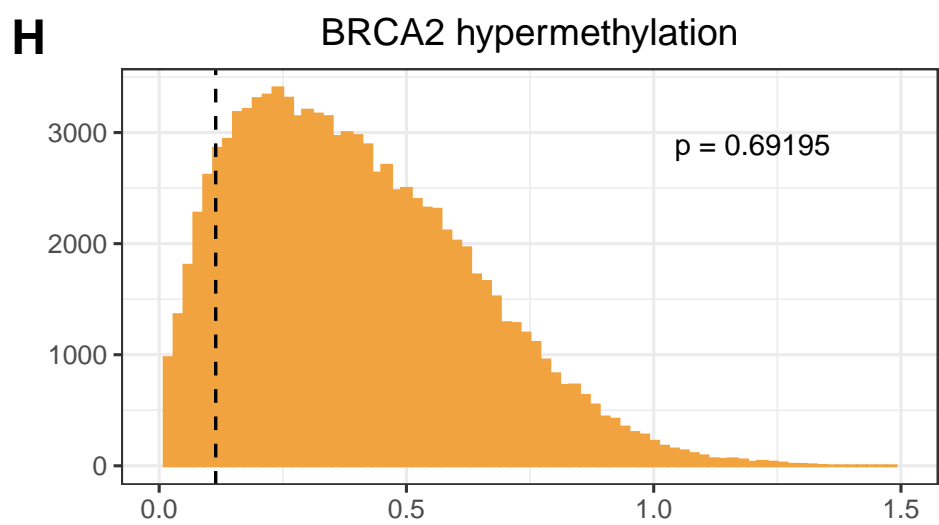

Supplement: S20 Fig — We calculated the Euclidean distance between each pair of individuals who had germline mutations (A, B), somatic mutations (C, D), homozygous deletions (E, F), or hypermethylation events (G, H) in BRCA1 or BRCA2; the medians of these distances are illustrated using vertical, dashed lines. We then randomized the patient identifiers and calculated pairwise distances for the same number of randomly selected patients, which resulted in an empirical null distribution. We calculated p-values by comparing the actual distances against the randomized distances and then adjusted for multiple tests using Holm’s method. (PDF) [file pone.0239197.s020.pdf]

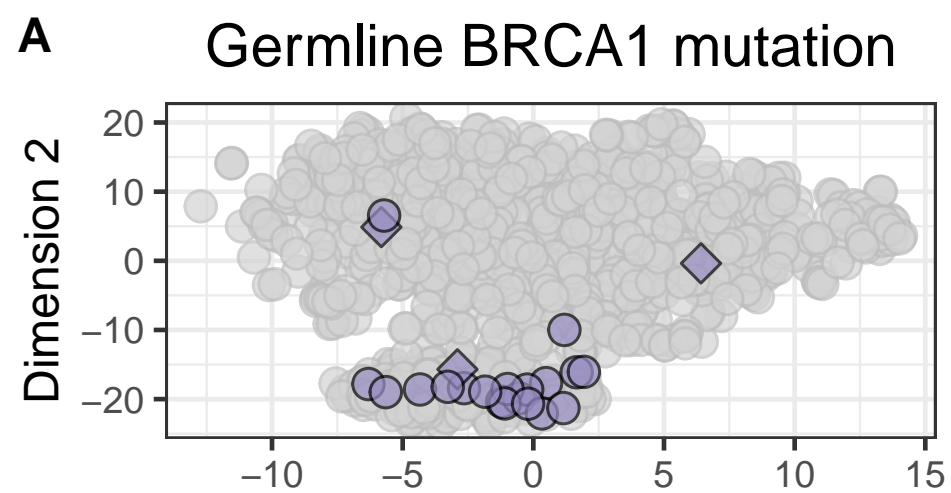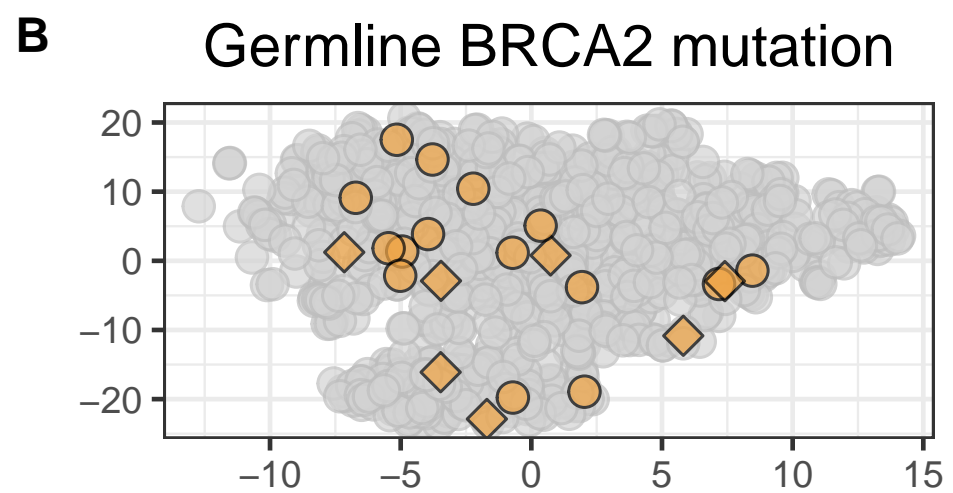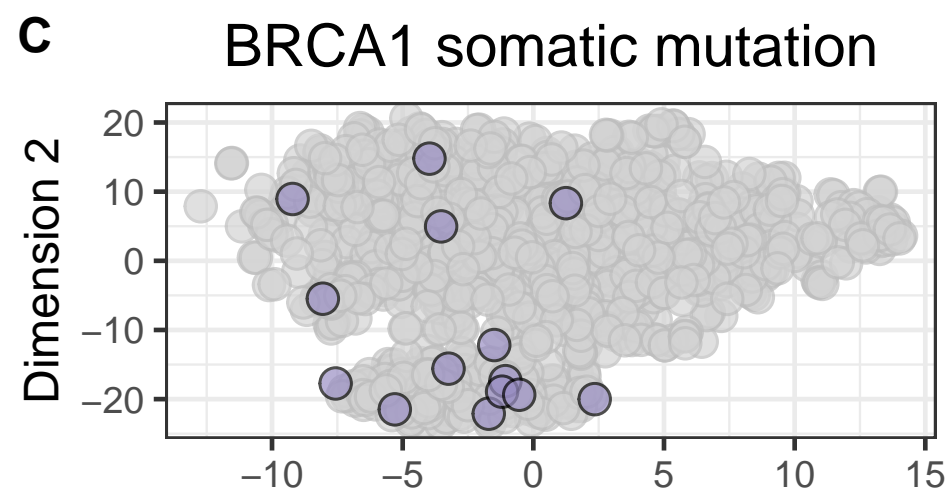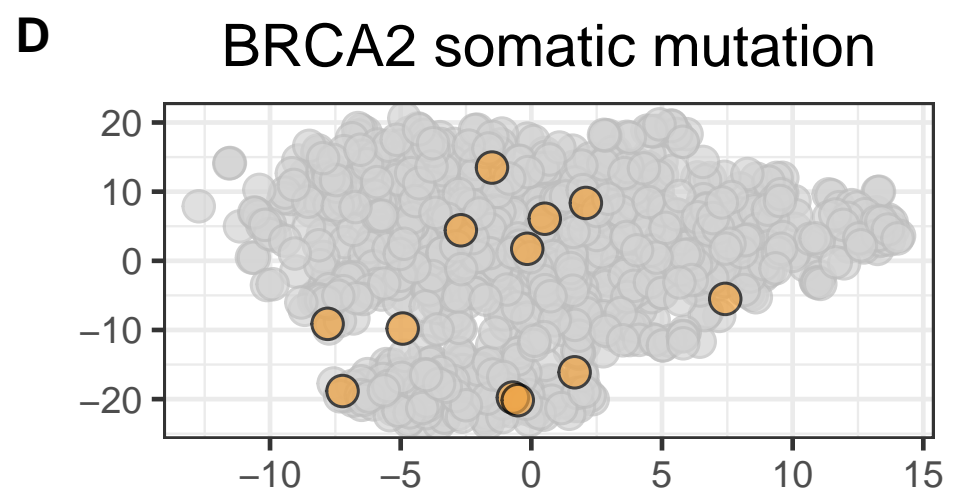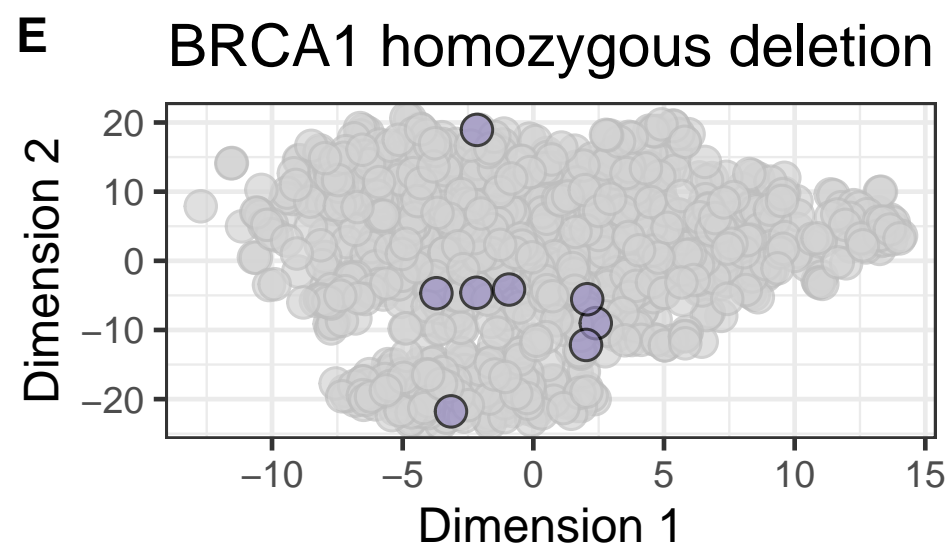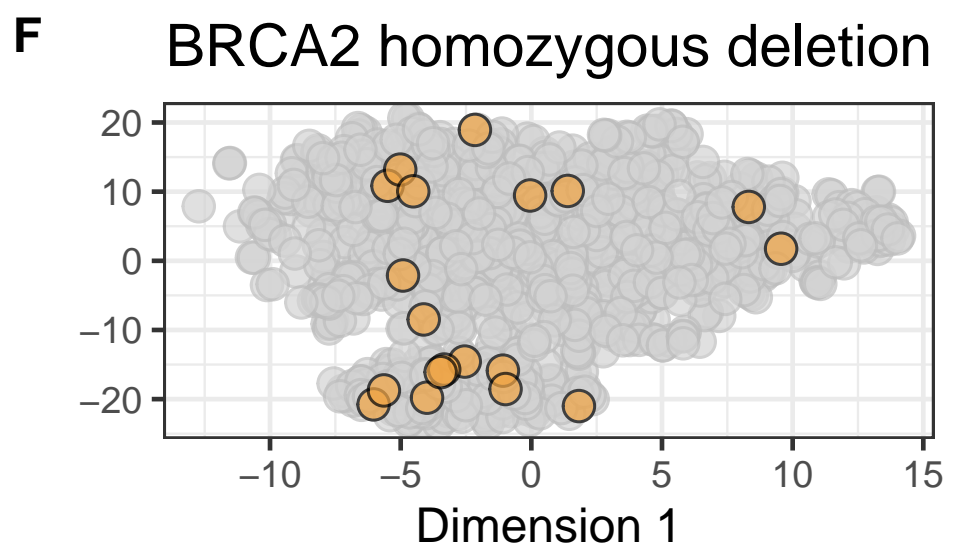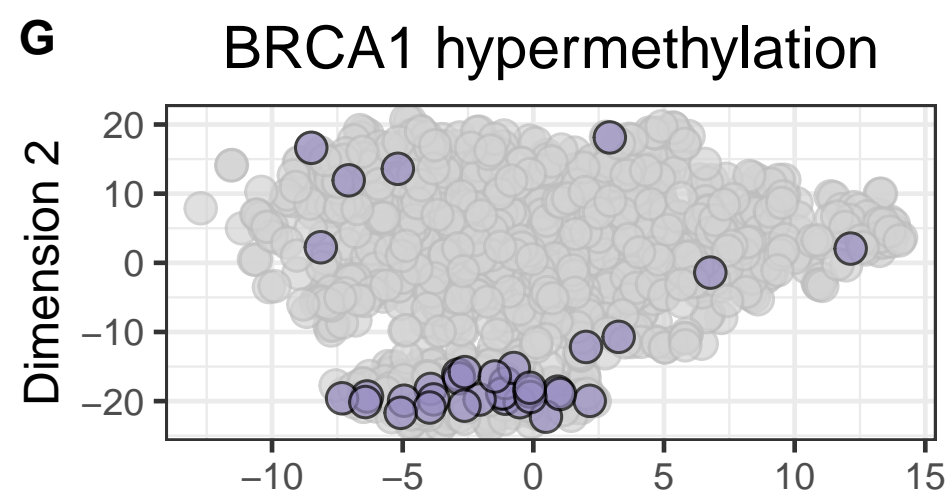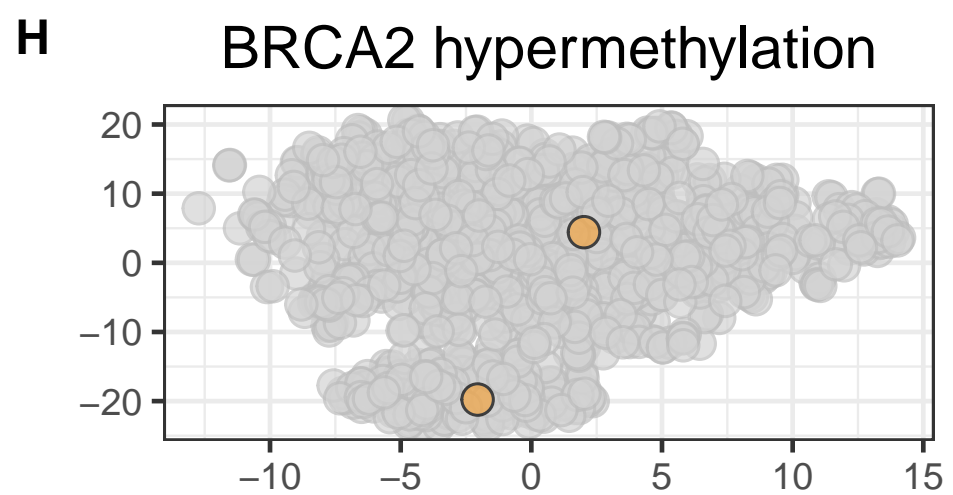

Supplement: S22 Fig — Using the same two-dimensional representation of gene-expression profiles shown in S11 Fig, this plot indicates which patients had germline mutations (A, B), somatic mutations (C, D), homozygous deletions (E, F), or hypermethylation events (G, H) in BRCA1 and BRCA2, respectively. Many of these tumors overlapped with the Basal-like subtype, but other tumors were dispersed broadly across the gene-expression landscape. Diamond shapes indicate patients for whom no loss-of-heterozygosity was observed. Data are shown for all patients, even those for whom we did not have all types of data. (PDF) [file pone.0239197.s022.pdf]

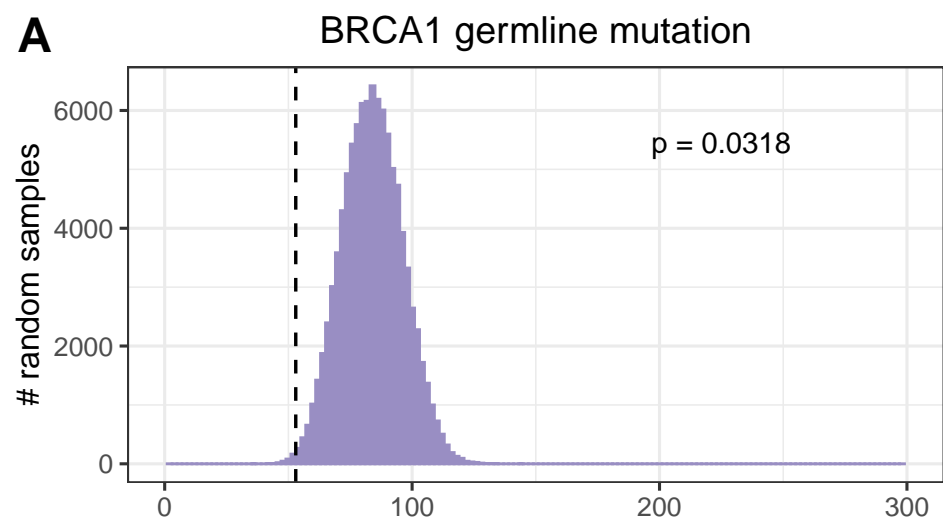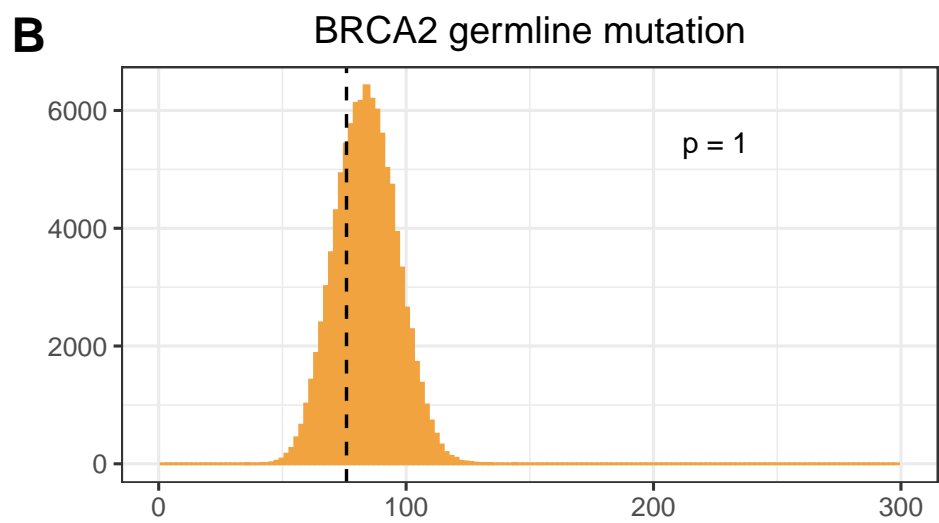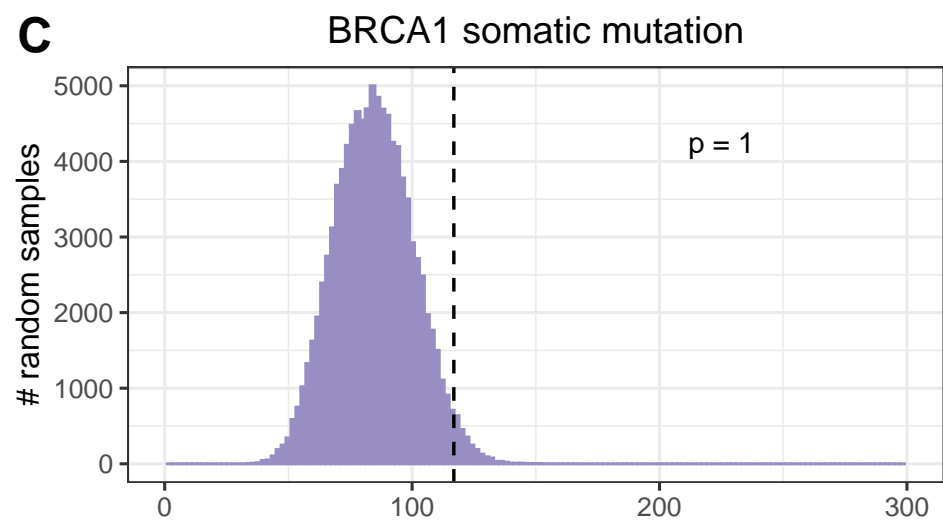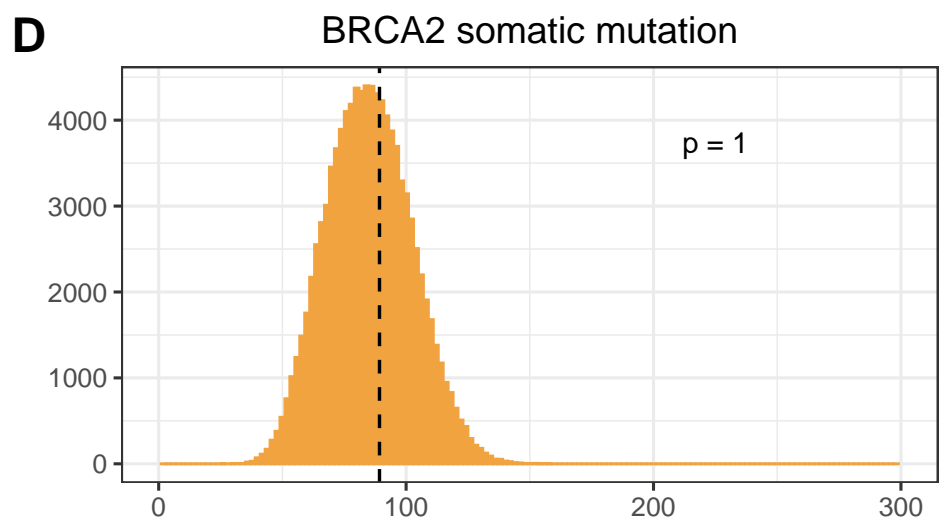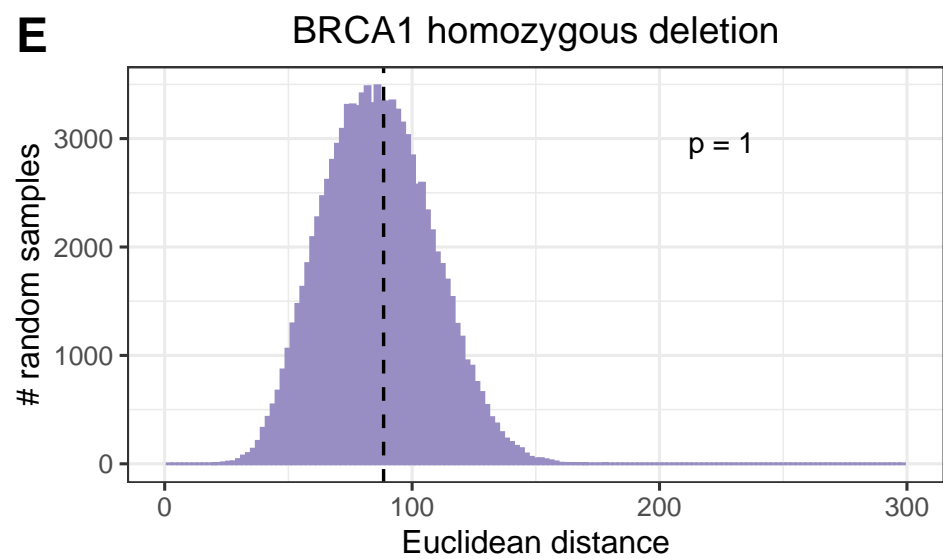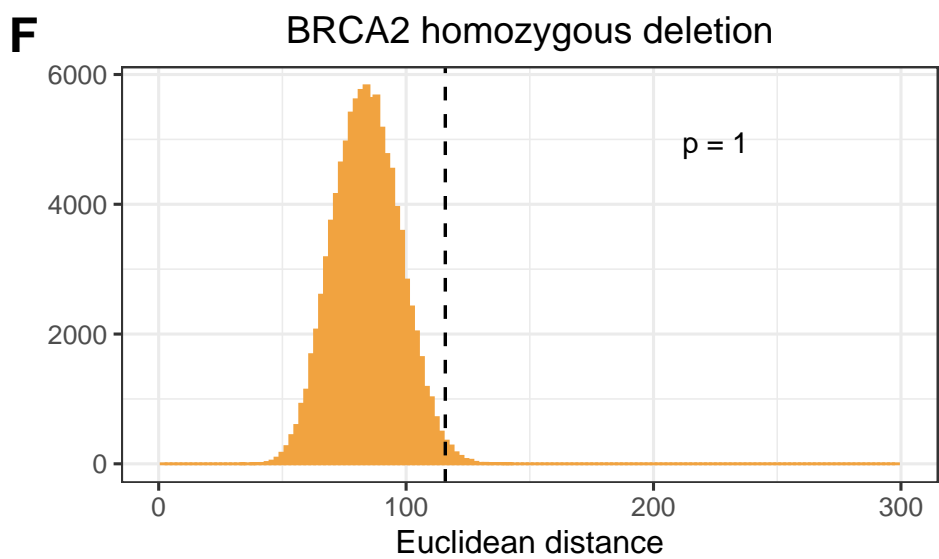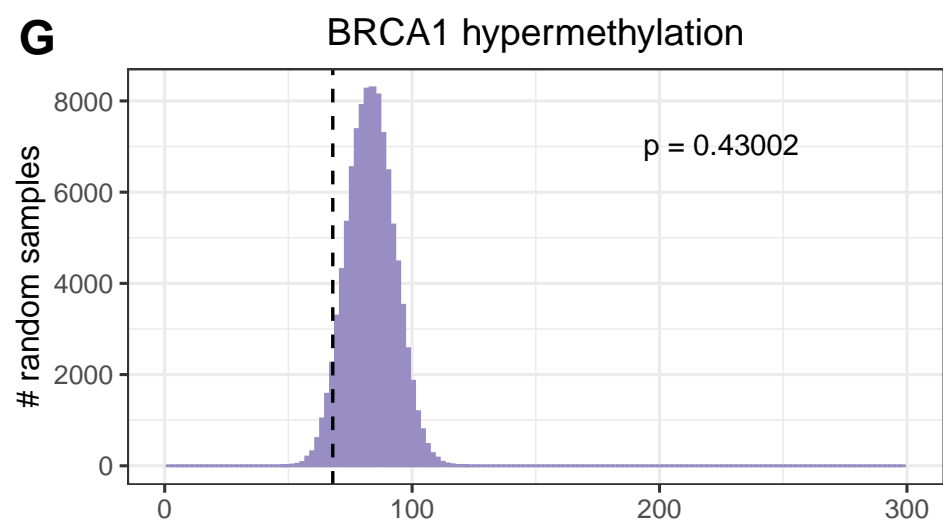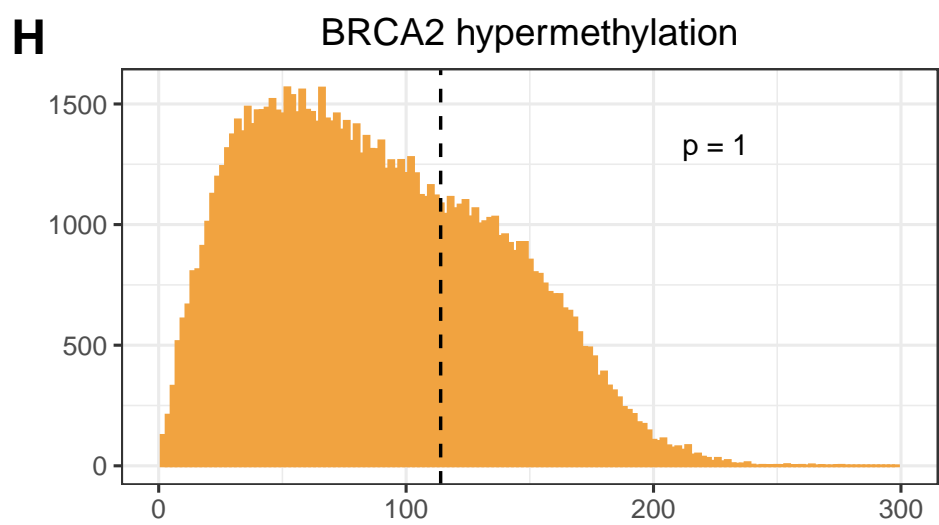

Supplement: S23 Fig — We calculated the Euclidean distance between each pair of individuals who had germline mutations (A, B), somatic mutations (C, D), homozygous deletions (E, F), or hypermethylation events (G, H) in BRCA1 or BRCA2; the medians of these distances are illustrated using vertical, dashed lines. We then randomized the patient identifiers and calculated pairwise distances for the same number of randomly selected patients, which resulted in an empirical null distribution. We calculated p-values by comparing the actual distances against the randomized distances and then adjusted for multiple tests using Holm’s method. (PDF) [file pone.0239197.s023.pdf]

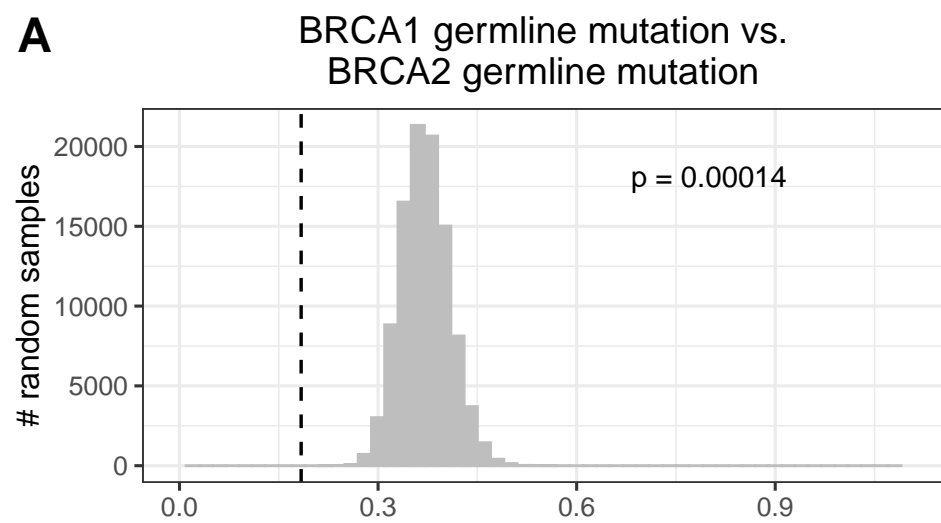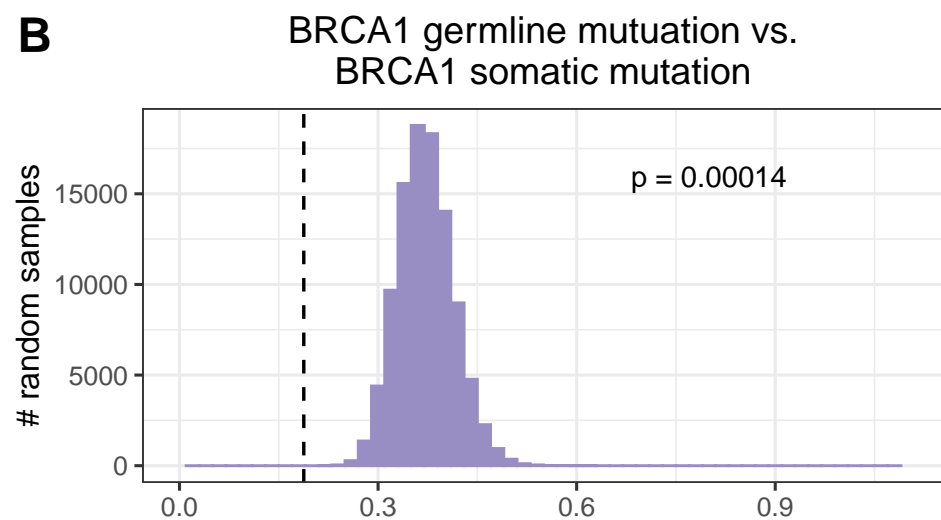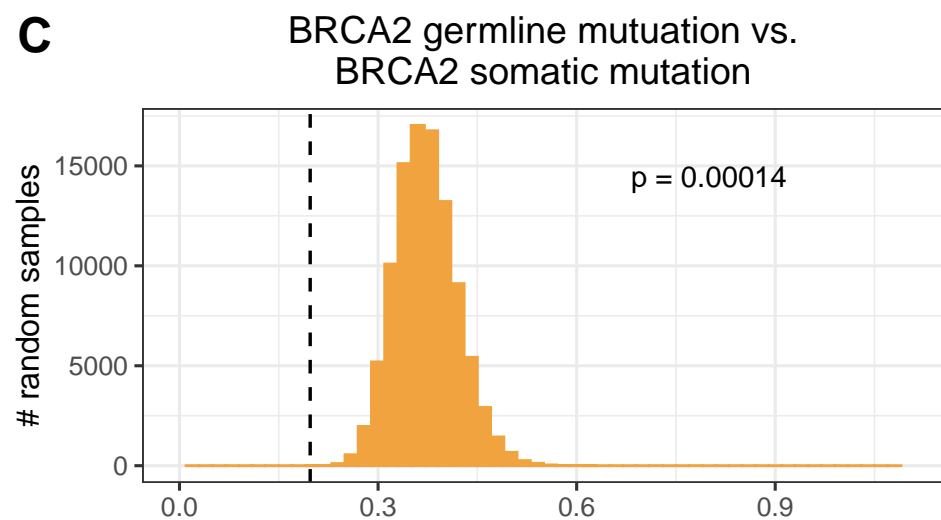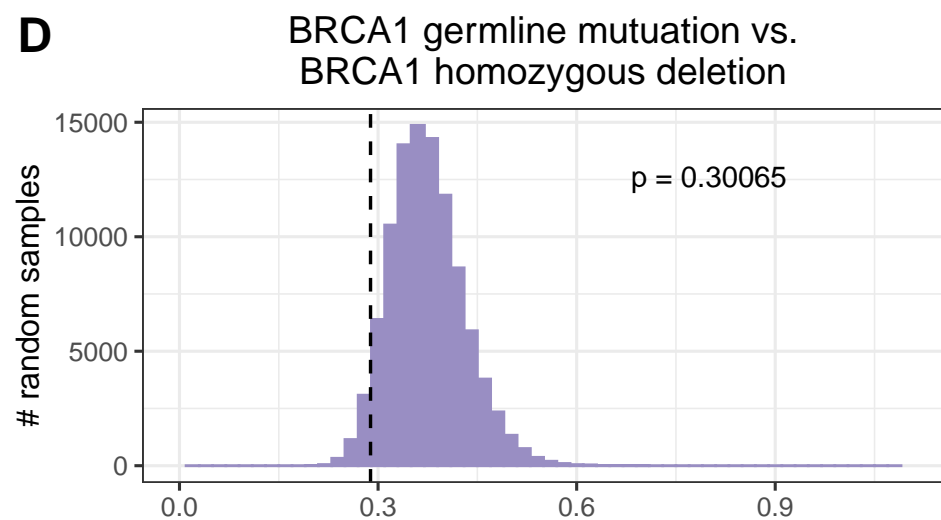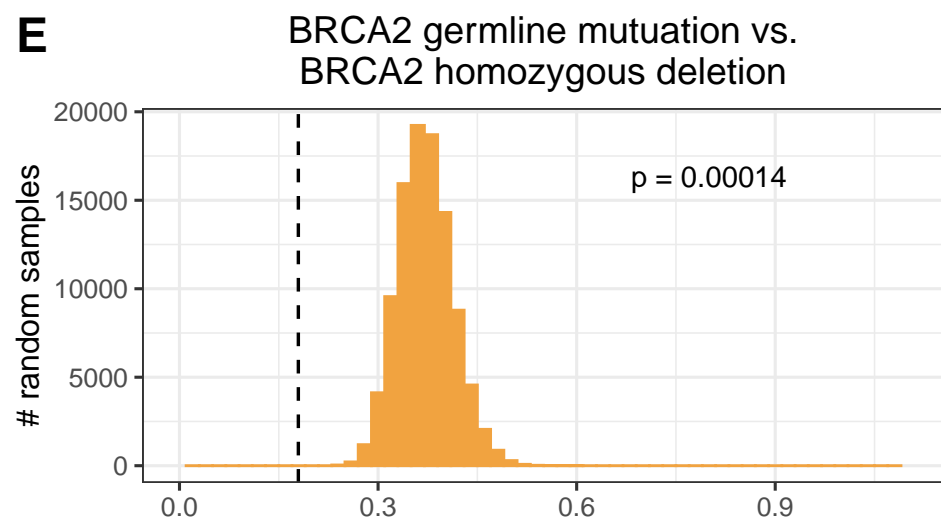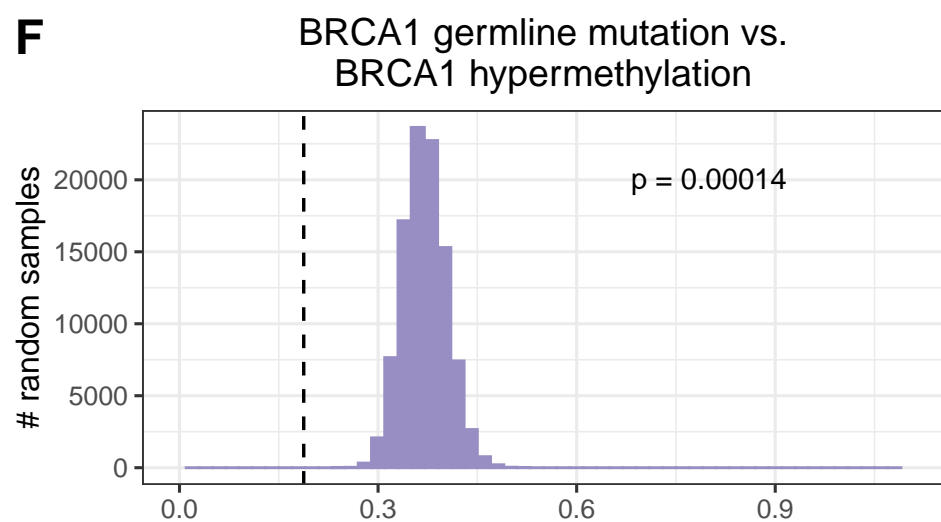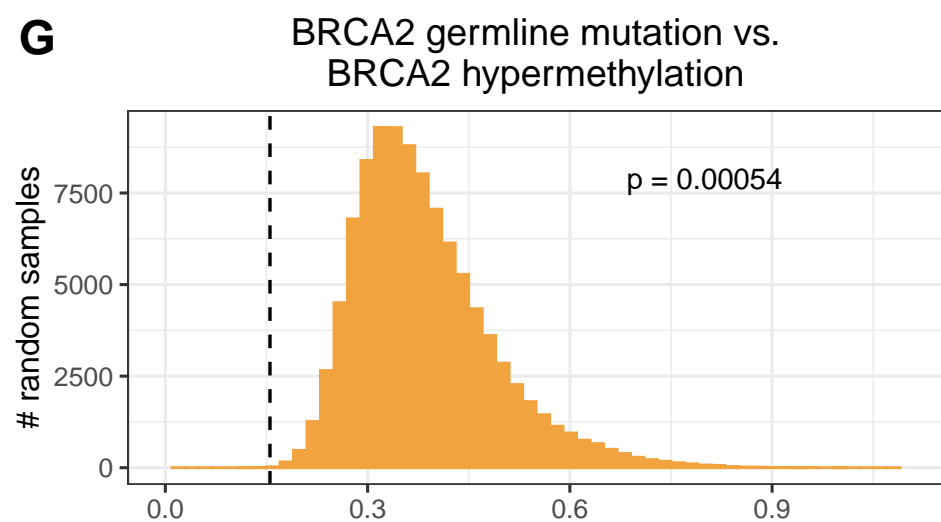

Supplement: S24 Fig — We identified patients who had a germline mutation in BRCA1 or BRCA2 and compared them against each other (A), those with a somatic mutation in the same gene (B-C), those with a homozygous deletion in the same gene (D-E) and those with DNA hypermethylation of the same gene (F-G). We calculated the Euclidean distance between each pair of individuals in these groups; these distances are illustrated using vertical, dashed lines. We then randomized the patient identifiers and calculated pairwise distances for groups of randomly selected patients, which resulted in an empirical null distribution. We calculated p-values by comparing the actual distances against the randomized distances. (PDF) [file pone.0239197.s024.pdf]

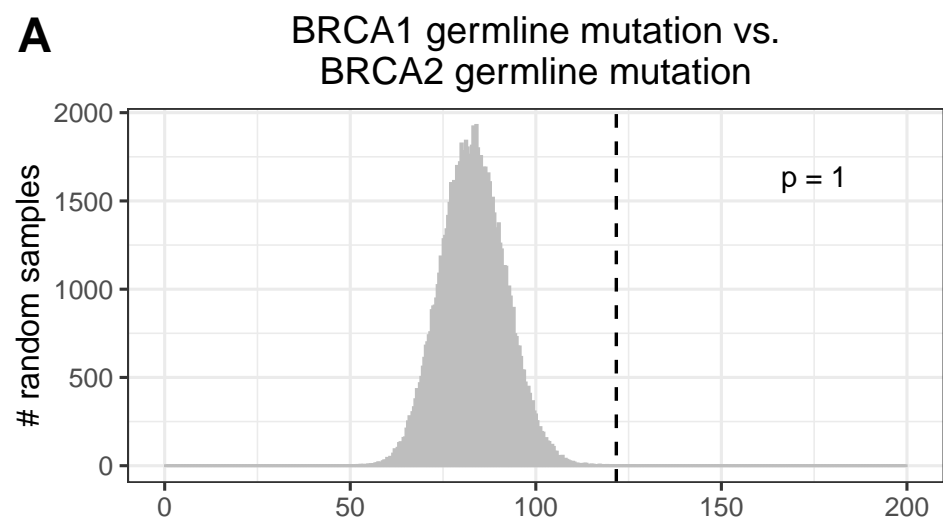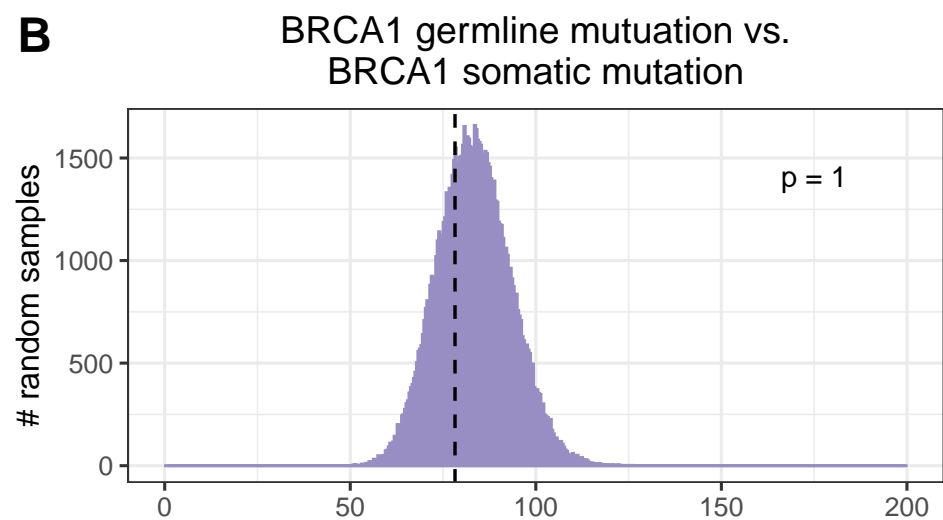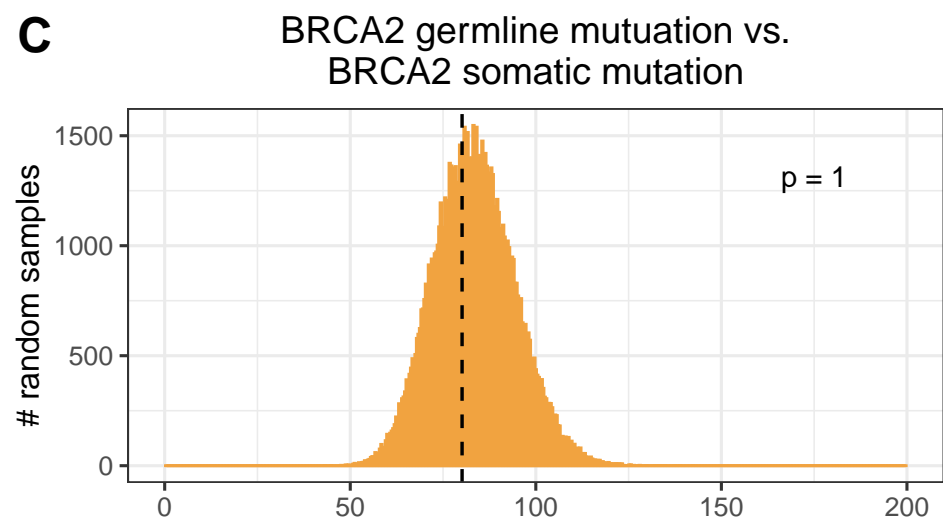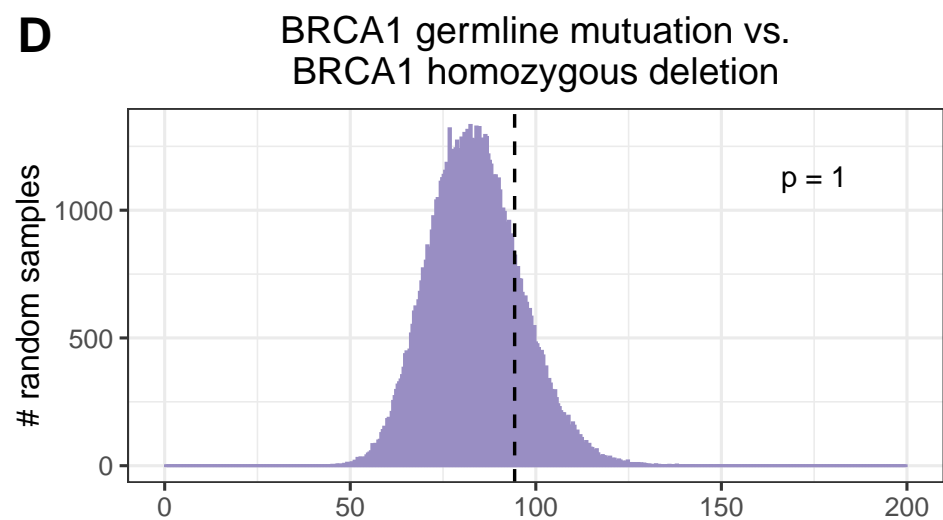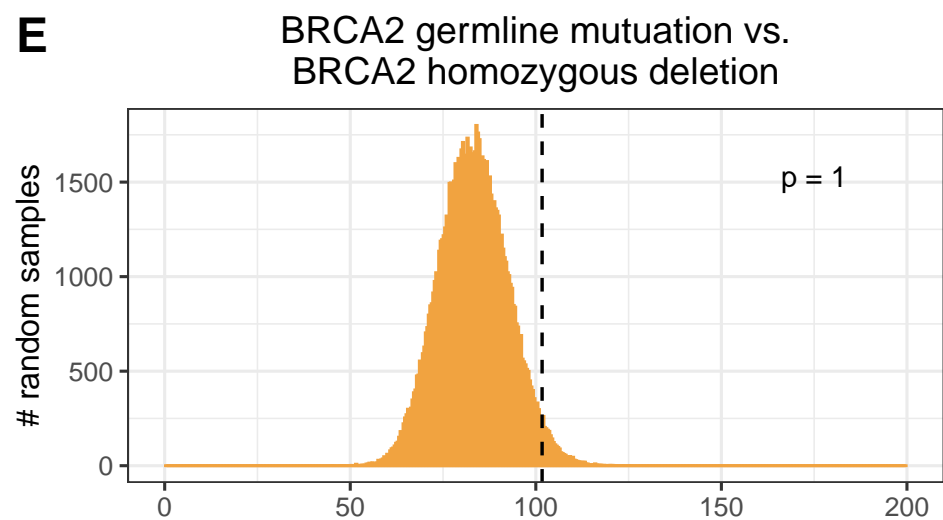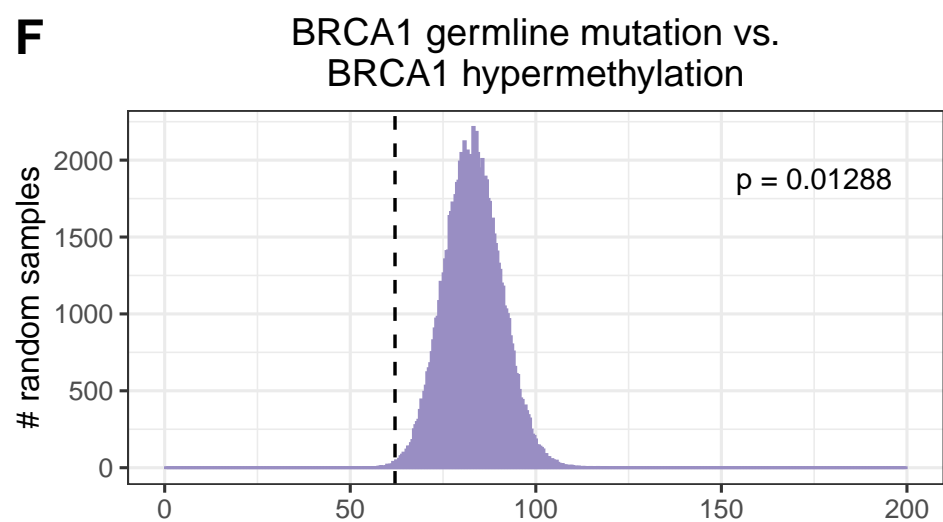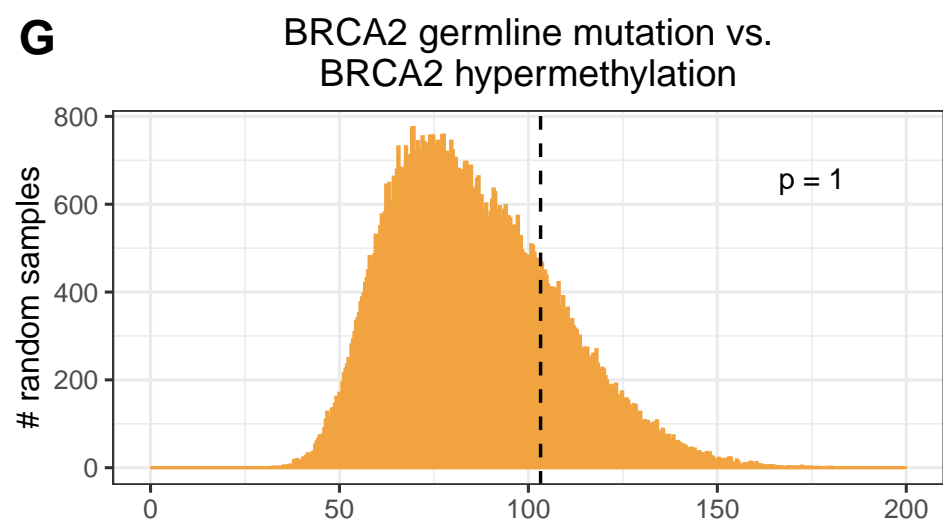

Supplement: S25 Fig — We identified patients who had a germline mutation in BRCA1 or BRCA2 and compared them against each other (A), those with a somatic mutation in the same gene (B-C), those with a homozygous deletion in the same gene (D-E) and those with DNA hypermethylation of the same gene (F-G). We calculated the Euclidean distance between each pair of individuals in these groups; these distances are illustrated using vertical, dashed lines. We then randomized the patient identifiers and calculated pairwise distances for groups of randomly selected patients, which resulted in an empirical null distribution. We calculated p-values by comparing the actual distances against the randomized distances. (PDF) [file pone.0239197.s025.pdf]

BRCA1 / BRCA2, multiple aberration types

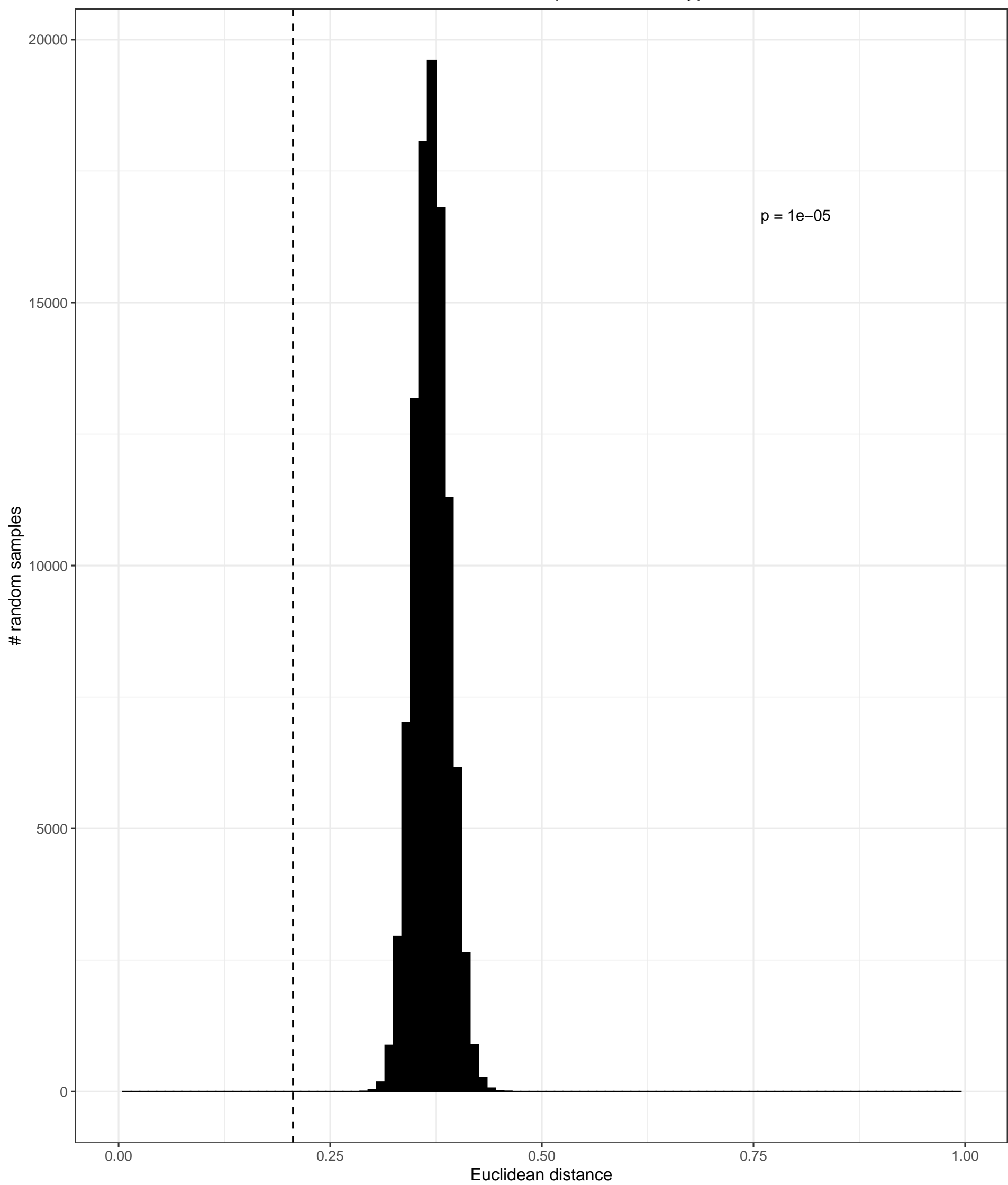

Supplement: S26 Fig — We calculated the Euclidean distance between each pair of individuals who had a germline mutation, somatic mutation, homozygous deletion, and/or hypermethylation event in BRCA1 and/or BRCA2; the median of these distances is illustrated using a vertical, dashed line. We then randomized the patient identifiers and calculated pairwise distances for the same number of randomly selected patients, which resulted in an empirical null distribution. We calculated a p-value by comparing the actual distance against the randomized distances. (PDF) [file pone.0239197.s026.pdf]

# Germline mutations

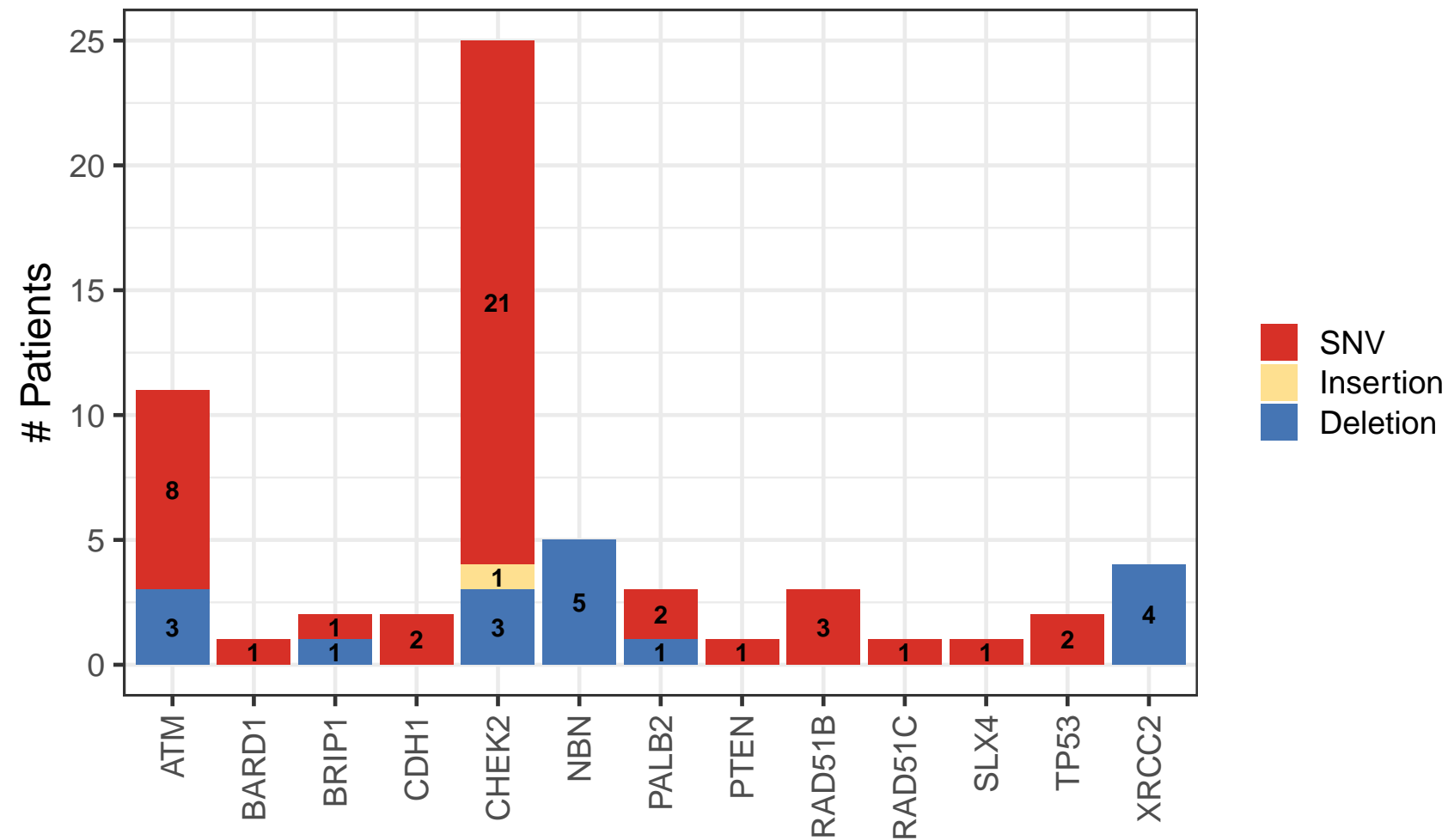

Supplement: S27 Fig — This graph omits genes in which we observed no germline mutations. SNV = single-nucleotide variant. (PDF) [file pone.0239197.s027.pdf]

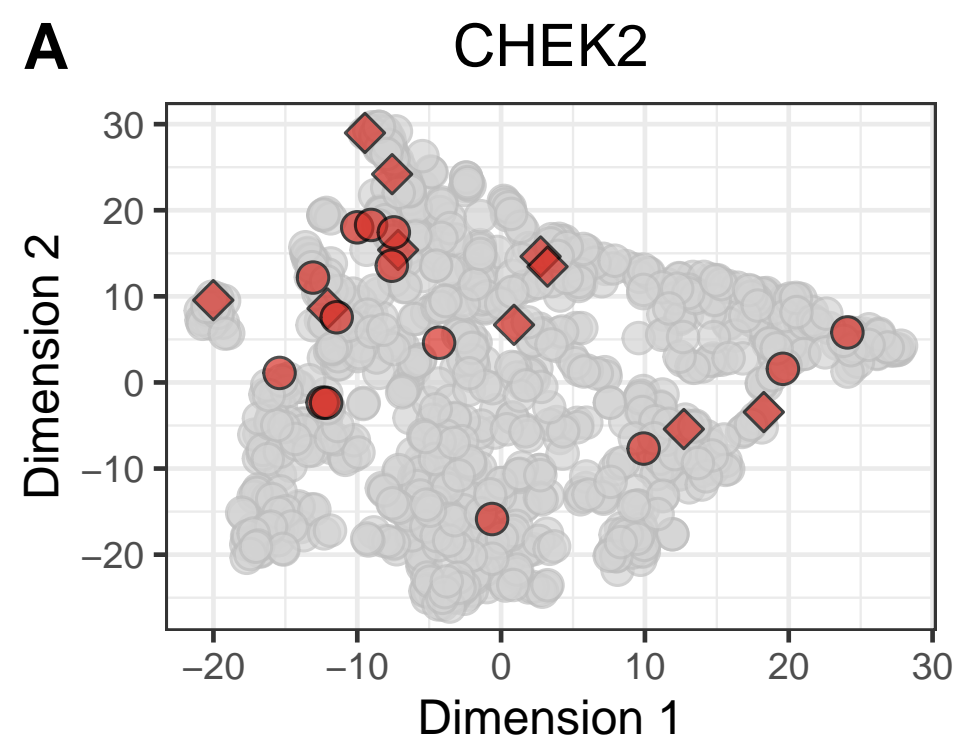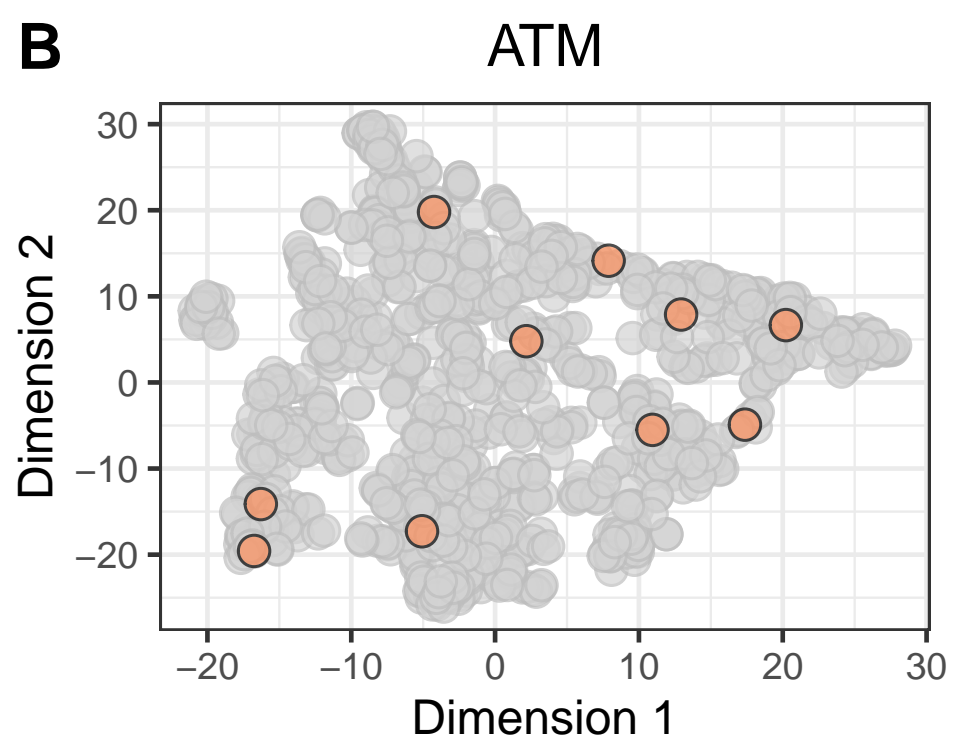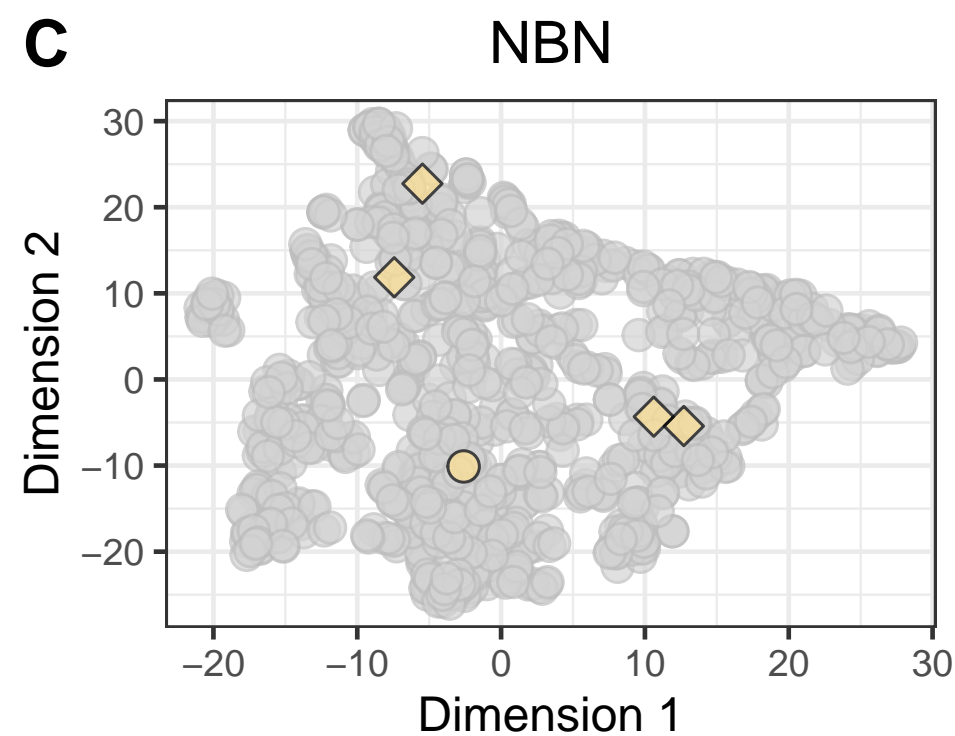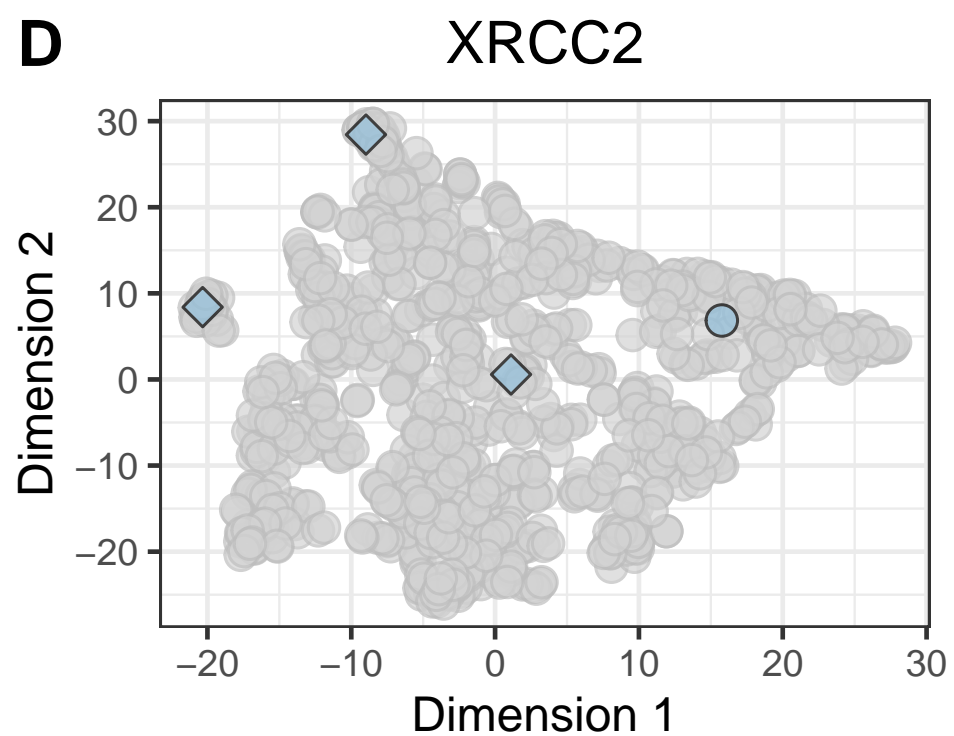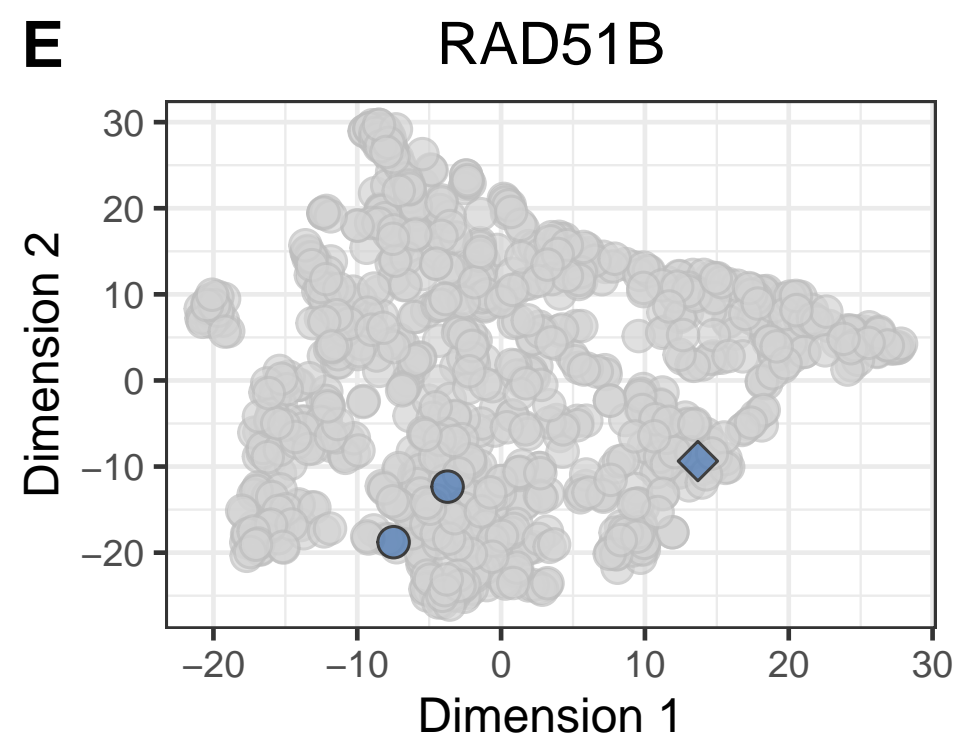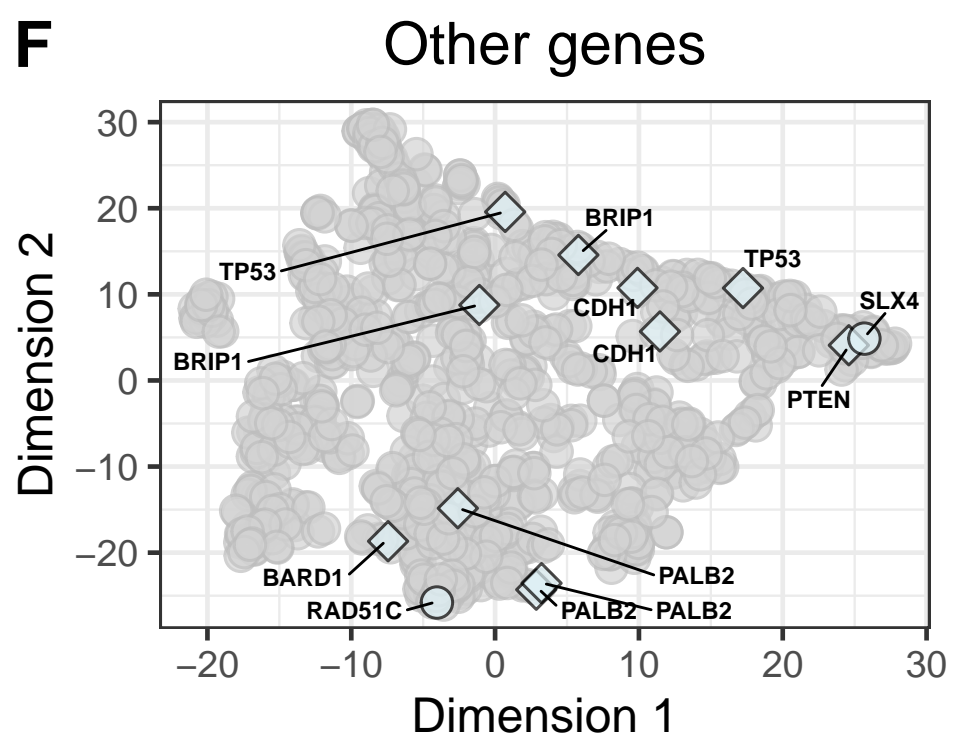

Supplement: S28 Fig — Using the same two-dimensional representation of mutational signatures shown in S10 Fig, this plot indicates which patients had germline mutations in non-BRCA cancer-predisposition genes. Diamond shapes indicate patients for whom no loss-of-heterozygosity was observed. (PDF) [file pone.0239197.s028.pdf]

# Somatic mutations

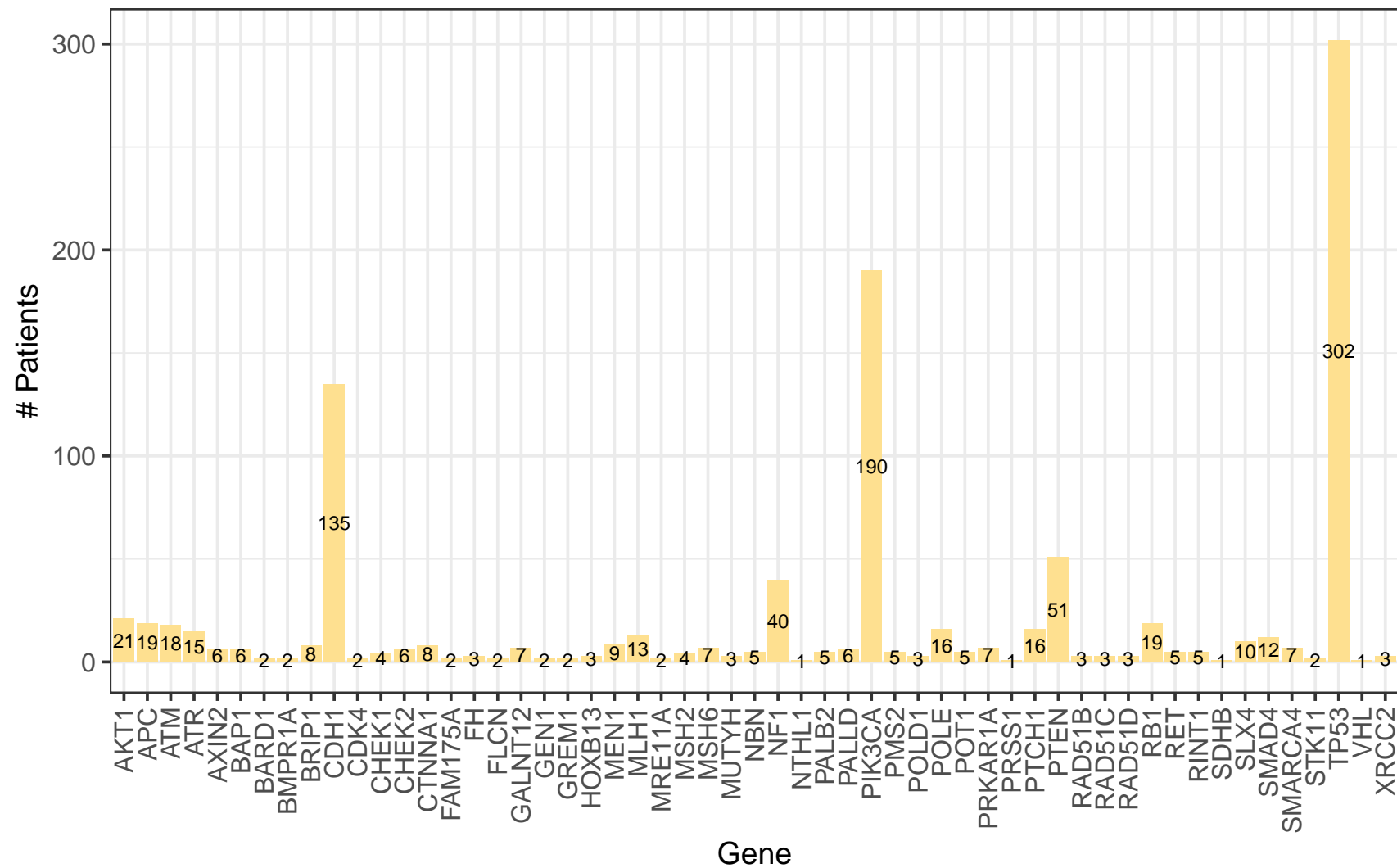

Supplement: S29 Fig — This graph omits genes in which we observed no somatic mutations. (PDF) [file pone.0239197.s029.pdf]

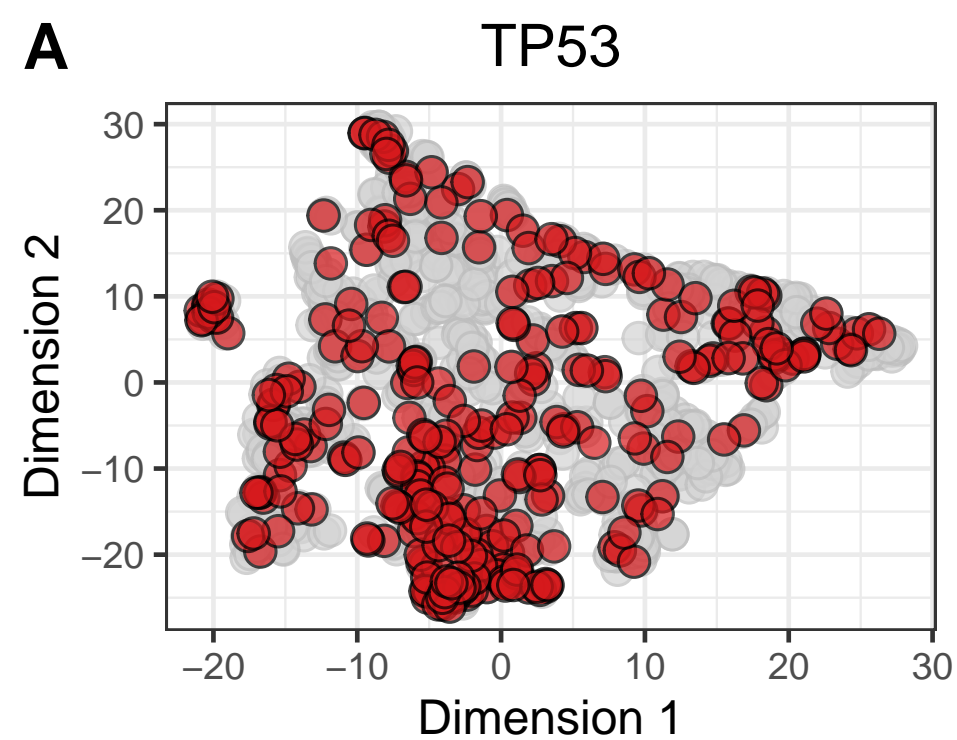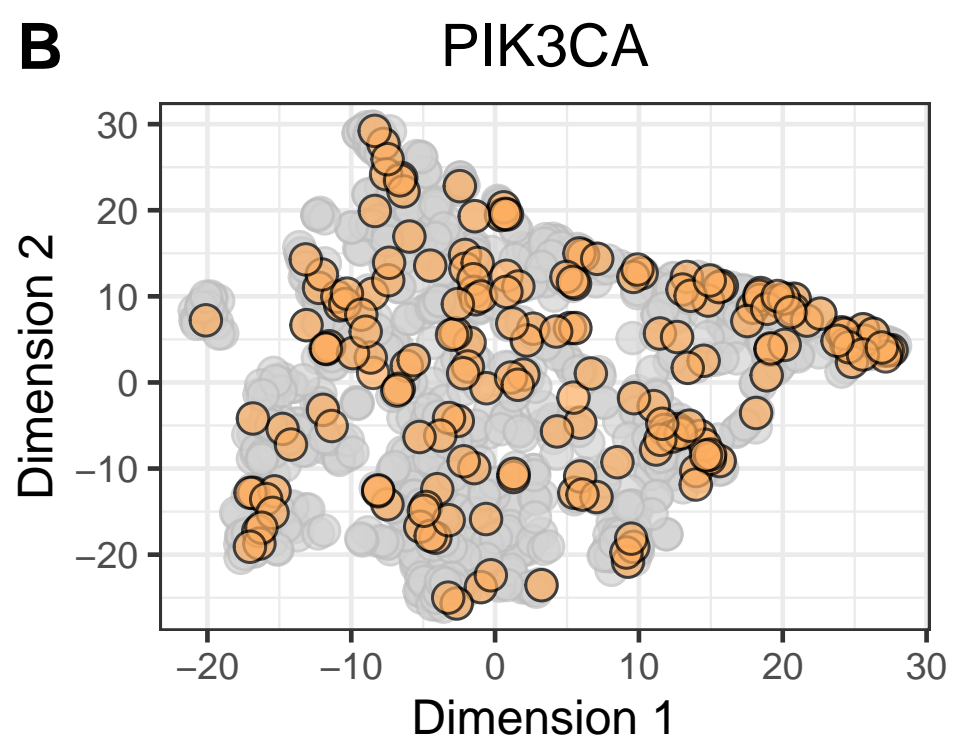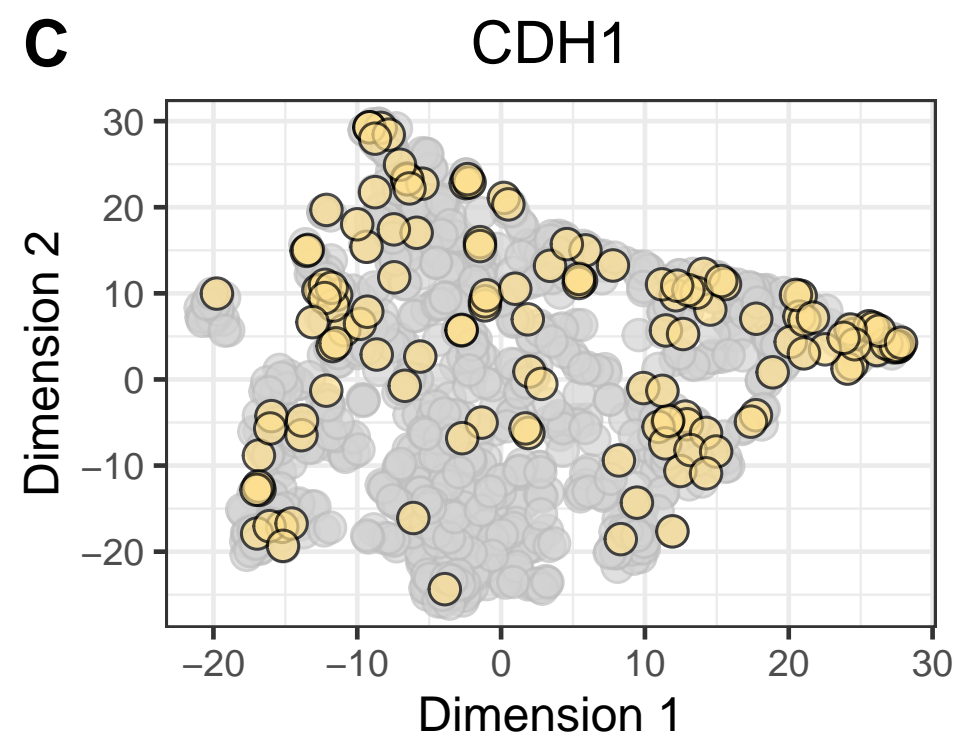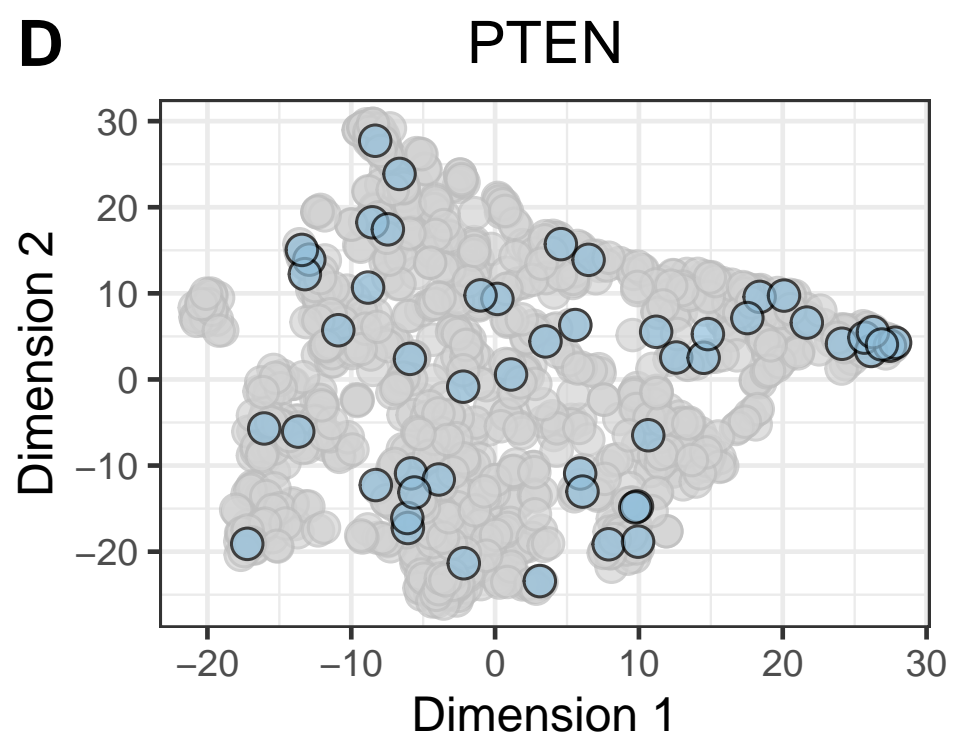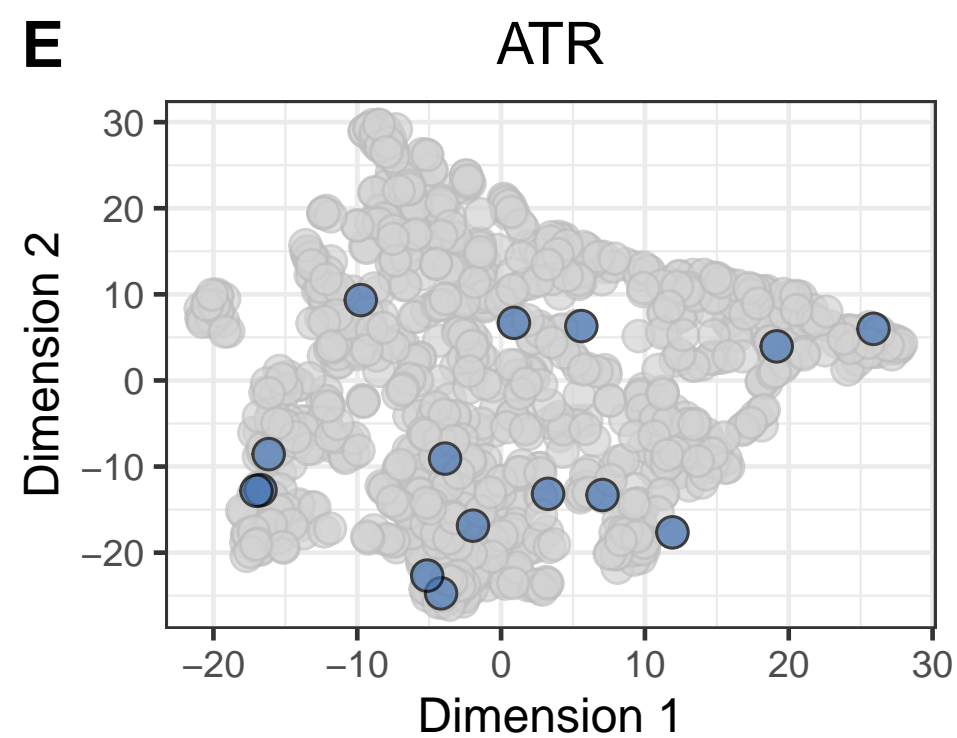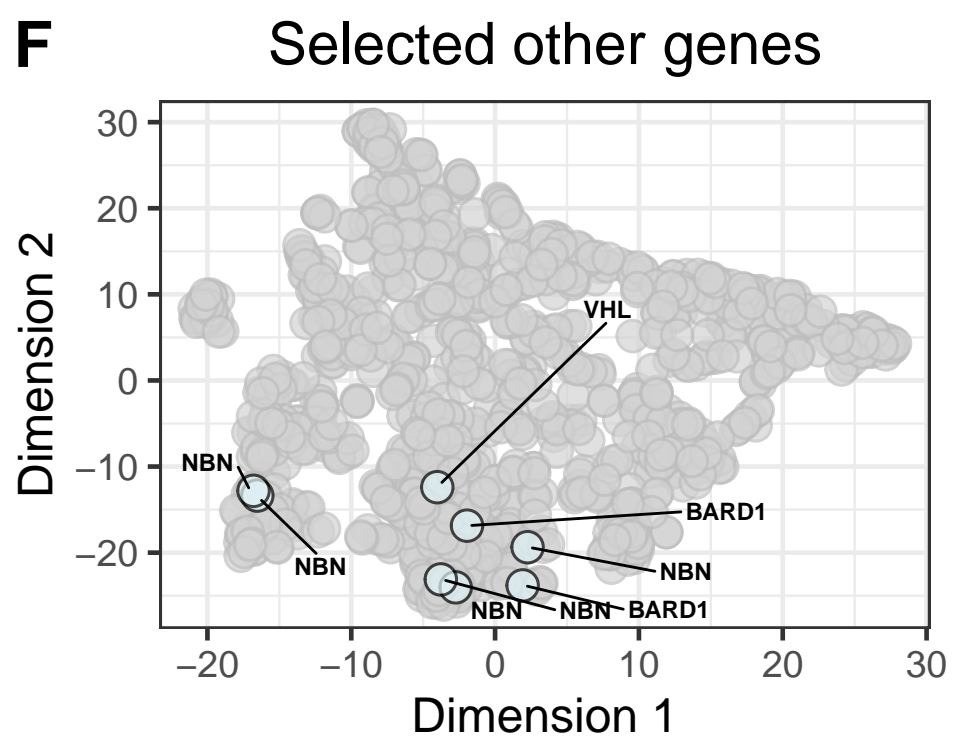

Supplement: S30 Fig — Using the same two-dimensional representation of mutational signatures shown in S10 Fig, this plot indicates which patients had somatic mutations in non-BRCA cancer-predisposition genes. Diamond shapes indicate patients for whom no loss-of-heterozygosity was observed. (PDF) [file pone.0239197.s030.pdf]

# Copy number alterations

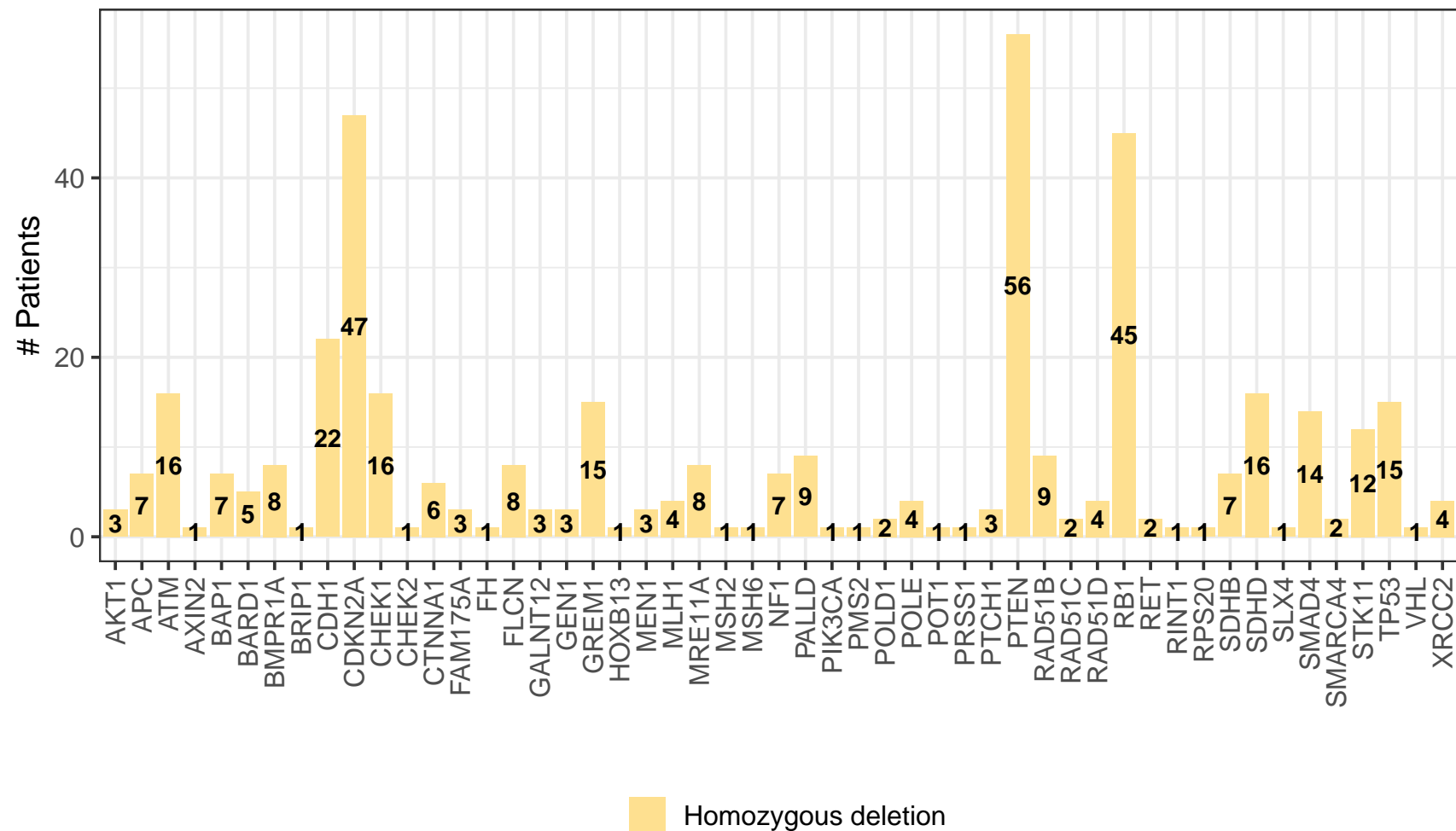

Supplement: S31 Fig — This graph omits genes in which we observed no homozygous deletions. (PDF) [file pone.0239197.s031.pdf]

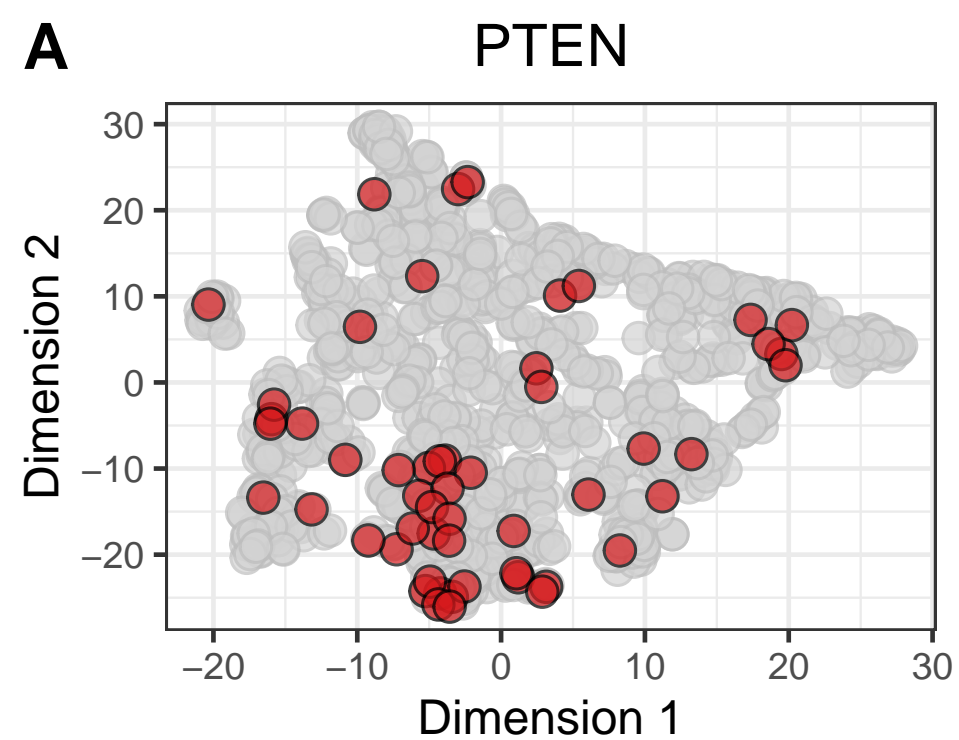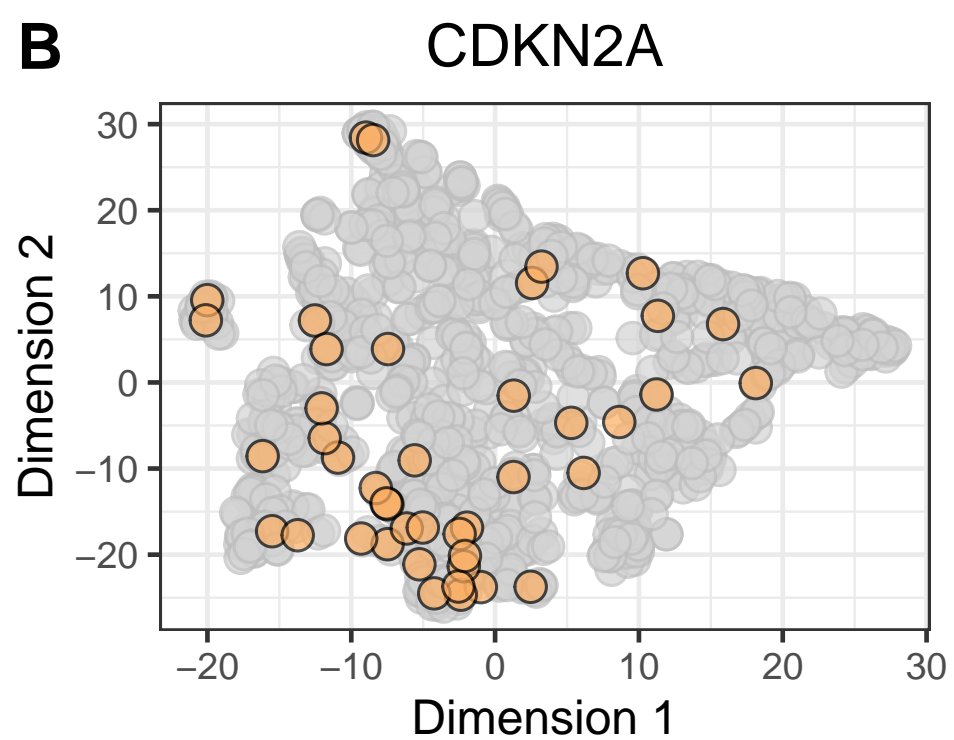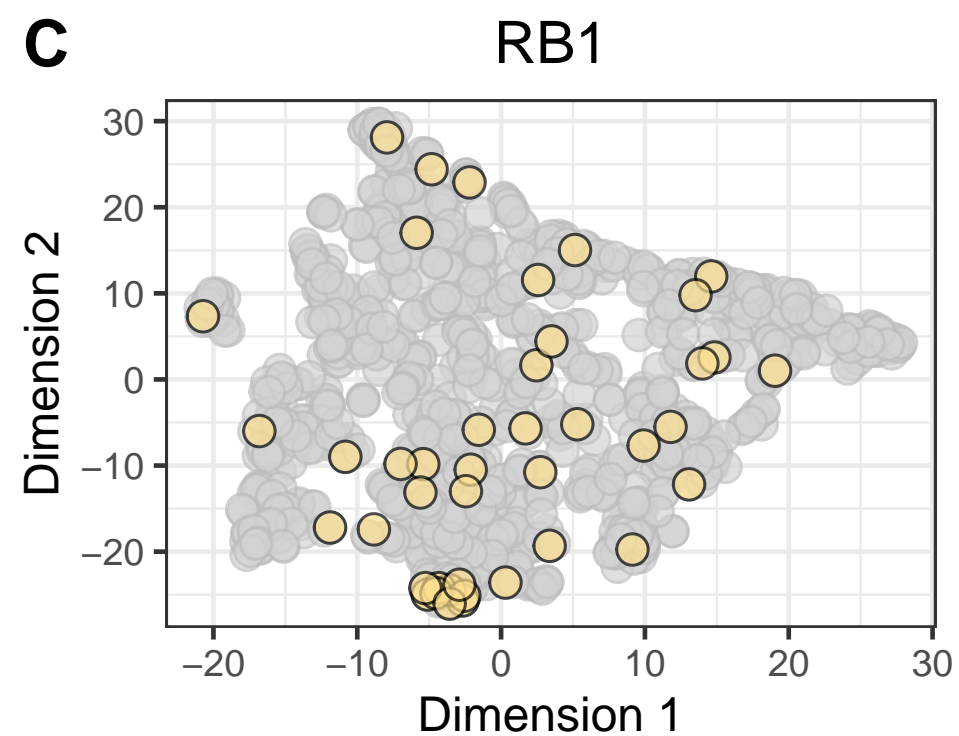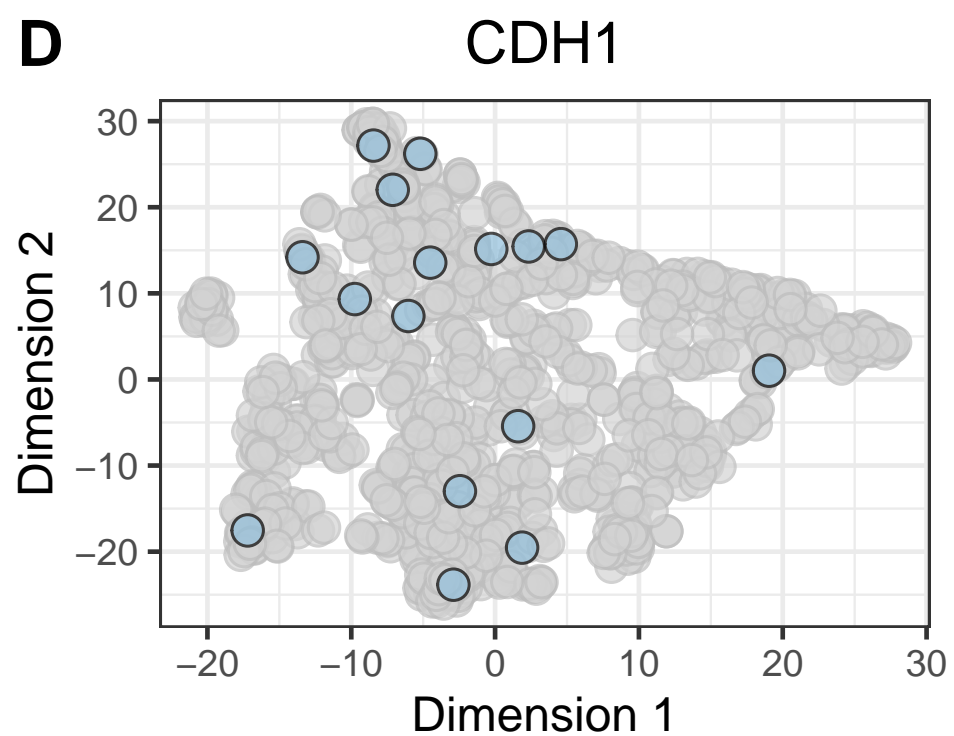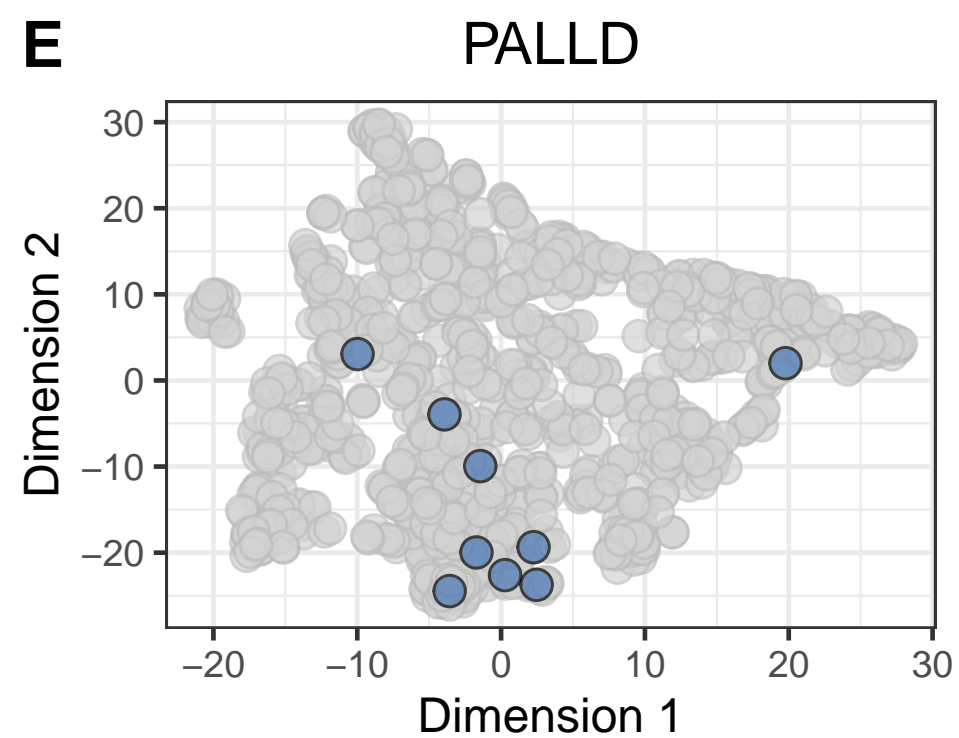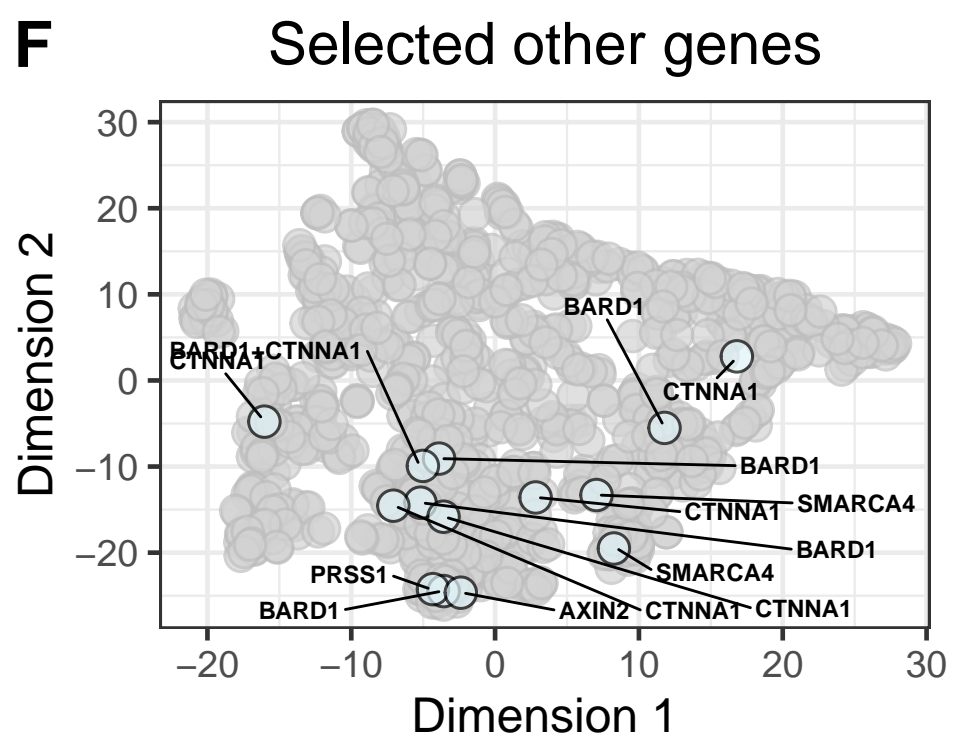

Supplement: S32 Fig — Using the same two-dimensional representation of mutational signatures shown in S10 Fig, this plot indicates which patients had homozygous deletions in non-BRCA cancer-predisposition genes. Diamond shapes indicate patients for whom no loss-of-heterozygosity was observed. (PDF) [file pone.0239197.s032.pdf]

# DNA methylation

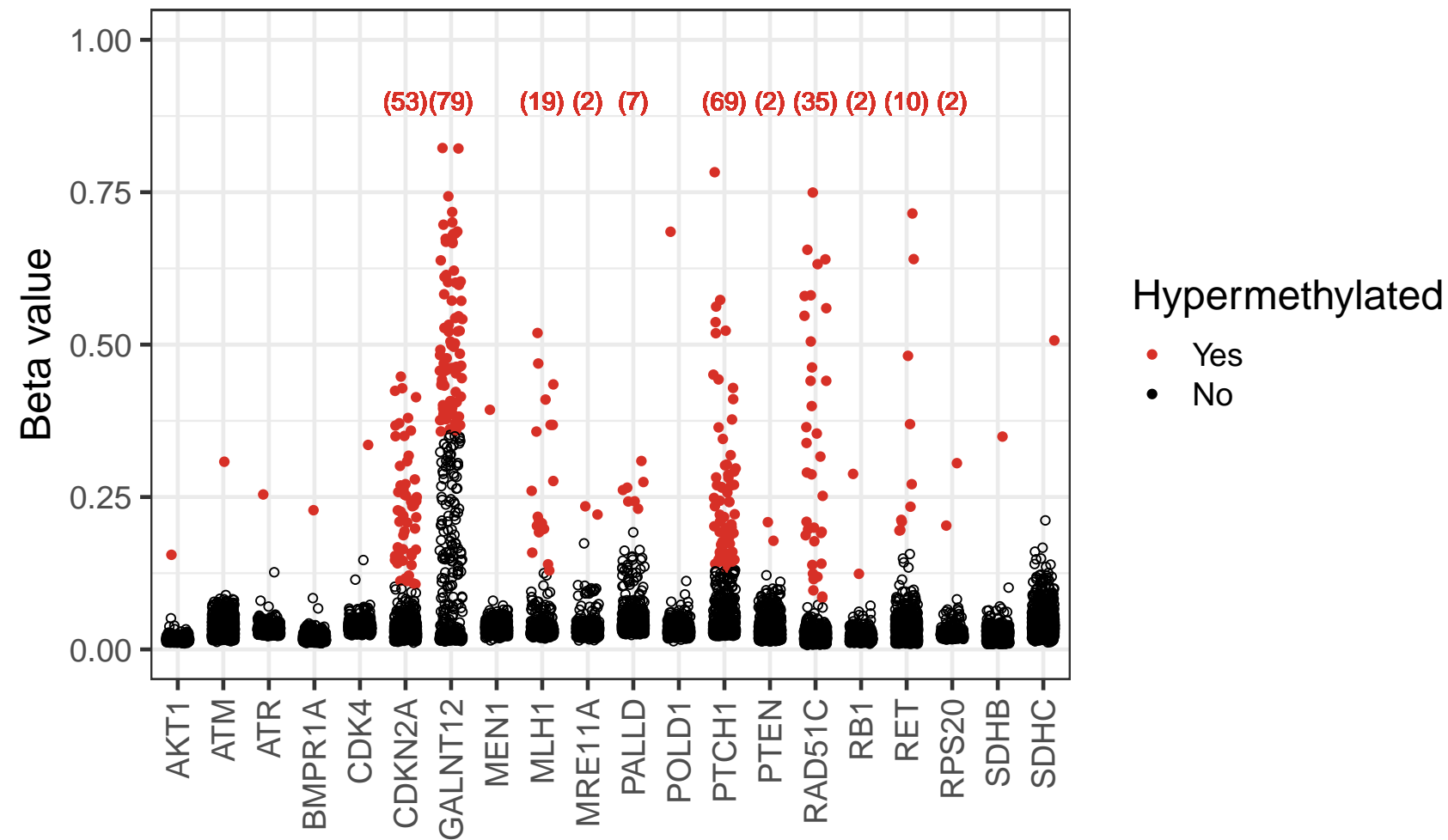

Supplement: S33 Fig — Tumors that we classified as having hypermethylation events are highlighted as red points. This graph omits genes in which we observed no hypermethylation events. (PDF) [file pone.0239197.s033.pdf]

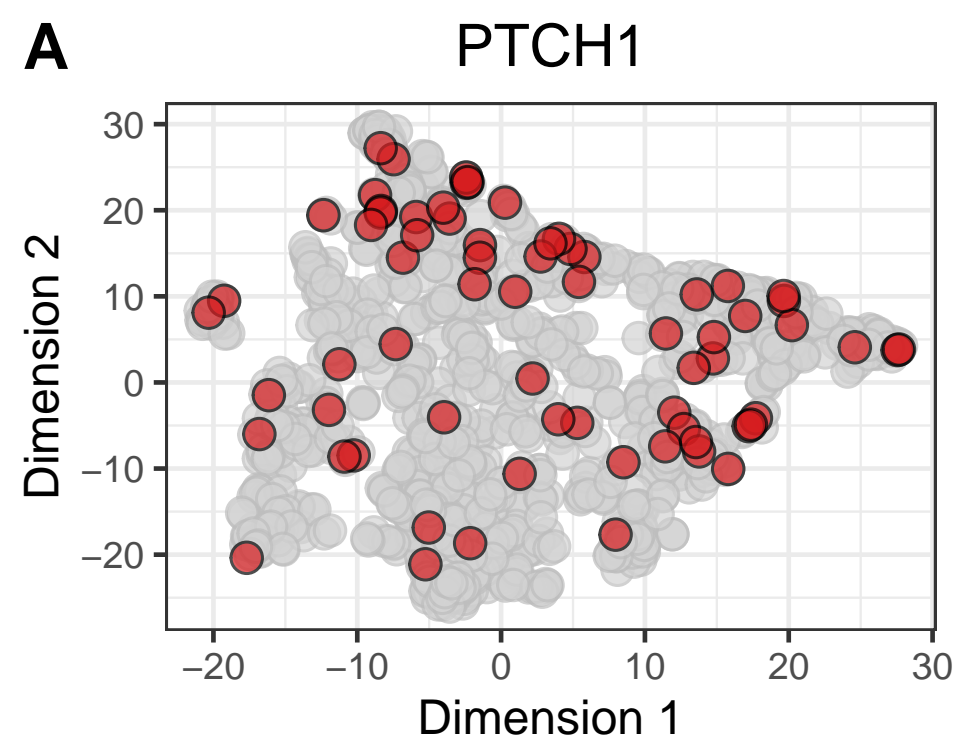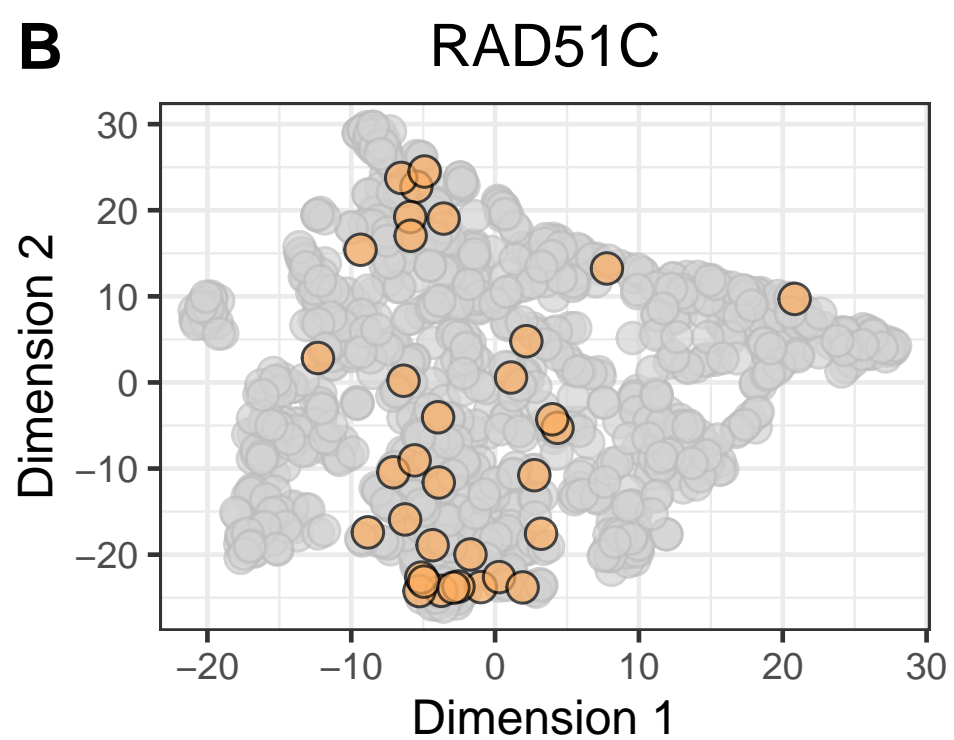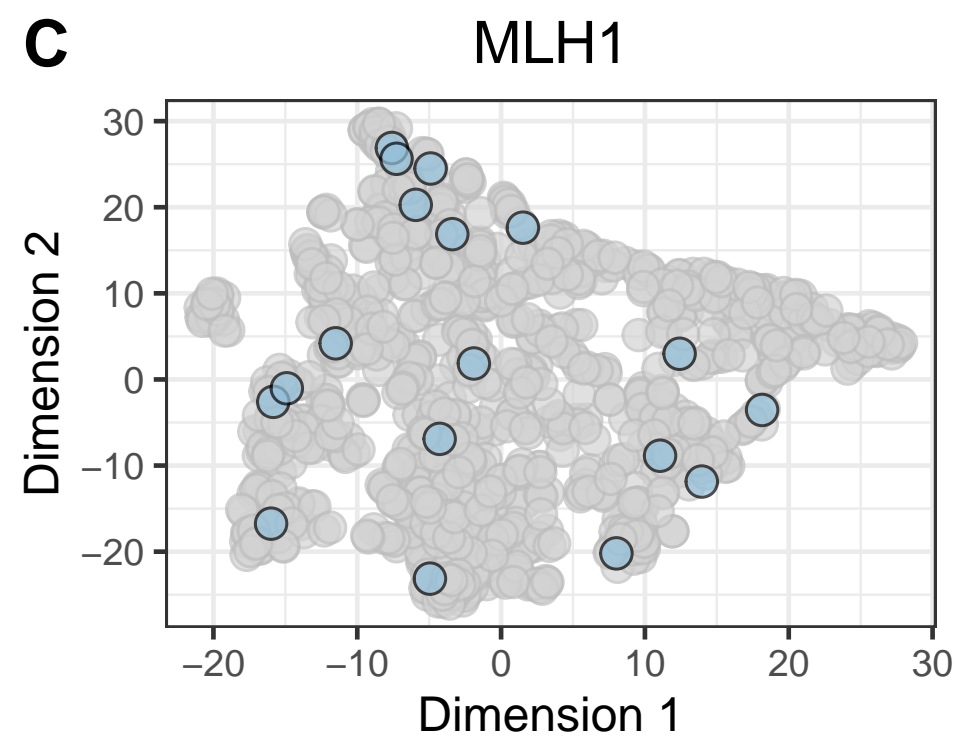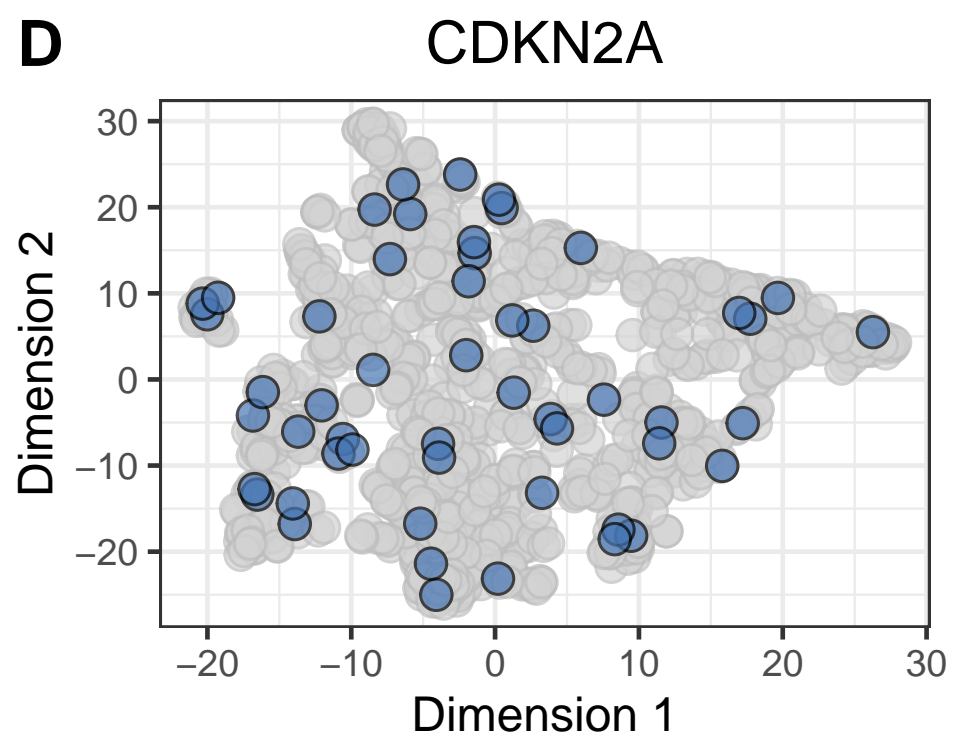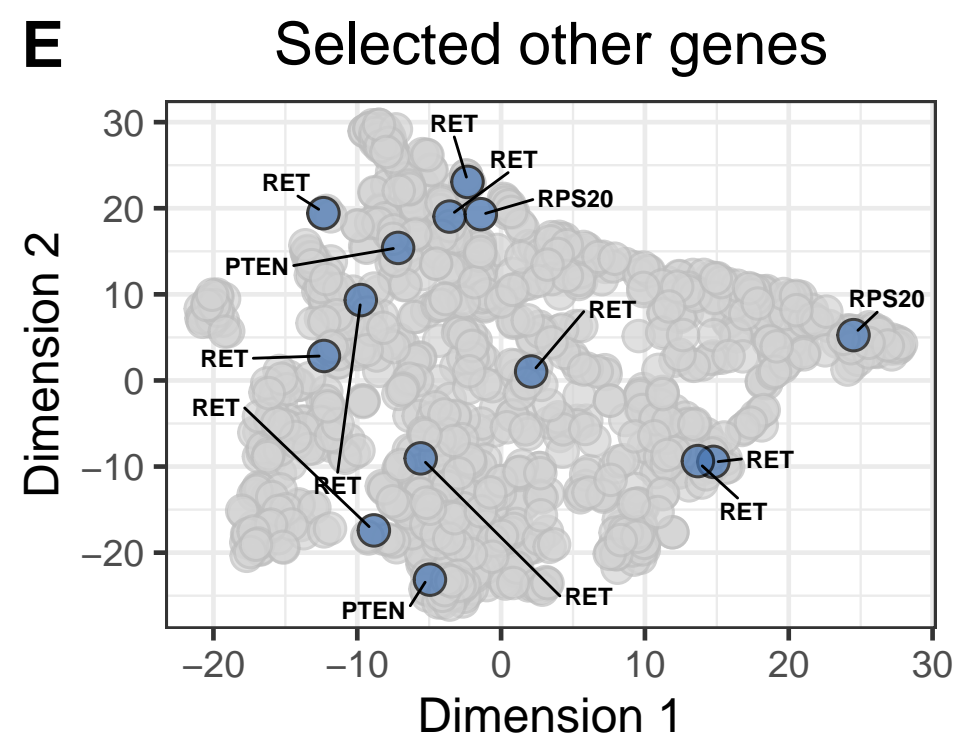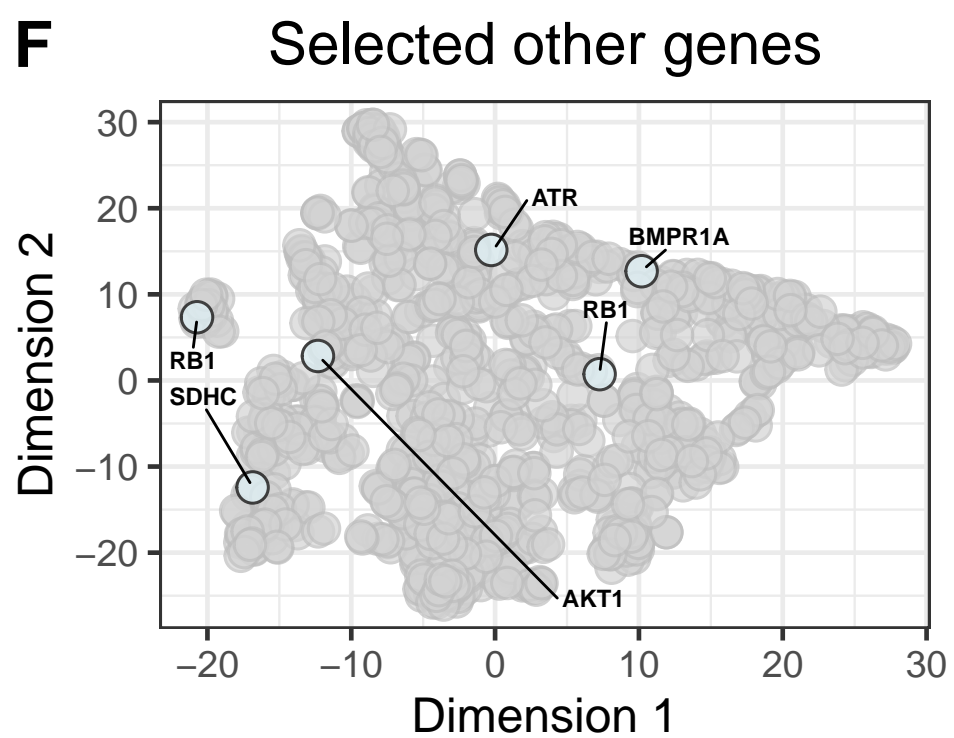

Supplement: S34 Fig — Using the same two-dimensional representation of mutational signatures shown in S10 Fig, this plot indicates which patients had hypermethylation events in non-BRCA cancer-predisposition genes. Diamond shapes indicate patients for whom no loss-of-heterozygosity was observed. (PDF) [file pone.0239197.s034.pdf]

# Low mRNA expression

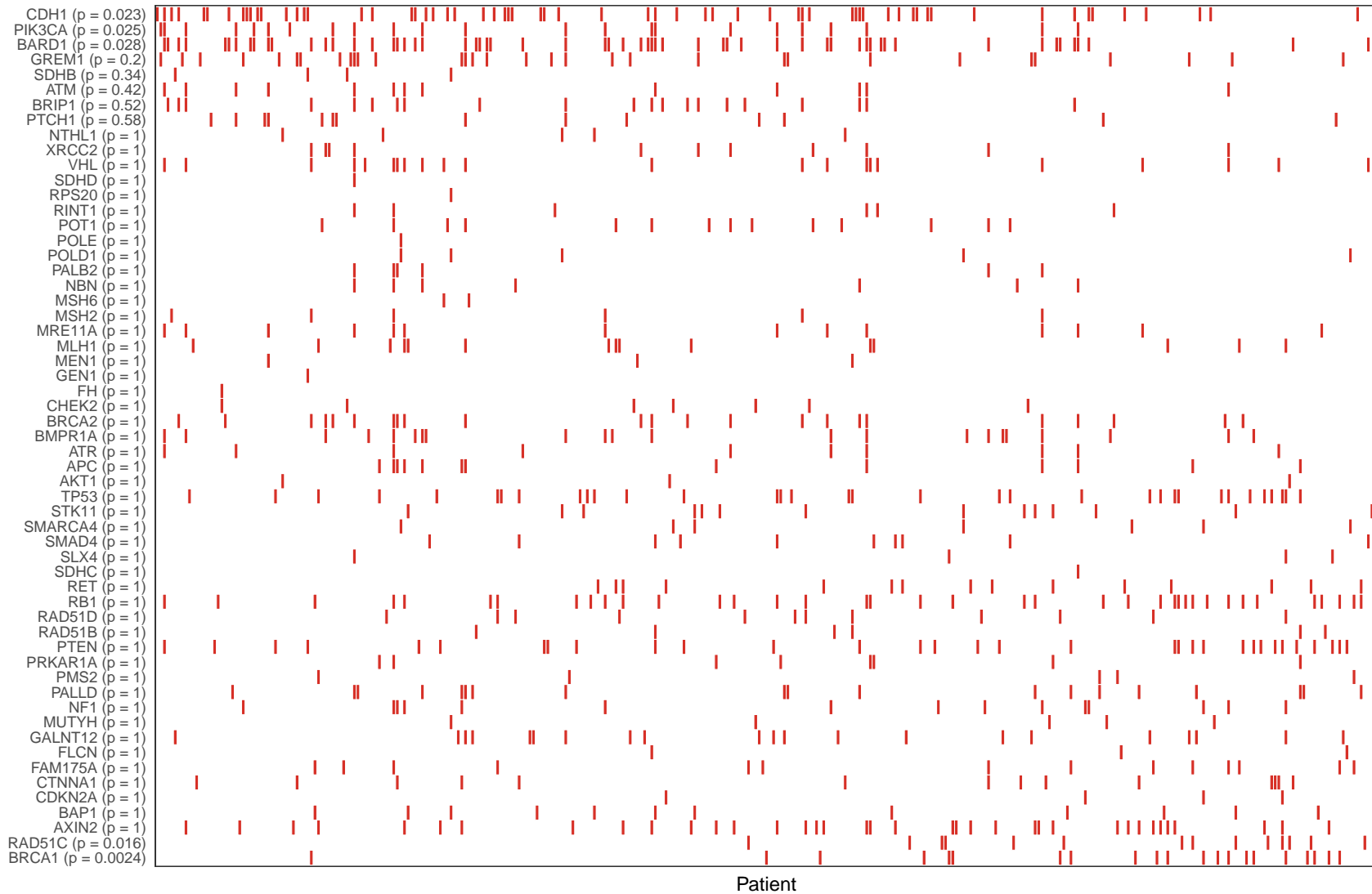

Supplement: S36 Fig — We identified tumors with low expression for cancer-predisposition genes (see S35 Fig) and evaluated whether the somatic-mutation signatures of these tumors were relatively similar or dissimilar to the BRCA reference group. Each red rectangle represents a patient sample that expressed a given gene at low levels. Low expression of RAD51C and BRCA1 showed the strongest positive correlation between gene-expression status and the BRCAness reference group. Low expression of BARD1 and CDH1 showed the strongest negative correlation between gene-expression status and the BRCAness reference group. Genes for which no tumors exhibited low expression are omitted. (PDF) [file pone.0239197.s036.pdf]

# Drug response

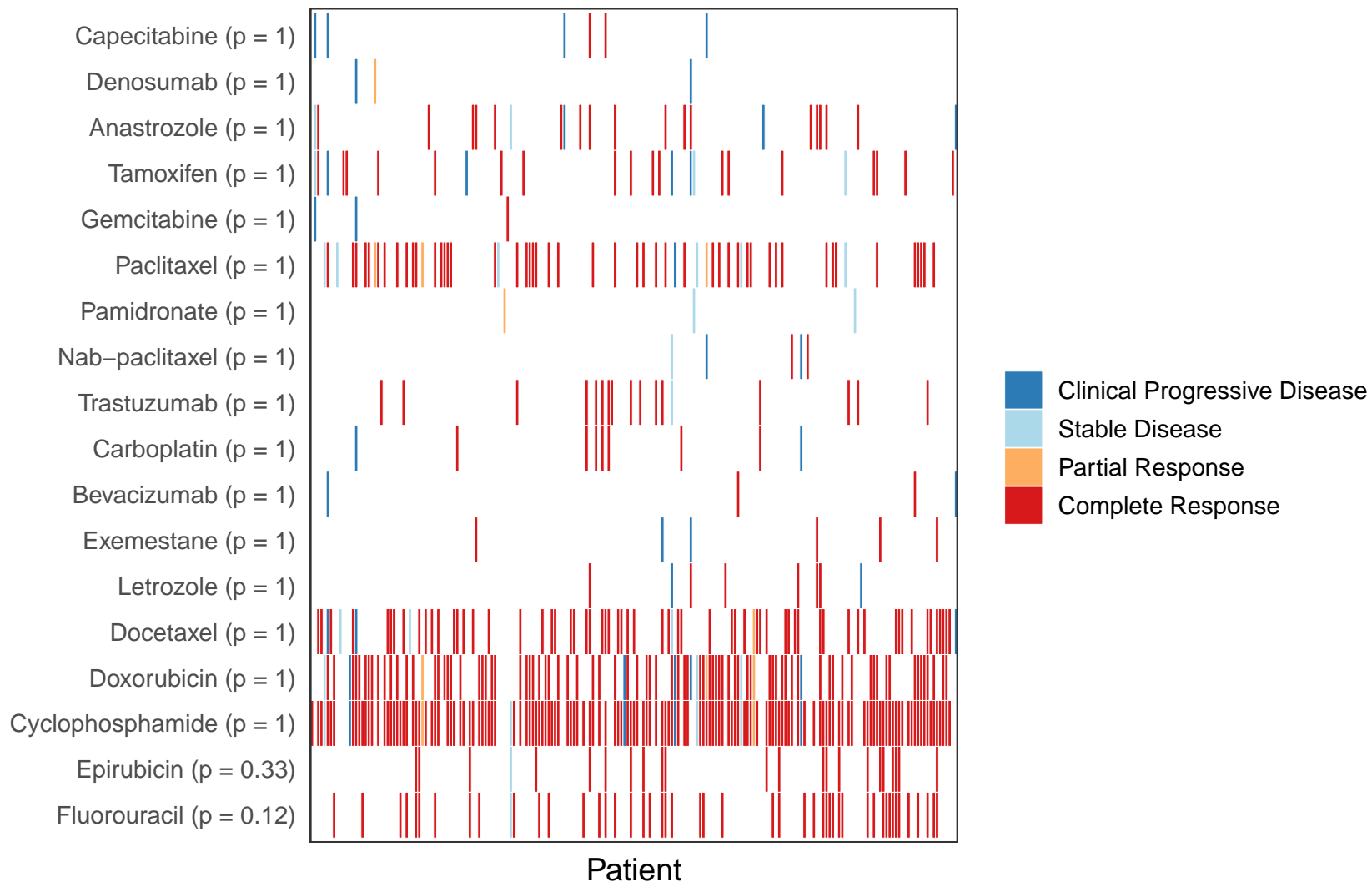

Supplement: S38 Fig — We evaluated clinical treatment responses for 211 TCGA patients for whom drug-response data were available. Responses for none of the drugs were significantly correlated with BRCA aberration status based on somatic-mutation signatures. (PDF) [file pone.0239197.s038.pdf]
