# Supplementary material for: Effects of germline and somatic events in candidate BRCA-like genes on breast-tumor signatures
Source: PLoS One. 2020 Sep 30;15(9):e0239197. doi: 10.1371/journal.pone.0239197 (PMC7526916; doi:10.1371/journal.pone.0239197)
Supplement: S21 Fig — Using the same two-dimensional representation of gene-expression profiles shown in Fig 2, this plot indicates which patients had germline mutations (A, B), somatic mutations (C, D), homozygous deletions (E, F), or hypermethylation events (G, H) in BRCA1 and BRCA2, respectively. Many of these tumors overlapped with the Basal-like subtype, but other tumors were dispersed broadly across the gene-expression landscape. Diamond shapes indicate patients for whom no loss-of-heterozygosity was observed. Data are shown for all patients, even those for whom we did not have all types of data. (PDF) [file pone.0239197.s021.pdf]

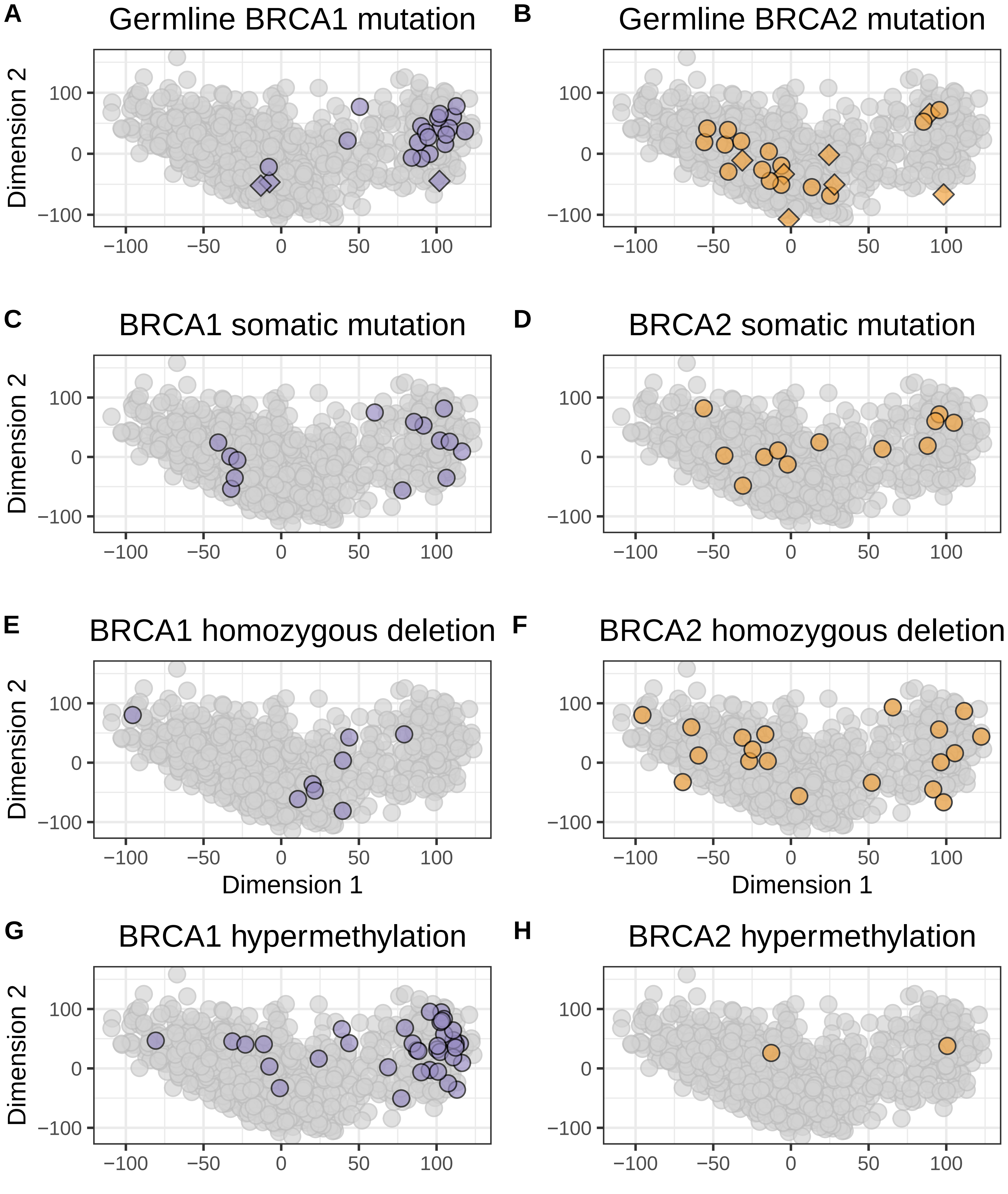
A
Germline BRCA1 mutation
B
Germline BRCA2 mutation
C
BRCA1 somatic mutation
D
BRCA2 somatic mutation
E
BRCA1 homozygous deletion
F
BRCA2 homozygous deletion
G
BRCA1 hypermethylation
H
BRCA2 hypermethylation
Dimension 1
Dimension 2
−100
−50
0
50
100
