# Supplementary material for: Effects of germline and somatic events in candidate BRCA-like genes on breast-tumor signatures
Source: PLoS One. 2020 Sep 30;15(9):e0239197. doi: 10.1371/journal.pone.0239197 (PMC7526916; doi:10.1371/journal.pone.0239197)
Supplement: S37 Fig — Red rectangles indicate patients that were positive for each respective clinical characteristic. Tumors with triple-negative hormone receptors, infiltrating ductal carcinoma histologies, or close surgical margins overlapped most with BRCA-aberrant tumors based on somatic-mutation signatures. (PDF) [file pone.0239197.s037.pdf]

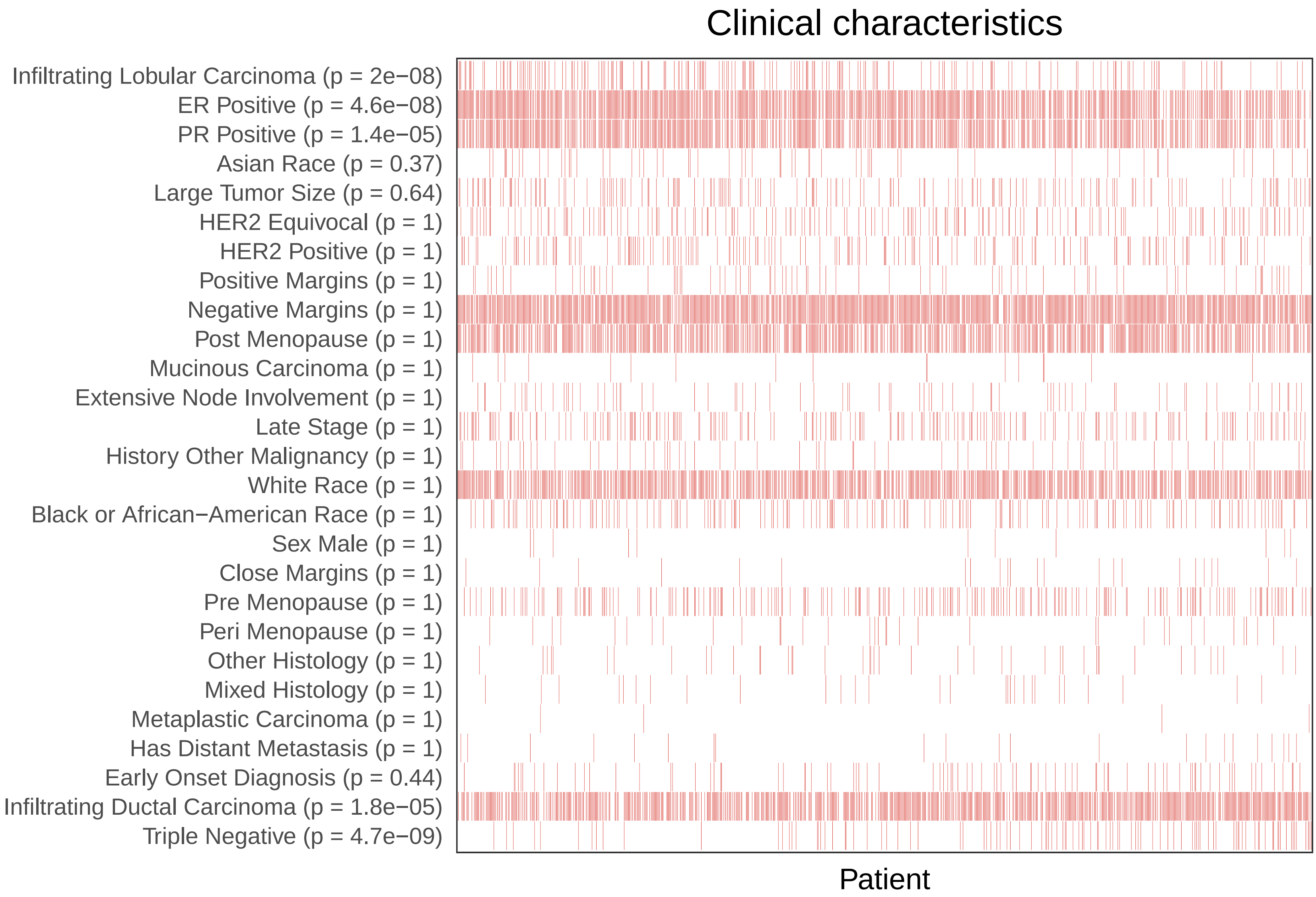
Clinical characteristics
Infiltrating Lobular Carcinoma (p = 2e−08)
ER Positive (p = 4.6e−08)
PR Positive (p = 1.4e−05)
Asian Race (p = 0.37)
Large Tumor Size (p = 0.64)
HER2 Equivocal (p = 1)
HER2 Positive (p = 1)
Positive Margins (p = 1)
Negative Margins (p = 1)
Post Menopause (p = 1)
Mucinous Carcinoma (p = 1)
Extensive Node Involvement (p = 1)
Late Stage (p = 1)
History Other Malignancy (p = 1)
White Race (p = 1)
Black or African−American Race (p = 1)
Sex Male (p = 1)
Close Margins (p = 1)
Pre Menopause (p = 1)
Peri Menopause (p = 1)
Other Histology (p = 1)
Mixed Histology (p = 1)
Metaplastic Carcinoma (p = 1)
Has Distant Metastasis (p = 1)
Early Onset Diagnosis (p = 0.44)
Infiltrating Ductal Carcinoma (p = 1.8e−05)
Triple Negative (p = 4.7e−09)
Patient
