# Supplementary material for: Effects of germline and somatic events in candidate BRCA-like genes on breast-tumor signatures
Source: PLoS One. 2020 Sep 30;15(9):e0239197. doi: 10.1371/journal.pone.0239197 (PMC7526916; doi:10.1371/journal.pone.0239197)
Supplement: S1 Table — We evaluated somatic mutations in BRCA1 and BRCA2 based on variant type, predicted effects on protein sequence, evolutionary conservation, minor allele frequency, evidence in ClinVar, etc. This table provides information about each variant and specified criteria that we considered. A value of 1 in the Pathogenicity column indicates that we considered the variant to be pathogenic in our analyses. (DOCX) [file pone.0239197.s039.docx]

| **Sample** | **Chr** | **Position** | **Gene** | **Type** | **Notes** | **Classification** | **Pathogenicity** |
| --- | --- | --- | --- | --- | --- | --- | --- |
| TCGA-AN-A0AL | 13 | 32332280 | BRCA2 | Deletion | 25bp fs insertion exon 10 | Pathogenic | 1 |
| TCGA-A7-A26F | 13 | 32332830 | BRCA2 | Insertion | in frame insertion exon 10 | Likely benign | 0 |
| TCGA-A8-A07R | 13 | 32332961 | BRCA2 | SNV | Non-conserved SNV exon 10 | Likely benign | 0 |
| TCGA-E2-A14W | 13 | 32333271 | BRCA2 | Deletion | 5p fs insertion | Pathogenic | 1 |
| TCGA-BH-A0HF | 13 | 32336991 | BRCA2 | SNV | exon 11 SNV - not conserved | Likely benign | 0 |
| TCGA-AC-A23H | 13 | 32337452 | BRCA2 | SNV | conserved missensse exon 11 | VUS | 0 |
| TCGA-A8-A07I | 13 | 32338418 | BRCA2 | SNV | Non-conserved SNV exon 11 | Likely benign | 0 |
| TCGA-A8-A07R | 13 | 32338762 | BRCA2 | Deletion | fs deletion | Pathogenic | 1 |
| TCGA-BH-A0HB | 13 | 32339399 | BRCA2 | SNV | Non-conserved SNV exon 11 | Likely benign | 0 |
| TCGA-D8-A1JP | 13 | 32340247 | BRCA2 | Deletion | fs deletion | Pathogenic | 1 |
| TCGA-A8-A07J | 13 | 32340266 | BRCA2 | Insertion | fs insertion | Pathogenic | 1 |
| TCGA-BH-A0HQ | 13 | 32340346 | BRCA2 | SNV | Non-conserved SNV exon 11 | Likely benign | 0 |
| TCGA-E9-A1R2 | 13 | 32340800 | BRCA2 | Deletion | fs deletion | Pathogenic | 1 |
| TCGA-D8-A27G | 13 | 32340878 | BRCA2 | SNV | Non-conserved SNV exon 11 | Likely benign | 0 |
| TCGA-AR-A0U0 | 13 | 32354938 | BRCA2 | Insertion | fs insertion | Pathogenic | 1 |
| TCGA-A8-A08L | 13 | 32357930 | BRCA2 | SNV | exon 16 donor site, rs81002809 | Likely pathogenic per splice prediction | 1 |
| TCGA-AN-A0AT | 13 | 32362665 | BRCA2 | SNV | exon 17 conserved missense | VUS | 1 |
| TCGA-A2-A0T0 | 13 | 32379339 | BRCA2 | SNV | exon 22 moderate conservation, lack of conservation | VUS | 0 |
| TCGA-S3-AA17 | 13 | 32394700 | BRCA2 | Deletion | in frame deletion, non-conserved, exon 25 | Probably benign, but classify as VUS | 1 |
| TCGA-AC-A2QH | 13 | 32394859 | BRCA2 | Insertion | in frame insertion, exon 25 | Probably benign, but classify as VUS | 1 |
| TCGA-A8-A0A7 | 13 | 32396919 | BRCA2 | SNV | exon 26 missense not highly conserved | Likely benign | 0 |
| TCGA-C8-A12T | 13 | 32396925 | BRCA2 | SNV | exon 26 missense not highly conserved | Likely benign | 0 |
| TCGA-AO-A124 | 13 | 32398424 | BRCA2 | SNV | exon 27 missense not highly conserved | Likely benign | 0 |
| TCGA-A8-A08T | 13 | 32398507 | BRCA2 | Insertion | exon 27 late frameshift insertion | Likely pathogenic | 1 |
| TCGA-AN-A046 | 13 | 32398537 | BRCA2 | SNV | exon 27 missense, conserved region, LB (after polymorphic stop @ 3326) | VUS | 0 |
| TCGA-BH-A0DS | 17 | 43047678 | BRCA1 | SNV | exon 23, Q or E moderate conservation | VUS | 1 |
| TCGA-AN-A0XU | 17 | 43049164 | BRCA1 | SNV | exon 23, moderate conservation | VUS | 1 |
| TCGA-AN-A041 | 17 | 43051116 | BRCA1 | Insertion | exon 21 near splice acceptor, Frameshift insertion, pathogenic | Pathogenic | 1 |
| TCGA-AR-A1AO | 17 | 43051118 | BRCA1 | SNV | exon 21 acceptor site -1, likely pathogenic | Likely pathogenic | 1 |
| TCGA-A7-A13D | 17 | 43057078 | BRCA1 | SNV | exon 20 NM_007294.3(BRCA1):c.5251C>T (p.Arg1751Ter) pathogenic see ClinVar | Pathogenic | 1 |
| TCGA-D8-A27M | 17 | 43071072 | BRCA1 | Deletion | frameshift deletion, pathogenic | Pathogenic | 1 |
| TCGA-BH-A2L8 | 17 | 43071182 | BRCA1 | SNV | exon 16, not conserved, likely benign | Likely benign | 0 |
| TCGA-BH-A0WA | 17 | 43071239 | BRCA1 | SNV | exon 6 acceptor site, NM_007294.3(BRCA1):c.4676-1G>A, Pathogenic | Pathogenic | 1 |
| TCGA-PE-A5DE | 17 | 43082506 | BRCA1 | SNV | exon 12 not conserved, other beingn variant at same site, likely benign | Likely benign | 0 |
| TCGA-LL-A8F5 | 17 | 43082539 | BRCA1 | SNV | exon 12, conservation moderate, likely benign | Likely benign | 0 |
| TCGA-BH-A0HF | 17 | 43091028 | BRCA1 | SNV | exon 11 SNV - not conserved | Likely benign | 0 |
| TCGA-C8-A12T | 17 | 43091501 | BRCA1 | SNV | Non-conserved SNV exon 10 | Likely benign | 0 |
| TCGA-A7-A13G | 17 | 43091619 | BRCA1 | Insertion | fs exon 10 | Pathogenic | 1 |
| TCGA-AN-A046 | 17 | 43092239 | BRCA1 | SNV | Non-conserved SNV exon 10 | Likely benign | 0 |
| TCGA-E2-A1L9 | 17 | 43092451 | BRCA1 | SNV | Non-conserved SNV exon 10 | Likely benign | 0 |
| TCGA-A1-A0SH | 17 | 43092731 | BRCA1 | SNV | Non-conserved SNV exon 10 | Likely benign | 0 |
| TCGA-GM-A2D9 | 17 | 43092787 | BRCA1 | SNV | Non-conserved SNV exon 10 | Likely benign | 0 |
| TCGA-A7-A6VW | 17 | 43092821 | BRCA1 | Deletion | fs exon 10 | Pathogenic | 1 |
| TCGA-A2-A25B | 17 | 43093373 | BRCA1 | SNV | Non-conserved SNV exon 10 | Likely benign | 0 |
| TCGA-A7-A26H | 17 | 43093466 | BRCA1 | Deletion | fs exon 10 | Pathogenic | 1 |
| TCGA-A8-A06X | 17 | 43094717 | BRCA1 | SNV | Non-conserved SNV exon 10 | Likely benign | 0 |
| TCGA-A8-A08G | 17 | 43094800 | BRCA1 | Insertion | fs insertion exon 10 | Pathogenic | 1 |
| TCGA-A8-A08G | 17 | 43094802 | BRCA1 | Insertion | nonframeshift insertion, exon 10 | Likely benign | 0 |
| TCGA-E9-A1NC | 17 | 43104148 | BRCA1 | SNV | nonframeshift insertion, exon 10 | Likely benign | 0 |
| TCGA-AO-A0J4 | 17 | 43104922 | BRCA1 | SNV | exon 5 missense, V>F | VUS | 1 |
| TCGA-A2-A4S0 | 17 | 43106519 | BRCA1 | SNV | exon 4 missense, VUS, functional in Findlay assay | VUS | 0 |
| TCGA-A1-A0SO | 17 | 43124016 | BRCA1 | SNV | exon 2 +1 donor site | Pathogenic | 1 |
| TCGA-B6-A0X1 | 17 | 43124029 | BRCA1 | Deletion | exon 2 frameshift | Pathogenic | 1 |
| TCGA-A1-A0SI | 17 | 43124072 | BRCA1 | SNV | exon 2 missense, moderate conservation, functional in Findlay assay | VUS | 0 |
| TCGA-E2-A1BD | 17 | 43124091 | BRCA1 | SNV | exon 1, missense | Likely benign | 0 |
