# Supplementary material for: Effects of germline and somatic events in candidate BRCA-like genes on breast-tumor signatures
Source: PLoS One. 2020 Sep 30;15(9):e0239197. doi: 10.1371/journal.pone.0239197 (PMC7526916; doi:10.1371/journal.pone.0239197)
Supplement: S2 Table — Via cross validation, we used gene-expression profiles and somatic-mutation signatures, respectively, to predict whether a given patient/tumor harbored particular types of aberrations. Sensitivity is equivalent to the true-positive rate. Specificity is equivalent to the true-negative rate. The area under the receiver operator characteristic curve (AUROC) quantifies the balance between sensitivity and specificity across a range of prediction thresholds. (DOCX) [file pone.0239197.s040.docx]

| **Aberration Type** | **Data Type** | **Sensitivity** | **Specificity** | **AUROC** |
| --- | --- | --- | --- | --- |
| BRCA1 germline mutation | Gene Expression | 0.80 | 0.77 | 0.86 |
| BRCA1 germline mutation | Mutational Signatures | 0.71 | 0.82 | 0.83 |
| BRCA2 germline mutation | Gene Expression | 0.62 | 0.42 | 0.57 |
| BRCA2 germline mutation | Mutational Signatures | 0.67 | 0.78 | 0.75 |
| BRCA1 somatic mutation | Gene Expression | 0.76 | 0.70 | 0.73 |
| BRCA1 somatic mutation | Mutational Signatures | 0.70 | 0.81 | 0.81 |
| BRCA2 somatic mutation | Gene Expression | 0.71 | 0.50 | 0.62 |
| BRCA2 somatic mutation | Mutational Signatures | 0.77 | 0.81 | 0.88 |
| BRCA1 homozygous deletion | Gene Expression | 0.72 | 0.0 | 0.29 |
| BRCA1 homozygous deletion | Mutational Signatures | 0.71 | 0.63 | 0.68 |
| BRCA2 homozygous deletion | Gene Expression | 0.74 | 0.35 | 0.52 |
| BRCA2 homozygous deletion | Mutational Signatures | 0.75 | 0.72 | 0.80 |
| BRCA1 hypermethylation | Gene Expression | 0.81 | 0.81 | 0.87 |
| BRCA1 hypermethylation | Mutational Signatures | 0.78 | 0.82 | 0.85 |
| BRCA2 hypermethylation | Gene Expression | 0.74 | 0.0 | 0.52 |
| BRCA2 hypermethylation | Mutational Signatures | 0.96 | 0.0 | 0.94 |
