# Supplementary material for: Effects of germline and somatic events in candidate BRCA-like genes on breast-tumor signatures
Source: PLoS One. 2020 Sep 30;15(9):e0239197. doi: 10.1371/journal.pone.0239197 (PMC7526916; doi:10.1371/journal.pone.0239197)
Supplement: S14 Fig — This graph indicates how many patients had each type of molecular aberration and the level of overlap among these aberrations within a given patient. In most cases, these aberrations were mutually exclusive from each other; however, some overlap did occur. For example, one patient had a somatic mutation in BRCA1 and hypermethylation of the same gene. This graph only depicts patients for whom all four types of molecular data were available. (PDF) [file pone.0239197.s014.pdf]

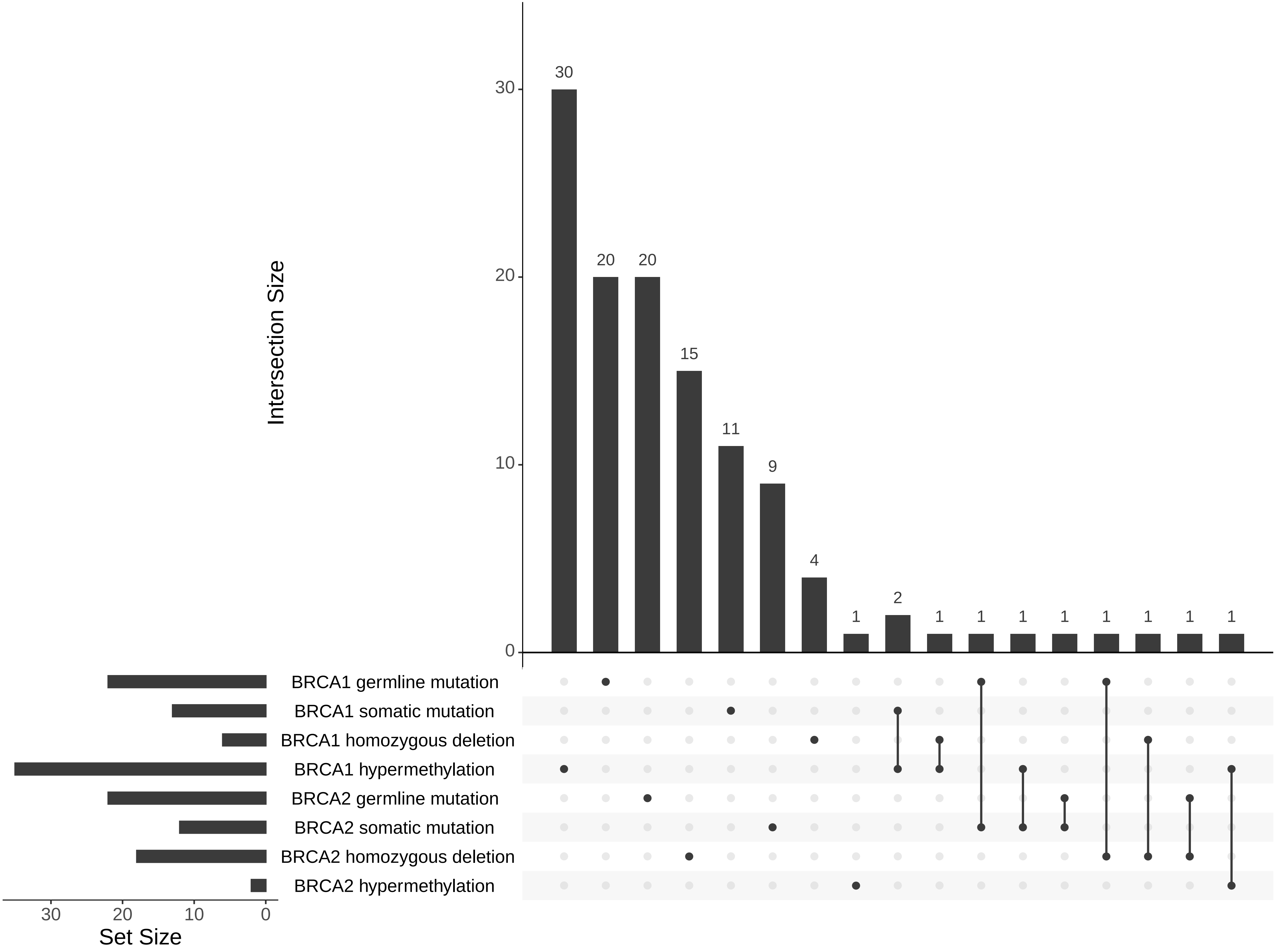

Intersection Size
30
20
10
0
30
20
20
15
11
9
4
1
2
1
1
1
1
1
1
1
1
BRCA1 germline mutation
BRCA1 somatic mutation
BRCA1 homozygous deletion
BRCA1 hypermethylation
BRCA2 germline mutation
BRCA2 somatic mutation
BRCA2 homozygous deletion
BRCA2 hypermethylation
30
20
10
0
Set Size
