# Supplementary material for: Effects of germline and somatic events in candidate BRCA-like genes on breast-tumor signatures
Source: PLoS One. 2020 Sep 30;15(9):e0239197. doi: 10.1371/journal.pone.0239197 (PMC7526916; doi:10.1371/journal.pone.0239197)
Supplement: S35 Fig — For each gene, we identified tumors that expressed these genes at relatively low levels compared to other breast tumors; these low expressors are highlighted as red points. (PDF) [file pone.0239197.s035.pdf]

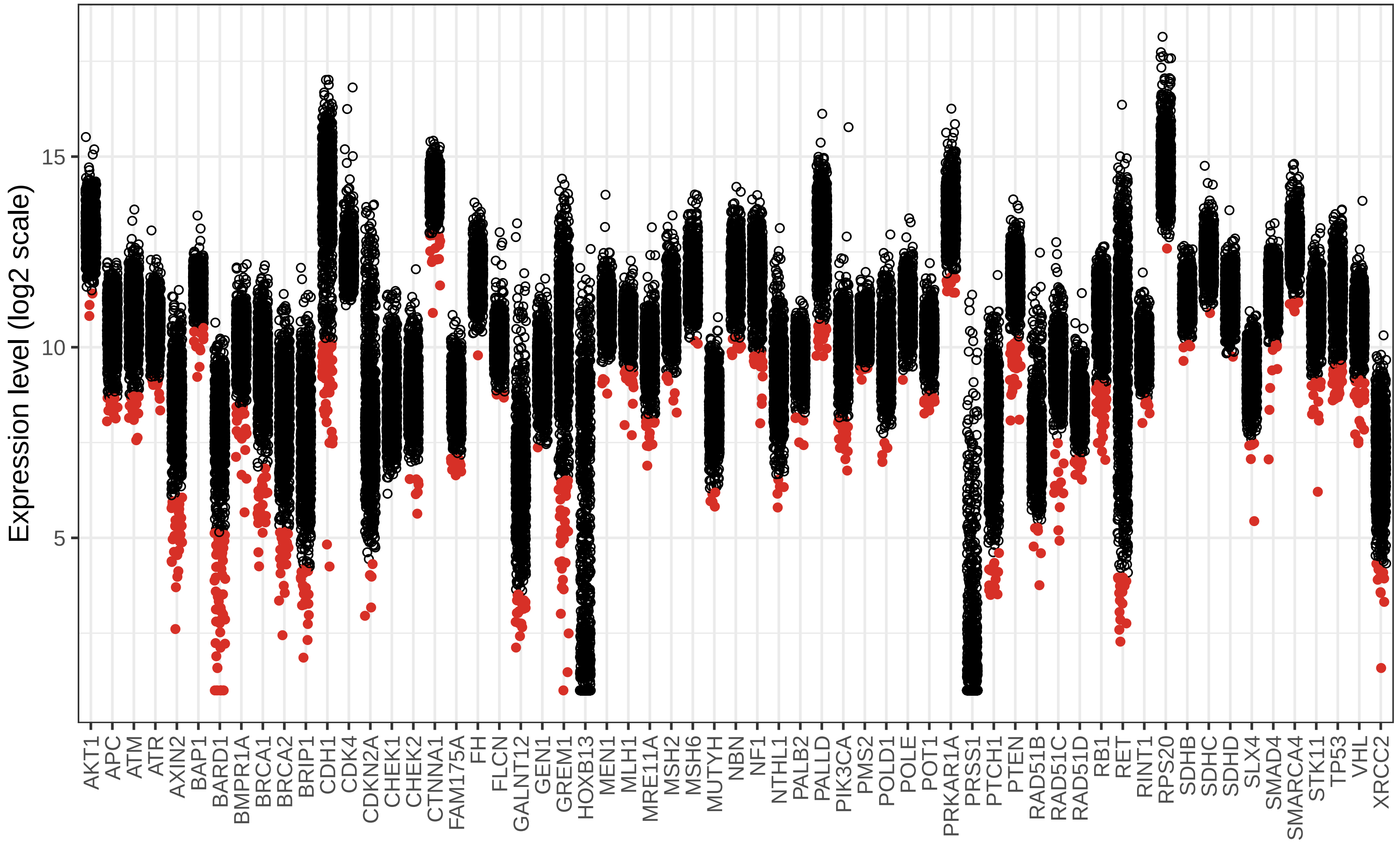

Expression level (log2 scale)
15
10
5
AKT1
APC
ATM
ATR
AXIN2
BAP1
BARD1
BMPR1A
BRCA1
BRCA2
BRIP1
CDH1
CDK4
CDKN2A
CHEK1
CHEK2
CTNNA1
FAM175A
FH
FLCN
GALNT12
GEN1
GREM1
HOXB13
MEN1
MLH1
MRE11A
MSH2
MSH6
MUTYH
NBN
NF1
NTHL1
PALB2
PALLD
PIK3CA
PMS2
POLD1
POLE
POT1
PRKAR1A
PRSS1
PTCH1
PTEN
RAD51B
RAD51C
RAD51D
RB1
RET
RINT1
RPS20
SDHB
SDHC
SDHD
SLX4
SMAD4
SMARCA4
STK11
TP53
VHL
XRCC2
