# Supplementary material for: Effects of germline and somatic events in candidate BRCA-like genes on breast-tumor signatures
Source: PLoS One. 2020 Sep 30;15(9):e0239197. doi: 10.1371/journal.pone.0239197 (PMC7526916; doi:10.1371/journal.pone.0239197)
Supplement: Attachment — Submitted filename: Response_to_Reviewers.pdf [file pone.0239197.s041.pdf]

DEPARTMENT OF BIOLOGY

Dear editors:

Thank you for reviewing our manuscript, "Markers of BRCAness in breast cancer." We apologize for the technical difficulties with the supplementary section of our manuscript. We have provided the supplementary tables and figures as separate files to avoid further complications. Below we provide a detailed response to the editor's and reviewers' comments.

Warm regards,

Stephen R. Piccolo, PhD
Assistant Professor
Department of Biology
Brigham Young University
(801) 422-7116
Stephen_Piccolo@byu.edu

We have listed the editor's and reviewers' comments in gray text below. Our comments are black text.

1. Please ensure that your manuscript meets PLOS ONE's style requirements, including those for file naming. The PLOS ONE style templates can be found at https://journals.plos.org/plosone/s/file?id=wjVg/PLOSOne_formatting_sample_main_body.pdf and https://journals.plos.org/plosone/s/file?id=ba62/PLOSOne_formatting_sample_title_authors_affiliations.pdf

We have addressed these requirements. We are happy to address anything that we may have missed.

2. Thank you for stating the following in the Competing Interests section:

"TW consults for Color Genomics. Otherwise, the authors declare that they have no competing interests."

Please confirm that this does not alter your adherence to all PLOS ONE policies on sharing data and materials, by including the following statement: "This does not alter our adherence to PLOS ONE

policies on sharing data and materials." (as detailed online in our guide for authors http://journals.plos.org/plosone/s/competing-interests). If there are restrictions on sharing of data and/or materials, please state these. Please note that we cannot proceed with consideration of your article until this information has been declared.

We have added the phrase, "This does not alter our adherence to PLOS ONE policies on sharing data and materials," to our Competing Interests section.

Please respond by return email with your amended Competing Interests Statement and we will change the online submission form on your behalf.

Please know it is PLOS ONE policy for corresponding authors to declare, on behalf of all authors, all potential competing interests for the purposes of transparency. PLOS defines a competing interest as anything that interferes with, or could reasonably be perceived as interfering with, the full and objective presentation, peer review, editorial decision-making, or publication of research or non-research articles submitted to one of the journals. Competing interests can be financial or non-financial, professional, or personal. Competing interests can arise in relationship to an organization or another person. Please follow this link to our website for more details on competing interests: http://journals.plos.org/plosone/s/competing-interests

We have responded by email with our amended Competing Interests statement.

3. We note that you have indicated that data from this study are available upon request. PLOS only allows data to be available upon request if there are legal or ethical restrictions on sharing data publicly. For information on unacceptable data access restrictions, please see http://journals.plos.org/plosone/s/data-availability#loc-unacceptable-data-access-restrictions.

In your revised cover letter, please address the following prompts:

a) If there are ethical or legal restrictions on sharing a de-identified data set, please explain them in detail (e.g., data contain potentially identifying or sensitive patient information) and who has imposed them (e.g., an ethics committee). Please also provide contact information for a data access committee, ethics committee, or other institutional body to which data requests may be sent.

b) If there are no restrictions, please upload the minimal anonymized data set necessary to replicate your study findings as either Supporting Information files or to a stable, public repository and provide us with the relevant URLs, DOIs, or accession numbers. Please see http://www.bmj.com/content/340/bmj.c181.long for guidelines on how to de-identify and prepare clinical data for publication. For a list of acceptable repositories, please see http://journals.plos.org/plosone/s/data-availability#loc-recommended-repositories.

Our analysis uses germline-variant data from The Cancer Genome Atlas (TCGA) human cohort. The TCGA Human Subjects Protection and Data Access Policies state the following: "The controlled-access data tier

will not be freely available to the public, but will be made available to any qualified researcher for the purpose of biomedical research, once the investigator, along with his/her institution, has certified agreement to the statements within TCGA Data Use Certification (DUC). The data types in the controlled access tier include...individual-level germline variant data." (https://www.cancer.gov/about-nci/organization/ccg/research/structural-genomics/tcga/history/policies/tcga-human-subjects-data-policies.pdf) In addition, this page provides information about how researchers may contact the TCGA data-access committee with any questions. Accordingly, researchers can obtain germline variant data for the TCGA breast-cancer cohort, as we did. To make re-analysis more convenient for other researchers, we would be happy to share our summarized, filtered variant calls with any researcher who requests them, as long as the researcher has obtained approval to access TCGA controlled data. We have not and will not place any restrictions on data access beyond those of the TCGA data-access committee. Such restrictions are common for germline data. Accordingly, we believe we are in compliance with PLOS policies regarding data sharing.

We will update your Data Availability statement on your behalf to reflect the information you provide.

Thank you for updating this statement on our behalf. Please let me know if I can provide any other useful information regarding data availability.

4. Your ethics statement must appear in the Methods section of your manuscript. If your ethics statement is written in any section besides the Methods, please move it to the Methods section and delete it from any other section. Please also ensure that your ethics statement is included in your manuscript, as the ethics section of your online submission will not be published alongside your manuscript.

We have moved our ethics statement to the Methods section.

5. Please include captions for your Supporting Information files at the end of your manuscript, and update any in-text citations to match accordingly. Please see our Supporting Information guidelines for more information: http://journals.plos.org/plosone/s/supporting-information.

We have moved the captions for the Supporting Information files to the end of our manuscript and updated the in-text citations to match them.

6. We note that currently the supporting figures in your supporting information file "BRCAness_Supplementary.docx" are not displaying. Can you please ensure all the supporting figures are included and display clearly?

Thank you, and we apologize. We have fixed this problem and now provide these figures and tables as separate files to avoid further complications.

Reviewers' comments:

Reviewer's Responses to Questions

Comments to the Author

1. Is the manuscript technically sound, and do the data support the conclusions?

The manuscript must describe a technically sound piece of scientific research with data that supports the conclusions. Experiments must have been conducted rigorously, with appropriate controls, replication, and sample sizes. The conclusions must be drawn appropriately based on the data presented.

Reviewer #1: Yes

Reviewer #2: Yes

Thank you.

2. Has the statistical analysis been performed appropriately and rigorously?

Reviewer #1: Yes

Reviewer #2: Yes

Thank you.

3. Have the authors made all data underlying the findings in their manuscript fully available?

The PLOS Data policy requires authors to make all data underlying the findings described in their manuscript fully available without restriction, with rare exception (please refer to the Data Availability Statement in the manuscript PDF file). The data should be provided as part of the manuscript or its supporting information, or deposited to a public repository. For example, in addition to summary statistics, the data points behind means, medians and variance measures should be available. If there are restrictions on publicly sharing data—e.g. participant privacy or use of data from a third party—those must be specified.

Reviewer #1: No

Reviewer #2: Yes

Our analysis uses germline-variant data from The Cancer Genome Atlas (TCGA) human cohort. The TCGA Human Subjects Protection and Data Access Policies state the following: "The controlled-access data tier will not be freely available to the public, but will be made available to any qualified researcher for the purpose of biomedical research, once the investigator, along with his/her institution, has certified

agreement to the statements within TCGA Data Use Certification (DUC). The data types in the controlled access tier include...individual-level germline variant data." (https://www.cancer.gov/about-nci/organization/ccg/research/structural-genomics/tcga/history/policies/tcga-human-subjects-data-policies.pdf) In addition, this page provides information about how researchers may contact the TCGA data-access committee with any questions. Accordingly, researchers can obtain germline variant data for the TCGA breast-cancer cohort, as we did. To make re-analysis more convenient for other researchers, we would be happy to share our summarized, filtered variant calls with any researcher who requests them, as long as the researcher has obtained approval to access TCGA controlled data (in general). We have not and will not place any restrictions on data access beyond those of the TCGA data-access committee. Such restrictions are common for germline data. Accordingly, we believe we are in compliance with PLOS policies regarding data sharing.

4. Is the manuscript presented in an intelligible fashion and written in standard English?

PLOS ONE does not copyedit accepted manuscripts, so the language in submitted articles must be clear, correct, and unambiguous. Any typographical or grammatical errors should be corrected at revision, so please note any specific errors here.

Reviewer #1: Yes

Reviewer #2: Yes

Thank you.

5. Review Comments to the Author

Please use the space provided to explain your answers to the questions above. You may also include additional comments for the author, including concerns about dual publication, research ethics, or publication ethics. (Please upload your review as an attachment if it exceeds 20,000 characters)

Reviewer #1: The authors have proved their findings by a bioinformatical and statistical analysis of several genetic samples obtained by public accessible databases. The statistical methods used seem appropriate for their conclusion and the statistical power coming from the samples considerated seems enough to prove their point.

We thank the reviewer for taking the time to review the article and for providing a timely response!

1) My concern is that I was totally unable to visualize the supplementary figures and therefore I was unable to appropriately review the data discussed. I do not know if it was just my problem, but I would like to considerate also the supplementary figure before accepting the paper for publish.

It was a technical glitch that we, as the authors, should have prevented. We apologize. In the current version of the manuscript, we have uploaded these figures and tables as separate files to avoid such

problems.

2) In addition, I think that the method section needs more details and less references to other paper in order to allow the reader to reproduce the experiments and the analysis performed.

Thank you for this suggestion. We have added methodological details to various paragraphs throughout the Methods section. In place of prior sentences that referred the reader to referenced papers for these details, we have provided more details so that the reader can understand our approach without necessarily needing to read those other papers. Additionally, as before, we have provided a GitHub repository and an Open Science Framework repository that provide code that we used as an additional reference in support of reproducibility.

3) It would be interesting to see if applying their method to other candidate BRCAness genes such as the ones discovered by Konstantinopoulos et al (PMID 20547991), they would also fit into the group generated in this paper. Of course, if enough data are accessible from genetic databases.

Konstantinopoulos, et al. identified a signature of 60 genes that they used to classify patients into BRCA-like and non-BRCA-like categories based on expression levels of those genes. The reviewer suggested that these genes might also be considered as candidate BRCAness genes. However, to do this properly, we would need to have germline mutation status for these genes, but our process for determining pathogenicity of germline mutations is limited to the candidate genes that we evaluated in the paper, so I'm afraid it would not be a comprehensive comparison. Furthermore, the findings of Konstantinopoulos, et al. do not necessarily suggest that DNA mutations, copy-number variations, or hypermethylation of these genes are drivers of BRCAness; rather their findings suggest that these genes can be used to categorize patients into the BRCAness category, irrespective of the underlying driver events. Konstantinopoulos, et al. showed that their expression signature was successful at predicting response to a platinum agent and a PARP-inhibitor in samples relevant to epithelial ovarian cancer. We attempted to test this signature using the limited drug-sensitivity data available for the breast-cancer patients in TCGA. However, we had drug-sensitivity data for a single platinum agent (Carboplatin) and for no PARP inhibitors. For Carboplatin, we had data for 9 patients but only 2 non-responders, which was an insufficient sample size for building a predictive model. We hope that in the future, it will be possible to do more analysis with drug sensitivity data.

Reviewer #2: In the present paper Bodily et al. present an analysis of molecular profiles of breast cancer data retrieved from TCGA in order to identify genes that can be associated with BRCAness. Firstly they analyzed data of patients carrying BRCA1/2 germline mutation to define the molecular features in term of somatic mutational signature and expression profile. Then they analyzed molecular profiles of breast cancer patients carrying somatic BRCA1/2 alteration (promoter methylation, somatic mutation, homozygous deletion). They observed that BRCA1/2 germiline and somatic carriers have homogenous mutational signature and expression profile so they use all group as reference group to evaluate whether molecular aberrations in cancer–predisposing genes determine mutational signatures similar to breast cancer carrying BRCA1/2 alterations.
Genomic approaches are very important to address fundamental biological questions of breast cancer

and in particular, new studies to better define BRCAness are necessary for the identification of new therapeutic approaches. The approach used in the manuscript is intriguing and has the potential to positively influence the field of BRCAness. In my opinion however, the manuscript contains critical pitfalls that renders it unsuitable for publication in the present form. I listed below comments.

We thank the reviewer for taking the time to review the article and for providing a timely response!

Major comments

1) Supplementary data: The figures of supplementary file could not be opened with Word of Office (I tried with different version). I was able to open them with LibreOffice. Unfortunately supplementary tables were not even seen with LibreOffice.

It was a technical glitch that we, as the authors, should have prevented. We apologize. In the current version of the manuscript, we have uploaded these figures and tables as separate files to avoid such problems.

2) Line 40: “suggests additional genes”. What genes? ATR and BARD1? Are really new? Too vague sentence. In the paper of Lord and Asworth 2016 (BRCAness revisted) ATR is already included among BRCAness genes.

The reviewer makes an important point. Earlier in the abstract, we mention ATR and BARD1 as well as other genes that "showed high similarity but only for a small number of events or for a single event type." We have clarified this part to specifically mention the genes to which we were referring. Having clarified this part, we revised the final sentence so that instead of reiterating which genes were significant and suggesting that they might be considered for inclusion in the BRCAness definition (some of which already have been, as the reviewer notes), we emphasize that our "methodology represents an objective way to identify genes that have similar downstream effects on molecular signatures when mutated, deleted, or hypermethylated."

3) Section methods “data preparation and filtering” should be divided into paragraphs to distinguish how the authors analyzed the several molecular features.

We have divided this section into additional paragraphs to make the section more readable.

4) Line 120-121: The description of selection parameter for somatic mutation is unclear. Instead of “we used following criteria to exclude somatic variant” I would say somatic variants that are 1)… 2) were excluded

Thank you. We have made this change.

5) Also for results section the author did not divided into paragraphs. In this way different results are difficult to follow and the findings relative to each approach is lost during the reading. In the first part of results (line 204-215) the authors have written down an outline of the analysis they have done but on the followings paragraphs there is a mix. For instance homogeneity is treated at line 239-244 and then

at line 251-258. Since the conclusion is that BRCA1 and BRCA2 germline carriers show homogenous somatic mutation signature and expression profile, it would result clear to join the two paragraph and add a title. Paragraphs with titles would help the reader to understand the results.

Thank you for these helpful observations. We have reorganized the text and added two figures to help convey the results in a more logical and organized manner. Below we provide additional responses to these suggestions.

The fragmentation of results is also found in the figures. In line 217 is described Figure1A while the other panels of figure 1 are described in line 265. In my opinion, is easier to understand the paper if the panels of the figure are described in the same paragraph.

After reorganizing the Results section, the references to Figure 1 are now in the same paragraph.

lines 226-238: It is not clearly specified if this analysis is for all breast cancer (1101 patients) or for BRCA1/2 germline carries. It seems that this analysis refers to all patients. Why is it described after the analysis of germlines BRCA1/2? May be it is better as first paragraph.

We have clarified that this applies to all breast-cancer patients in the cohort and have added two figures that illustrate these data across all patients.

I would group the results in this way adding the following paragraphs (titles are just an example):
Aberration in BRCA1 and BRCA2
Line 216-225
Line 245-250
Line 264-277
Expression profile and signature of breast cancer
lines 226-238
Homogeneity of somatic mutation signature and expression profile of germline BRCA1/2 carriers
lines 239-244; 251-258.
Similarity between BRCA1/2 germline carriers
lines 259-262
Aberration in cancer predisposing genes
Lines 283-303
Line 305-318

We followed the reviewer's advice, although we have used somewhat different section names and have structured the subsections slightly differently than what the reviewer recommended. But we feel that our changes address the spirit of what the reviewer recommended.

6) Discussion: The discussion section overall lacks references and in some parts is a mere description of results. For instance how can be explained affirmation of lines 327-331? What has it been shown in previous papers? What is the impact of result of paper on BRCAness definition? Could the result help in

finding new therapeutic approaches?

Thank you for these suggestions. We have rewritten the Discussion section substantially in response to the reviewer's comments and questions. We have removed parts that were mere descriptions of results, and we have added citations in this section. We now provide some commentary on the utility of our approach for helping to clarify the definition of BRCAness and potentially to better understand mechanisms of BRCAness.

7) Line 369: which are the new factors highlighted? Explain better. What do you mean for factors? The two genes identified? Are they really new?

Thank you for pointing this out. Our wording was too vague. As mentioned above, we have rewritten the Discussion section to provide more thorough descriptions of prior evidence associated with our results. As part of this, we are now more explicit about the literature surrounding *BARD1* and *ATR*, in particular.

Minor comments:
1) Line 31 After "somatic-mutation signatures of tumors having" I would add molecular aberrations such as ….

Thank you. We have made this change.

2) Lane 170 extreme values instead of extremevalues

The name of the package is "extremevalues" without any spaces.

3) Line 293 for 11 genes …add the list of genes to guide the reader in the analysis of the table

Thank you. We have made this change.

6. PLOS authors have the option to publish the peer review history of their article (what does this mean?). If published, this will include your full peer review and any attached files. If you choose "no", your identity will remain anonymous but your review may still be made public. Do you want your identity to be public for this peer review? For information about this choice, including consent withdrawal, please see our Privacy Policy.

Reviewer #1: No

Reviewer #2: No

Thank you.

While revising your submission, please upload your figure files to the Preflight Analysis and Conversion Engine (PACE) digital diagnostic tool, https://pacev2.apexcovantage.com/. PACE helps ensure that

figures meet PLOS requirements. To use PACE, you must first register as a user. Registration is free. Then, login and navigate to the UPLOAD tab, where you will find detailed instructions on how to use the tool. If you encounter any issues or have any questions when using PACE, please email PLOS at figures@plos.org. Please note that Supporting Information files do not need this step.

We have performed this check. PACE reported no issues. In our resubmission, we used the TIFF files created by PACE for the non-supplementary figures.
